# The dentin phosphoprotein repeat region and inherited defects of dentin

**DOI:** 10.1002/mgg3.176

**Published:** 2015-09-07

**Authors:** Jie Yang, Kazuhiko Kawasaki, Moses Lee, Bryan M. Reid, Stephanie M. Nunez, Murim Choi, Figen Seymen, Mine Koruyucu, Yelda Kasimoglu, Ninna Estrella‐Yuson, Brent P. J. Lin, James P. Simmer, Jan C.‐C. Hu

**Affiliations:** ^1^ Department of Biologic and Materials SciencesUniversity of Michigan School of Dentistry1210 Eisenhower PlaceAnn ArborMichigan; ^2^ Department of Pediatric DentistrySchool and Hospital of StomatologyPeking University22 South AvenueZhongguancun Haidian DistrictBeijing100081China; ^3^ Department of AnthropologyPennsylvania State UniversityUniversity ParkPennsylvania16802; ^4^ Department of Biomedical SciencesSeoul National University College of Medicine275‐1 Yongon‐dong, Chongno‐guSeoul110‐768Korea; ^5^ Department of PedodonticsFaculty of DentistryIstanbul UniversityIstanbulTurkey; ^6^ Department of Paediatric DentistryWomen's and Children's Hospital72 King William RoadNorth AdelaideSouth Australia5006Australia; ^7^ Department of Pediatric DentistrySchool of DentistryUniversity of CaliforniaSan FranciscoCalifornia

**Keywords:** Mutations, osteogenesis imperfecta, SMRT technology, tooth, whole‐exome sequencing

## Abstract

Nonsyndromic dentin defects classified as type II dentin dysplasia and types II and III dentinogenesis imperfecta are caused by mutations in *DSPP* (dentin sialophosphoprotein). Most reported disease‐causing *DSPP* mutations occur within the repetitive DPP (dentin phosphoprotein) coding sequence. We characterized the DPP sequences of five probands with inherited dentin defects using single molecule real‐time (SMRT) DNA sequencing. Eight of the 10 sequences matched previously reported DPP length haplotypes and two were novel. Alignment with known DPP sequences showed 32 indels arranged in 36 different patterns. Sixteen of the 32 indels were not represented in more than one haplotype. The 25 haplotypes with confirmed indels were aligned to generate a tree that describes how the length variations might have evolved. Some indels were independently generated in multiple lines. A previously reported disease‐causing *DSPP* mutation in Family 1 was confirmed and its position clarified (c.3135delC; p.Ser1045Argfs*269). A novel frameshift mutation (c.3504_3508dup; p.Asp1170Alafs*146) caused the dentin defects in Family 2. A *COL1A2* (c.2027G>A or p.Gly676Asp) missense mutation, discovered by whole‐exome sequencing, caused the dentin defects in Family 3. We conclude that SMRT sequencing characterizes the DPP repeat region without cloning and can improve our understanding of normal and pathological length variations in *DSPP* alleles.

## Introduction

Nonsyndromic inherited dentin defects are relatively common, with an incidence of one in every 6000 to 8000 persons (Witkop 1957; Aplin et al. 1995). Inherited dentin defects, subclassified as dentin dysplasia type II (DD‐II) and dentinogenesis imperfecta type II and type III (DGI‐II, DGI‐III) (Shields et al. 1973), show an autosomal dominant pattern of inheritance and are all caused by mutations in dentin sialophosphoprotein (*DSPP*; OMIM*125485) (Kim and Simmer 2007). *DSPP* is a member of the secretory calcium‐binding phosphoprotein (SCPP) gene family, which all share a common 5′ region that is descended from an ancestral SPARC‐like 1 gene (Kawasaki et al. 2004). There are two SCPP subfamilies: the proline/glutamine (P/Q) rich and the acidic genes. In humans there are 18 P/Q‐rich and 5 acidic SCPP genes. All except *AMELX* (*300391) and *AMELY* (*410000) are clustered on chromosome 4. *DSPP* is a member of the acidic group (also called SIBLINGs or Small Integrin‐Binding LIgand, N‐linked Glycoproteins) (Fisher et al. 2001). Features shared by SCPP genes include a conserved signal peptide and signal peptide cleavage site, a short N‐terminal motif that facilitates trafficking through the secretory pathway (Nam et al. 2014), a near N‐terminal Golgi casein kinase (G‐CK) phosphorylation site (Ser‐X‐Glu or Ser‐X‐pSer; SXE/pS), and phase zero introns (Kawasaki and Weiss 2003).

*DSPP* is the most recent member of the acidic SCPP genes to be generated during evolution. *DSPP* is found in mammals and reptiles, but not in amphibians or fish, whereas the other acidic SCPP genes appeared earlier: *DMP1* (*600980), *IBSP* (*147563), and *SPP1* (*166490) are in the Coelacanth (a fish with lobefins and lungs) and *MEPE* is in amphibians. *DSPP* appears to have originated as a duplication of *Dspp like 1* (*Dsppl1*), which is found in Coelacanth and in reptiles, but not in mammals (Kawasaki and Amemiya 2013). *Dsppl1* may serve the same function as *DSPP*. When *Dsppl1* duplicated, *DSPP* landed on the other side of *DMP1* within the acidic SCPP gene cluster, and *Dsppl1* was subsequently deleted during the evolution of mammals. Coelacanth *Dsppl1* sequence shows conservation in its 5′ regulatory region with mammalian *DSPP* (Fig. S1), which has been studied in mouse and contains important transcriptional *cis*‐regulatory elements (Feng et al. 1998; Chen et al. 2004).

*DSPP* expression is tooth‐specific (Chen et al. 2004). No *DSPP* expressed sequence tags (ESTs) are listed among the 3,328,811 characterized from over 40 healthy tissues (excluding developing teeth) in the human EST file for *DSPP* (Hs.678914). DSPP is required for normal dentin formation in mammals, as *Dspp* null mice exhibit severe dentin malformations (Sreenath et al. 2003). *Dspp* is pseudogenized in tetrapods that have lost the ability to make teeth during evolution, including anteaters (McKnight and Fisher 2009), turtles (Shaffer et al. 2013), and birds (Kawasaki and Amemiya 2013). These findings support the conclusion that DSPP is specialized for the formation of dentin.

Studies of DSPP protein products isolated from developing porcine and murine dentin have contributed much to our understanding of its structure and posttranslational modifications. Porcine DSPP is secreted as a single protein that is cleaved following its secretion (Tsuchiya et al. 2011). Uncleaved DSPP has been detected in mouse dentin (Sun et al. 2010). The first DSPP cleavage releases dentin phosphoprotein (DPP) from the C‐terminus. This cleavage is catalyzed by bone morphogenetic protein 1 (BMP1) and related proteases (von Marschall and Fisher 2010; Sun et al. 2010; Tsuchiya et al. 2011). Following the release of DPP from the parent DSPP protein, there is a subsequent cleavage that generates dentin glycoprotein (DGP), an 81‐amino acid domain with four phosphoserines and one N‐linked glycosylation (Yamakoshi et al. 2005b). The cleavage that generates DGP is catalyzed by MMP20 (Yamakoshi et al. 2006) and has not been demonstrated to occur in other animal models besides pig. The amount of DGP extracted from pig dentin was reduced by rapid acid extraction, suggesting that some (but not all) of the accumulated DGP was a postmortem artifact. The N‐terminal domain of DSPP is dentin sialoprotein (DSP), a proteoglycan that binds hyaluronic acid, and forms covalent dimers via a disulfide bridge at Cys^205^ (the only cysteine in DSPP). This cysteine is conserved in mammalian DSP sequences (McKnight and Fisher 2009). Porcine DSP has two glycan attachments, at Ser^253^ and Ser^265^. Both comprised chondroitin‐6‐sulfate (Yamakoshi et al. 2005a; Yamakoshi 2009). Mouse DSP also has two glycan attachments, which contain chondroitin‐4‐sulfate (Zhu et al. 2010). Porcine DSP has at least six *N*‐glycosylations (at Asn^52^, Asn^92^, Asn^151^, Asn^170^, Asn^176^, and Asn^191^) (Yamakoshi et al. 2011). Most DSP oligosaccharide attachments are removed by chondroitinase ABC digestion, indicating that the glycans are the predominant posttranslational modification of DSP (Yamakoshi et al. 2005a). Overall the primary structure of porcine DSPP (Yamakoshi et al. 2008) is: Signal peptide (Met^1^ to Ala^15^) DSP (Ile^16^ to Arg^391^) DGP (Ser^392^ to Gly^472^) DPP (Asp^473^ to Asp^1066^). The primary structure of human DSPP is: Signal peptide (Met^1^ to Ala^15^) DSP (Ile^16^ to Gly^462^) DPP (Asp^463^ to Asp^1301^).

The size of the DPP domain of DSPP varies from species to species and even among individuals within a species. Porcine DPP coding region length polymorphisms ranged from 3 to 63 nucleotides in length and appeared in at least four unique patterns (haplotypes) and expressed DPP domains between 551 and 594 amino acids (Yamakoshi et al. 2008). Human DPP varies between 770 and 902 amino acids (Wang et al. 2011). The number of amino acids in the DPP reference sequences from selected mammals are humans 839, mouse 494, and aardvark 524. These length variations reflect the size of a repetitive region, which includes numerous Asp‐Ser‐Ser (DSS) sequence motifs. There are 165 DSS sequences in human DPP. The amino acid composition of human DPP is 60% serine and 26% aspartic acid.

DPP is highly phosphorylated. Assuming the DPP molecular mass to be 100‐kDa, porcine DPP averaged 212 phosphates per molecule. The calculated isoelectric point of unmodified human DPP is 2.84 and its deduced molecular weight is 82‐kDa. In contrast, the measured pI of the rat DPP (modified) protein was 1.1 (Jonsson and Fredriksson 1978) and the apparent molecular weight of human DPP on sodium dodecyl sulfate polyacrylamide gel electrophoresis (SDS‐PAGE) is 140‐kDa (Chang et al. 1996). There are only eight primary SXE G‐CK sites in human DPP and phosphorylation of these serines generates six secondary SXpS G‐CK sites. The total of 14 G‐CK sites is far below the number of phosphoserines on human DPP, based upon the data from pig (Yamakoshi et al. 2008). There must either be other (unidentified) kinases in the secretory pathway that can phosphorylate DPP or G‐CK can also phosphorylate Ser‐X‐Asp sites. Recently G‐CK was shown to be part of a secretory kinase complex that regulates extracellular protein phosphorylation (Cui et al. 2015). A pseudokinase encoded by *Fam20a* increases the activity of G‐CK, which is encoded by *Fam20c* (Tagliabracci et al. 2012). Perhaps other factors can influence the target‐site specificity of the kinase complex in a way that could account for the observed phosphorylation of DPP.

Sequencing through the DPP repetitive region is necessary to determine the genetic causes of inherited dentin defects and their pathogenesis, to understand the range of DPP length variations in humans, to provide information from a gene on an autosomal chromosome for human migration studies, and to determine the DPP sequences of species whose genomes are being characterized by next‐generation sequencing (which cannot assemble the correct DPP sequence) to better understand the evolution of *Dspp* and its role in biomineralization. Until now, the only way to sequence the DPP repetitive region was to clone PCR amplification products and use the resulting plasmid DNA as template (McKnight et al. 2008b), which is labor intensive and impractical for most applications, including diagnoses. Here, we use the single molecule real‐time (SMRT) DNA sequencing system to accurately characterize the human DPP repetitive region without cloning, identify a novel frameshift mutation that causes DGI‐II, and analyze the currently known human DPP length variations to establish their relations to each other.

## Materials and Methods

The human study protocol and consents were reviewed and approved by Institutional Review Board (IRB) Committees at the University of Michigan, the University of Texas Health Science Center at San Antonio, and the Ethics Committee at the University of Istanbul, Turkey. Study participants signed appropriate written consents after explanation and discussion of their contents. Minors age 8 or older signed a written assent form after their parents completed a written parental consent form for participation of the minor.

### Recruitment of subjects

Five unrelated families with at least one person who had been diagnosed by their dentists as having inherited dentin defects were enrolled in our study. Family medical and dental histories were obtained and the family pedigrees were ascertained. The dental phenotypes of recruited family members were documented following clinical and radiographic examinations. Family 1 was a positive control with a known frameshift muation in the DPP repetitive region (McKnight et al. 2008a).

### Genomic DNA extraction

Peripheral whole blood (5 cc) or saliva (2 cc) were obtained from enrolled subjects and genomic DNA was isolated using the QIAamp DNA Blood Maxi Kit (51194; Qiagen, Valencia, CA) or Saliva DNA Collection, Preservation and Isolation Kit (RU35700; Norgen Biotek Corporation; Thorold, Canada), respectively. The quality and quantity of the extracted DNA samples was determined by gel electrophoresis and spectrophotometry at OD_260_ and OD_280_.

### Whole‐exome analyses

DNA samples from families 2, 3, and 5 were subjected to whole‐exome sequencing (WES) at the Yale Center for Genome Analysis (West Haven, CT) using specific approaches modified according to a previous report (Choi et al. 2009). Briefly, the genomic DNA was captured with NimbleGen v2.0 exome capture reagent (Roche/NimbleGen Incorporation; Madison, WI) and sequenced with Illumina, San Diego, CA, USA, HiSeq2000 for 75 base paired end reads. Reads were aligned to human reference genome hg19 using ELANDv2. Single‐nucleotide variants and short insertions and deletions (indels) were called using SAM tools and annotated using an in‐house script. The annotated results were first searched for potential disease‐causing sequence variations in the nonredundant region of *DSPP* and in known candidate genes for osteogenesis imperfecta (OI): *COL1A1*,* COL1A2*,* IFITM5, FKBP10, CRTAP, LEPRE1, PPIB, LRP5, PLOD2, FKBP10, SERPINF1, SERPINH1*,* WNT1*,* BMP1*,* PLS3*,* TMEM38B, CBP2,* and *SP7* (Valadares et al. 2014).

The number of reads per exome ranged from 70 to 94 mol/L. The mean coverage of the exome ranged from 67X to 93X. The quality of the WES data is summarized in Table S1. Based on the WES results, exon 34 of the type I collagen gene, collagen type I alpha 2 (*COL1A2*), was amplified using genomic DNA from the participants in Family 3 as template. The 229 bp amplification product was purified and characterized by direct Sanger sequencing at the University of Michigan DNA Sequencing Core (Ann Arbor, MI). For Family 4, which did not consent to WES, genomic DNA from the proband was first evaluated for sequence variations in the *DSPP* nonrepetitive coding region by Sanger sequencing of PCR amplification products. Oligonucleotide primer sequences and the reaction conditions for all PCR amplification performed in this study are provided in Table S2.

### SMRT sequencing and analysis

SMRT sequences were characterized in two single cells by using PCR amplification primers that included 5‐prime, 16‐basepair “barcode” sequences that were unique for each proband on their 5′ ends (Table S2). The barcodes were selected from published data (http://www.smrtcommunity.com/Share/Protocol?id=a1q70000000J4m5AAC). The barcoded primer pairs used to amplify the DPP repetitive region annealed at sites that would produce an amplification product ~2543 bp in length if the *DSPP* gene haplotype in the patient was the *DSPP* reference sequence (NM_014208.3). As the *DSPP* sequence in the DPP coding region is variable in its length due to existence of multiple indels in the DPP repetitive region, most DPP amplifications generate two amplicons that are manifested as a thick band or doublet on an agarose gel. These amplicons were separated from primers by excising them from a 1.25% agarose gel stained with SYBR Gold nucleic acid gel stain (Life Technologies, Calsbad, CA) and purified using QIAquick Gel Extraction Kit (Qiagen, Hilden, DE). Purified amplicons were quantified using a spectrometer (Beckman, Carlsbad, CA, USA, Coulter DU730). An average of 250 ng of purified amplicons from each proband was submitted to the University of Michigan DNA Sequencing Core for SMRT sequencing using two SMRT cells P4‐C2 chemistry, 120 min movies and hot start. The sequence data were compiled by the Bioinformatic core and further interrogated by informaticians at the Pacific Biosciences (Menlos Park, CA).

### DPP alignment

Wild‐type DPP haplotype sequences were downloaded from NCBI (National Center for Biotechnology Information) PopSets 162077127 and 162077085 (Table S3A and B), which contain DPP haplotype sequences from two different sizes of cloned DPP PCR products (McKnight et al. 2008a). Other published DPP haplotype sequences that were not submitted to GenBank but had clearly described the indels with respect to the reference sequence (Song et al. 2008) were readily assembled. The DNA alignments were made manually, trying to minimize the number of genetic events required to generate the observed allelic differences in DPP sequences. Only a single previously published DPP sequence for each indel pattern was retained in the final alignment.

The nomenclatures for the 32 indels (Table S4) and the 21 known disease‐causing DPP frameshift mutations (Table S5) were verified using *Mutalyzer* 2.0.3 at http://www.lovd.nl/mutalyzer/ (Wildeman et al. 2008) and were described with respect to the *DSPP* reference sequence (NM_014208.3).

### Generating a tree relating the known DPP haplotypes

Indels found in only a single haplotype were eliminated from this analysis as potentially being PCR artifacts, which reduced the number of novel DPP haplotypes from 36 to 25. These “confirmed” indel patterns (haplotypes) were then analyzed to produce a tree showing a potential relationship of DPP haplotypes in human populations. The tree was generated using *Network* version 4.6.1.3 available from Fluxus Technology Ltd. (www.fluxus-engineering.com). The indels of each haplotype were converted into binary data and used to calculate a phylogenetic network using the median‐joining algorithm (Bandelt et al. 1999).

## Results

### Family 1

**Figure 1:**
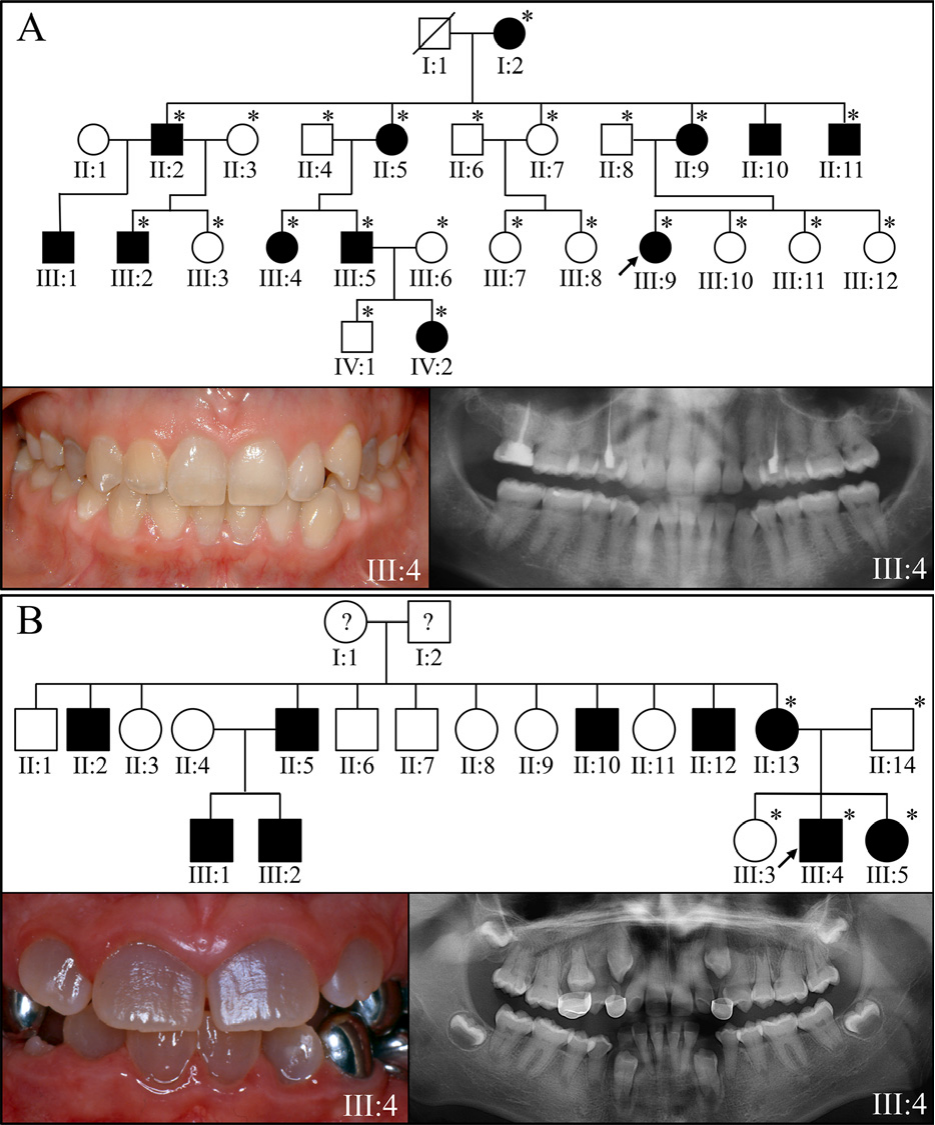
Pedigrees and proband's phenotype in families with *DSPP* −1 frameshifts. (A) Family 1: the four generation pedigree of a family diagnosed with dentin dysplasia type II following an autosomal dominant pattern of inheritance. The dental phenotype was mild, with grayish/yellowish discoloration of the permanent dentition. The panorex radiograph showed premature obliteration of the root canals, thistle‐shaped pulp chambers, and mildly bulbous molar crowns. The cause of this phenotype was confirmed by SMRT sequencing to be c.3135delC; p.Ser1045Argfs*269. This designation is, by convention, numbered as if the deletion had occurred in the *DSPP* reference sequence (NM_014208.3). (B) Family 2: the pedigree is consistent with an autosomal dominant pattern of inheritance. The proband presented with a more severe dental phenotype than the proband of Family 1, with grayish discoloration of the crowns, bulbous crown morphology with constricted cervical areas of the roots, and obliterated pulp chambers and root canals. This phenotype was caused by a five nucleotide duplication in a single *DSPP* allele with haplotype SHap3 (c.3504_3508dup; p.Asp1170Alafs*146) that would have produced a −1 frameshift that is known to cause inherited dentin defects. An asterisk in the pedigree marks persons who were recruited for the study and contributed DNA samples. *DSPP*, dentin sialophosphoprotein; SMRT, single molecule real time.

Family 1 was a Caucasian family spanning four generations with DD‐II that followed an autosomal dominant pattern of inheritance (Fig. 1A). The dental phenotype of this family was described previously (Beattie et al. 2006). The primary dentition was more severely affected than the secondary dentition. The phenotype varied, but the crowns of the permanent dentition of affected individuals were shaded gray. The pulp chambers tended to fill‐in (obliterate) more rapidly than normal, and some posterior teeth had bulbous crowns. Penetrance appeared to be complete as more than half of the offspring of a single affected parent exhibited the DD‐II phenotype. The genetic cause was a variant of *DSPP* haplotype SHap6(2) that included a novel single‐nucleotide deletion originally described as c.3141delC or p.S1047fsX223 (McKnight et al. 2008a). Subsequently it was noted that the designation for this mutation was in error, as nucleotide 3141 in the *DSPP* reference sequence (NM_014208.3) was not a “C.” By comparing the published chromatogram with the DSPP cDNA reference sequence, an alternative designation was proposed: c.3153delC and p.S1051fsX223 (Wang et al. 2011). To clarify this situation and to help assess the reliability of SMRT DNA sequencing for accurately determining the sequence of the DPP repetitive region, we included DNA from Family 1 along with that of four other families exhibiting inherited dentin defects of unknown etiology to be characterized by SMRT sequencing. These sequences were analyzed by aligning them to the sequences of all known *DSPP* haplotypes that vary due to their pattern of indels (Fig. S2).

The proband of Family 1 carried two different *DSPP* alleles. The SMRT sequence for the first *DSPP* allele (designated F1a) showed the same indel pattern as haplotype SHap6(2) (Song et al. 2008). The 2399 basepair DPP sequence exactly matched the one previously published for this family (McKnight et al. 2008b), and included the single‐nucleotide deletion that caused the DD‐II phenotype. Aligning this sequence to other *DSPP* haplotypes (Fig. S2) resolved the ambiguity of the designation for the disease‐causing single‐nucleotide deletion. The previous confusion occurred because the position of the single‐nucleotide deletion was immediately upstream from an indel that deleted the subsequent 18 nucleotides relative to the *DSPP* reference sequence (NM_014208.3). The proper designation for the single‐nucleotide deletion is c.3135delC; p.Ser1045Argfs*269. A lesson learned is that properly designating a DPP mutation sometimes requires aligning the patient's sequence to the known DPP haplotypes (not just the reference sequence) to accurately position the defect. The second DSPP allele in the proband (F1b) had the same indel pattern as *DSPP* haplotype 1a (Hap1a) and showed no potential disease‐causing sequence variations.

### Family 2

Family 2 was a Hispanic family with a DGI phenotype following an autosomal dominant pattern of inheritance (Fig. 1B). The dental phenotype was more severe than in Family 1. The permanent teeth were shaded gray, the posterior teeth had bulbous crowns, and the pulp chambers obliterated prematurely. WES analyses found no potential disease‐causing mutations in the type I collagen genes (*COL1A1/COL1A2*), or the nonredundant region of *DSPP*. SMRT analyses provided the DPP sequences of both *DSPP* alleles in the proband. F2a had the same DPP indel pattern as SHap3 (Song et al. 2008) as well as a novel 5‐bp duplication in the DPP coding region of *DSPP* (c.3504_3508dup; p.Asp1170Alafs*146) that would have generated −1 reading frameshift of the type known to cause inherited dentin defects (McKnight et al. 2008b). This is the first novel disease‐causing *DSPP* mutation identified by SMRT sequencing and in this case helped to establish the diagnosis as being DGI‐II, rather than OI. The other DPP allele in the proband (F2b) was identical to the reference sequence except for a novel 18‐bp duplication (indel 11 or ID11).

### Family 3

**Figure 2:**
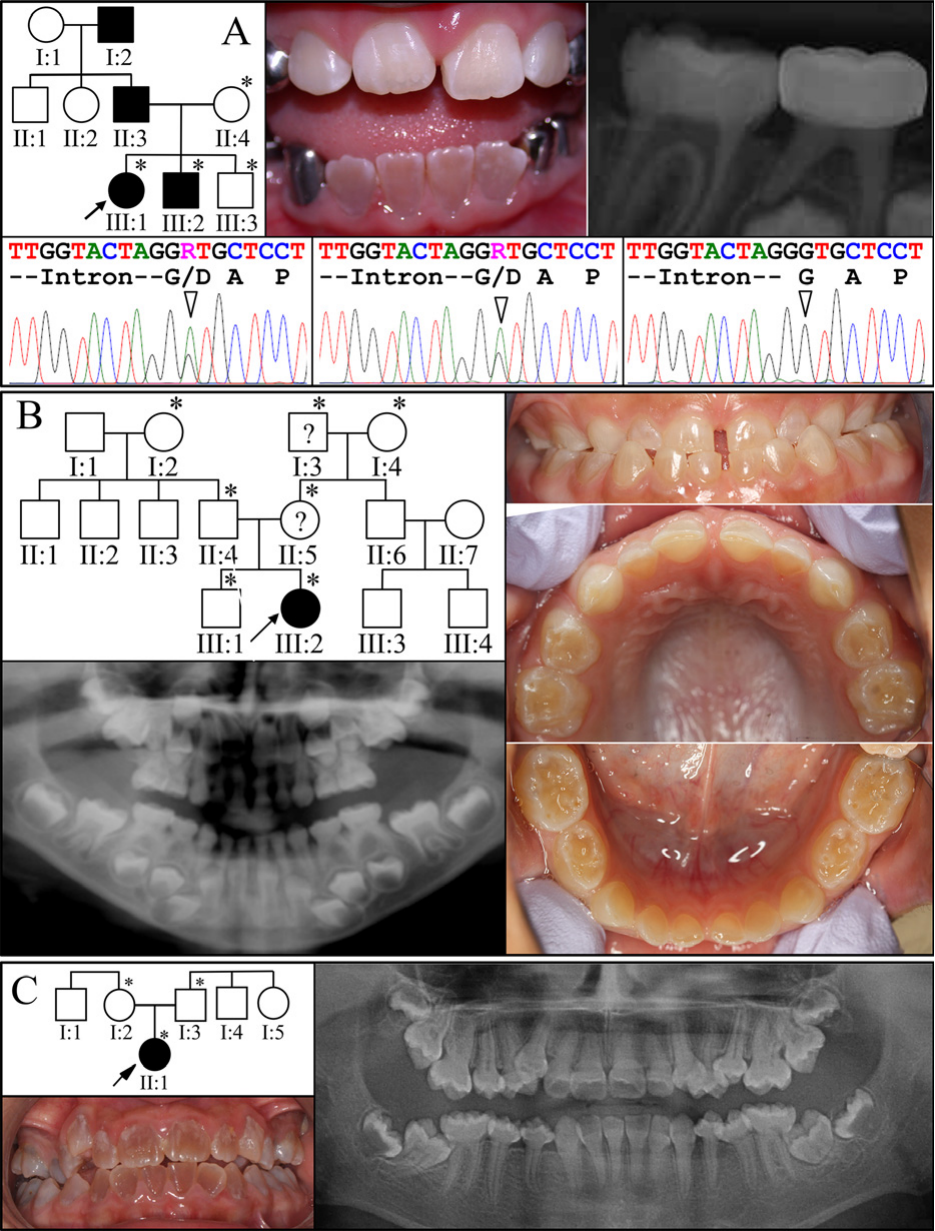
Pedigrees and proband's phenotype in OI families. (A) Family 3: the three generation pedigree of a family diagnosed with inherited dentin defects that follow an autosomal dominant pattern of inheritance. The dental phenotype was typical of dentinogenesis imperfecta, with variable discoloration of the permanent dentition, abnormal pulp size, and bulbous molar crown morphology. The cause of this phenotype was discovered during whole‐exome sequence analyses to be a dominant mutation in the first codon of exon 34 in *COL1A2*: c.2027G>A; p.Gly676Asp. Sanger sequencing chromatograms from exon 34 of *COL1A2* show that the proband (III:1; left) and her similarly affected younger brother (III:2; middle) were heterozygous for this mutation, while the unaffected brother (III:3; right) did not carry the mutant allele. (B) Family 4: mild dentin defects in the primary dentition of a simplex case. No potential disease‐causing mutations were detected in *DSPP*. (C) Family 5: a second simplex case of inherited dentin defects in the absence of any *DSPP* defects. The dentition is severely discolored, with bulbous crowns, constricted cervical areas, pulp obliteration, and thin narrow roots. No disease‐causing mutation was evident by the whole‐exome sequence analysis. An asterisk in the pedigree marks persons who were recruited for the study and contributed DNA samples. OI, osteogenesis imperfecta; *COL1A2*, collagen type I alpha 2; *DSPP*, dentin sialophosphoprotein.

Family 3 was a Hispanic family exhibiting an autosomal dominant DGI phenotype, with grayish, variably colored teeth, bulbous crowns, and abnormal pulp size, either larger or smaller than normal, and some pulp obliteration (Fig. 2A). The mother reported that proband was otherwise healthy, and denied bone fragility or joint pain. This was true at the time of enrollment (age 5.5 years), but the proband later broke her arm at age 7. WES analysis identified a heterozygous mutation in the first codon of exon 34 in *COL1A2*: c.2027G>A; p.Gly676Asp (based on *COL1A2* cDNA reference sequence NM_000089.3). This *COL1A2* mutation was previously reported to cause OI type III, but no description of the phenotype was provided and there was no mention of whether or not there were dentin defects (Lee et al. 2006). The DPP region of *DSPP* was characterized by SMRT sequencing. No potential disease‐causing mutations were observed in *DSPP*. The *COL1A2* defect established the diagnosis of OI and determined that the dentin defects were the earliest feature of the OI in this family. The DPP sequences determined by SMRT sequence analysis were identical to previously published haplotypes (F3a, Hap15a; F3b, Hap2a).

### Families 4 and 5

Family 4 was a Caucasian family with only the proband presenting with inherited dentin defects, which included yellowish discolored teeth, bulbous crowns, and obliterating pulp chambers (Fig. 2B). As the family did not consent to WES analyses, mutation analyses of *DSPP* (exons 1–4 and the 5′ part of exon 5; Table S2) and SMRT sequencing of the DPP region was performed, but did not identify any frame‐shift sequence variations. The proband's DPP alleles were a novel haplotype (F4a) and the *DSPP* reference sequence (F4b; SHap2). A c.2535C>A or p.Ser845Arg missense mutation was observed in one allele (F4a), which has a PolyPhen‐2 prediction of being probably damaging with a score of 0.993 (sensitivity 0.70; specificity 0.97) (Adzhubei et al. 2013). The cause of the dental phenotype in this family remains unknown.

Family 5, a Turkish family, was a sporadic case of OI type I with opalescent dentin and a history of bone fractures (Fig. 2C). Despite the confident clinical diagnosis, WES analyses did not find any deleterious variants in any of the OI candidate genes or in the DSP coding region. In addition, no potentially disease‐causing variants in the DPP coding region were identified by SMRT sequencing, which were identical to previously published haplotypes (F5a, Hap2a; F5b, Hap15a).

### DPP repetitive region

**Figure 3:**
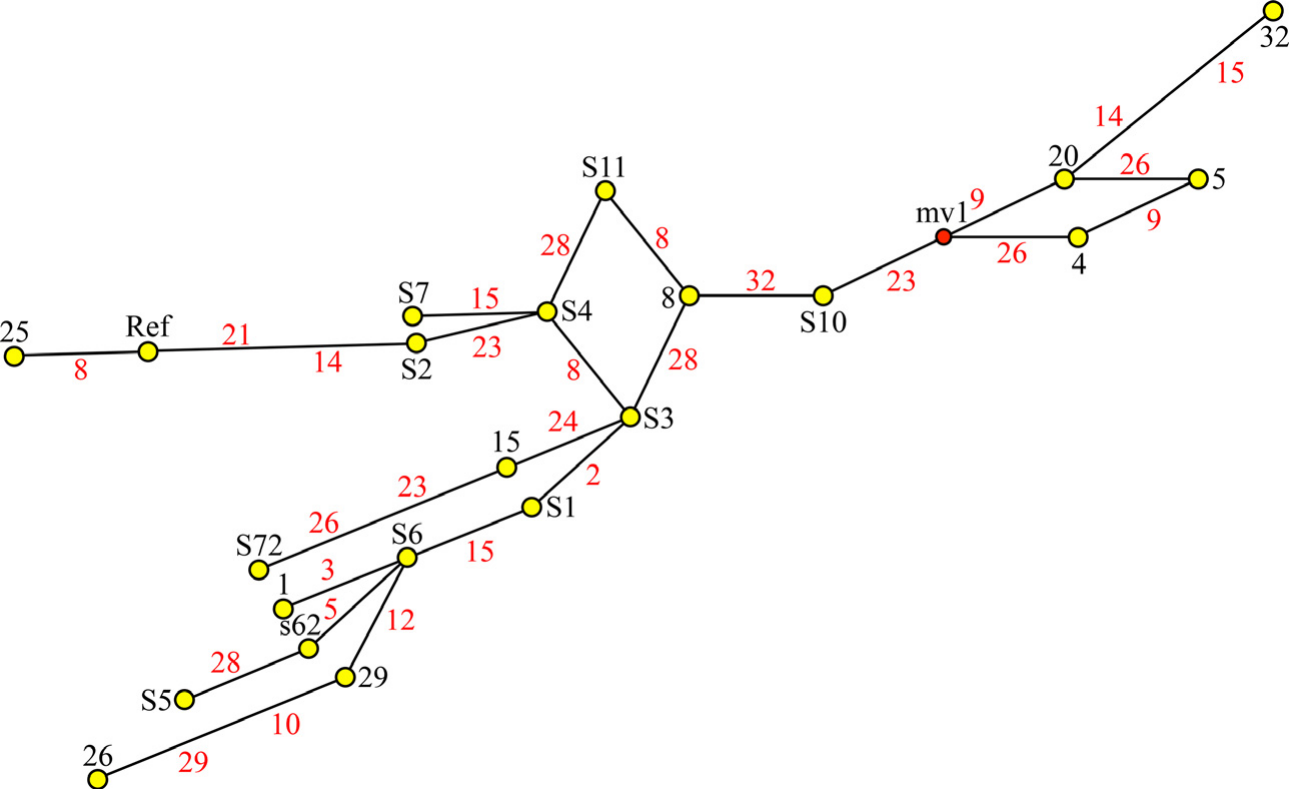
Tree describing a possible evolutionary relationship among the confirmed dentin phosphoprotein (DPP) length haplotypes (HAP). Yellow dots are nodes on the tree corresponding to a novel DPP length haplotype. The haplotypes are numbered in black type and “HAP” has been removed from the designations. Red numbers correspond to each of the confirmed DPP indels (those appearing in more than one unique length haplotype). Note that indels 8, 15, 23, 26, and 28 appear three times and indels 9 and 14 appear twice. DPP indels are apparent hot spots for slippage during DNA replication. A hypothetical haplotype, mv1 (medial vector 1, shown in a red dot), was inserted to construct the shortest connection.

**Figure 4:**
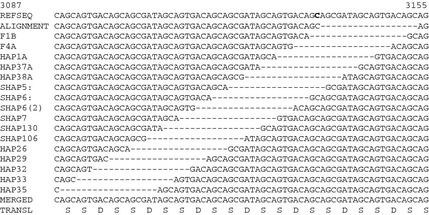
Variable positions for indel 15. The alignment covers the DPP sequence between ID14 and ID16. ID15 is an 18‐bp deletion relative to the reference sequence. Here, ID15 is placed in a different position each DPP length haplotype that contains it. ID15 could be placed at any position between ID14 and ID16. When aligning such sequences, Occam's razor is applied to minimize the number of genetic events required to explain the variation. As a result the dashes are aligned at a somewhat arbitrary location (as in Fig. S2), which suggests that the indel was generated at a single time and position and is present in multiple sequences through common descent. The tree, however, suggests that ID15 was generated independently in three lines. The C in bold is the site of a single bp deletion that caused DD‐II (c.3135delC). DPP, dentin phosphoprotein; DD‐II, dentin dysplasia type II.

The SMRT sequences of the DPP region for the 10 *DSPP* alleles of the probands from the five families with inherited dentin defects were aligned with the *DSPP* cDNA reference sequence (NM_014208.3), the McKnight sequence (EU709728) previously obtained for the DPP sequence of the proband of Family 1, and all previously reported *DSPP* haplotypes that varied with respect to their pattern of insertions or deletions (indels) in the DPP coding region (Fig. S2). The alignment shows 32 different indels in the DPP coding region relative to the *DSPP* reference sequence. The number of nucleotides in every indel is divisible by 3, so all maintain the reading frame and none are associated with a loss of function. The 32 indels in the DPP repetitive region combined into 36 different *DSPP* haplotypes based on the patterns of indels (Fig. S2). Half of the *DSPP* indels (16) have been identified in no more than one haplotype, and some of these may be artifacts generated during PCR amplification. If only the 16 shared indels (ones not unique to a single haplotype) are used and the 16 unconfirmed indels ignored, there are 25 unique patterns of DPP indels or *DSPP* length haplotypes (Fig. S3). A tree was generated mapping the relationships of unique *DSPP* length haplotypes (Fig. 3). The tree shows several of the indels occurred independently in multiple haplotypes. This is especially likely because some parts of the DPP repetitive region are so repetitive that the position of an indel can be moved up or down the sequence in the alignment (Fig. 4), so that independent insertions or deletions (indels) of the same length that occur within such a region would be aligned and appear to be generated in a single genetic event.

## Discussion

Next‐generation sequencing enables efficient examination of genomes except in repetitive areas that cannot be “disambiguated” during the assembly of short overlapping sequences. SMRT technology generates reads of several kilobases and has been used to close gaps in the human reference genome, 78% of which contains tandem repeats (Chaisson et al. 2015). The advantage of SMRT sequencing is that the target sequences are not fragmented into small segments of DNA that must contain overlapping unique sequences to accurately assemble the longer original sequence. Instead, the original DNA target sequences (in the case of DPP ~2500 bp) were circularized and sequenced as single molecules. Between 100 and 200 independently determined full‐length DPP sequences were aligned to eliminate random sequencing errors and to obtain a consensus sequence for each DPP allele. This platform allowed us to sequence through the highly redundant DPP coding region without cloning, determine the pattern of indels in both *DSPP* alleles in each proband, and to identify frameshift mutations in the DPP repetitive region (and the haplotype in which they occurred) that caused DD‐II and DGI‐II.

Type I collagen and DSPP are the principle organic constituents of tooth dentin (Linde 1989), and mutations in the genes encoding these proteins are the predominant cause of inherited dentin defects (Kim and Simmer 2007). *DSPP* is tooth‐specific, and defects in this gene cause isolated (nonsyndromic) dentin defects. Type I collagen is an important constituent in tissues besides dentin, such as bone, tendon, and skin, so mutations in the *COL1A1* or *COL1A2* genes (encoding type I collagen) cause dentin defects in syndromes, such as OI. Many genes contribute to the production of type I collagen, but due to their dominant pattern of inheritance, over 80% of the mutations that cause OI are in *COL1A1* or *COL1A2* (Kuivaniemi et al. 1997). Some patients with OI show no dentin abnormalities, while others suffer severe dentin malformations (O'Connell and Marini 1999). Some patients with type I collagen mutations show only dentin manifestations (Pallos et al. 2001; Wang et al. 2012).

In hereditary diseases of dentin, the primary teeth are typically more severely affected than the permanent dentition and therefore evident at an early age. Such patients are often diagnosed as having DD‐II, DGI‐II, or DGI‐III until an episode of bone fracture prompts a revision of the diagnosis to OI (O'Connell and Marini 1999). In this study, the probands of Family 3 and Family 5 showed obvious dental malformations but were otherwise healthy in early childhood when they enrolled in the study. Both experienced subsequent bone fractures.

Children with OI may experience gross motor developmental delay, joint and ligament instability, muscle weakness, and lack of stamina (Rauch and Glorieux 2004). The delayed diagnosis precludes the possibility of early intervention to improve mobility, increase bone mass and muscle strength, protection to prevent facture cycles, and awareness for accommodations. Correct diagnosis and intervention may help OI patients to initiate treatment early to maximize growth potential of the bone, function, and strength (Engelbert et al. 1998). Physical therapy, early intervention, and physical activity potentially improve outcomes. It is therefore important in cases of autosomal dominant inherited dentin defects to determine the genetic etiology to discern if the dentin defects are isolated (mutations in *DSPP*) or part of a syndrome like OI.

As OI can be caused by defects in many genes besides type I collagen (Marini et al. 2014) and dentin malformations can be manifested in other inherited conditions besides OI, WES should be considered as the first‐line screening test, which sequences the *DSPP* coding region outside of the DPP repeat region as well as all potential OI‐causing genes for potential disease‐causing mutations. If no causality is identified, then SMRT sequencing should be used to sequence DPP. Aligning the SMRT sequence with those provided in Figure S2 identifies the DPP haplotype and the causative mutation. If no mutation is found, the WES data should be reanalyzed with an expanded list of candidate genes.

Including the two novel haplotypes characterized in our probands (F2b and F4a), there are 36 novel DPP length haplotypes, or patterns of 32 indels. Excluding the 16 indels that have been identified in only a single haplotype, there are 16 confirmed DPP indels that give rise to 25 novel *DSPP* length haplotypes (Fig. S3). The tree generated from the alignment of these haplotypes describes how the haplotypes might have evolved. Trees are generated by minimizing the number of genetic events needed to arrive at a solution. In this case, even the most efficient tree required some indels to be generated independently in different lines. This kind of result should be expected for large, highly repetitive sequences, as it is sometimes possible to align all indels of the same length (suggesting they were generated by a single event) even though they could have occurred at any site within a segment of DNA. Indel 15 (an 18‐bp deletion relative to the reference sequence) could have been generated by an 18‐bp deletion anywhere between Indel 14 and Indel 16 (Fig. 4). It is apparent from the tree that some indels were generated independently in different DPP haplotypes, presumably by strand slippage during DNA replication. As more DPP sequences are characterized, the accuracy of the tree will improve and potentially help resolve prehistorical human migration patterns (McKnight et al. 2008b). Sequence variations including indels in *DMP1* (a close relative of *DSPP*) were used to successfully deduce the phylogenetic relationship of 19 species of closely related bats (Van Den Bussche et al. 2003).

The most severe form of inherited dentin defects (Witkop et al. 1956) was first described in a group of early immigrants that became known as the “Brandywine isolate” (Witkop et al. 1966). The dentin malformations allowed for dental abscesses to form in primary teeth without decay or history of trauma and was designated as DGI‐III (Shields et al. 1973). “Compound mutations” in *DSPP* (indels that maintained the reading frame) were proposed to cause the DGI‐III in this group, but it was subsequently demonstrated that the dentin defects in Brandywine isolate were caused by a p.Pro17Ser missense alteration that segregated with the phenotype, caused endoplasmic reticulum retention of the protein in a functional assay (Hart and Hart 2007), and was disease‐causing in another DGI family (Zhang et al. 2007). No other studies have linked indels in *DSPP* that maintain the reading frame to inherited dentin malformations.

This report brings to 36 the number of novel *DSPP* mutations that cause inherited dentin defects. These mutations are transmitted in an autosomal dominant pattern and fall into two groups. Both cause the mutant protein to be retained in the rER and therefore fail to efficiently traffic out of the cell (von Marschall et al. 2012). There are 21 (3‐prime) defects within the DPP repetitive region in exon 5 (Table S5). All shift translation into the −1 reading frame and replace the downstream Asp/Ser‐rich sequence with one rich in Val, Ala, Ile, and Thr (McKnight et al. 2008b). The mutant DPP protein is actually longer (has more amino acids) than normal DPP no matter where the frameshift occurs in the DPP repeat region (Wang et al. 2011). The absence of any disease‐causing frameshift mutations into the −2 reading frame (which would truncate the DPP protein near the site of the frameshift in all cases) suggests that a loss of half of the normal amount of DPP at the protein level does not result in dentin defects. This is consistent with the observation that *Dspp* ^+/−^ mice lack a discernable dentin phenotype (Sreenath et al. 2003). However, while recombinant DSPP was expressed and secreted by HEK293 cells, secretion of this protein could be inhibited by coexpression of a recombinant DSPP with a −1 frameshift (von Marschall et al. 2012). It was proposed that capture of recombinant DSPP by the −1 frameshifted protein was mediated through the “formation of Ca^2+^‐dependent complexes” in the part of DPP that preceded the frameshift, which might explain why most of the 5′ DPP frameshifts exhibit the milder (DD‐II) phenotype (von Marschall et al. 2012). However, while the locations of the mutations that cause DD‐II or DGI‐II tend to cluster (Fig. S2), there is overlap (McKnight et al. 2008a), and *DSPP* mutations that cause DD‐II in one family or individual cause DGI‐II in others (Wang et al. 2011). It seems more probable that the formation of covalent dimers in the DSP domain via intermolecular disulfide bridging (Yamakoshi et al. 2005a) explains the ability of mutant DSPP to prevent the secretion of normal DSPP (haploinsufficiency). Cell pathology caused by the retention of the mutant protein in the rER (dominant negative effects) is also likely to be important (Wang et al. 2011). We suspect that with the application of SMRT sequencing more frameshift mutations in specific DPP haplotypes will be identified and provide a clearer picture of the relationship between the position of each mutation and the severity of the clinical phenotype.

## Conflict of Interest

The authors state that they have no conflicts of interest.

## Supporting information


**Figure S1.** Alignment of the *DSPP* 5′ region from reptiles and mammals with *DSPPL1* from Coelacanth.
**Figure S2.** Alignment of DPP coding region of *DSPP* showing allelic variants and disease‐causing mutations.
**Figure S3.** Patterns of 32 DPP indels in *DSPP* haplotypes.
**Figure S4.** Map showing the 25 different patterns (length haplotypes) of confirmed indels.

**Table S1.** Outcome analyses of whole‐exome sequencing.
**Table S2.** PCR primers and reaction conditions.
**Table S3A.** Thirty‐eight short *DSPP* haplotype sequences in NCBI PopSet: 162077127.
**Table S3B.** Twenty‐one longer *DSPP* haplotype sequences in NCBI PopSet: 162077085.
**Table S4.** List of insertions and deletions (indels) in the DPP repetitive region.
**Table S5.** Disease‐causing mutations in the coding region for DPP.
