# Supplementary material for: The dentin phosphoprotein repeat region and inherited defects of dentin
Source: Mol Genet Genomic Med. 2015 Sep 7;4(1):28–38. doi: 10.1002/mgg3.176 (PMC4707025; doi:10.1002/mgg3.176)
Supplement: Supplementary file 1 — Figure S1. Alignment of the DSPP 5′ region from reptiles and mammals with DSPPL1 from Coelacanth. Figure S2. Alignment of DPP coding region of DSPP showing allelic variants and disease‐causing mutations. Figure S3. Patterns of 32 DPP indels in DSPP haplotypes. Figure S4. Map showing the 25 different patterns (length haplotypes) of confirmed indels. [file MGG3-4-028-s001.docx]

**Fig. S1. Alignment of the *DSPP* 5’ region from reptiles and mammals with *DSPPL1* from Coelacanth.** Region in bold is the 5’ end of exon 1 in Human *DSPP*. This region in mouse was characterized by DNase I footprinting and other analyses that demonstrated the binding of multiple nuclear factors to this region (1).

***Key:*** Highly conserved (:), Moderately-well conserved (.) not conserved ( ).

**DSPP** Exon1

Human caagtgaaggaagtg-----gggaa--gttattatgtgcttcatagactcg----atga--cactttataa-cccca-tatcagggatcc-ta-aacagtgattggttg-agaaaattatcaaactgagtttaaatttcggcaagtacaaaattgtc**atgcaaa-agtc**

Chimp caagtgaaggaagtg-----gggaa--gttattatgtgcttcatagactcg----atga--cactttataa-cccca-tatcagggatcc-ta-aacagtgattggttg-agaaaattatcaaactgagtttaaatttcggcaagtacaaaattgtcatgcaaa-agtc

Gorilla caagtgaaggaagtg-----gggaa--gttattatgtgcttcatagactcg----atgc--cactttataa-cccca-tatcagggatcc-ta-aacagtgattggttg-agaaaattatcaaactgagtttaaatttcagcaagtacaaaattgtcatgcaaa-agtc

Orangutan caagtgaaggaagtg-----gggaa--gttattatgtgcttcatcgacacg----atga--cactttataa-cccca-tatcagggatcc-ta-aacagtgattggttg-agaaaattatcaaactgagtttaaatttcggcaagtacaaaattgtcatgcaaa-agtc

Gibbon caagtgaaggaagtg-----gggaa--gttattacgtgcttcatagacacg----atga--cactttataa-cccca-tatcagggatcc-ta-aacagtgattggttg-agaaaattatcaaactgagtttaaatttcggcaagtacaaaattgtcatgcaaa-agtc

Rhesus caagtgaaggaagtg-----gggaa--gttattatgtgcttcacagacacg----atga--cactttataa-cccca-tatcagggatcc-ta-aacagtgattggttg-agaaaattatcaaactgagtttaaatttcggcaagtacaaaattgtcatgcaaa-aatc

Crab-eating macaque caagtgaaggaagtg-----gggaa--gttattatgtgcttcatagacacg----atga--cactttataa-cccca-tatcagggatcc-ta-aacagtgattggttg-agaaaattatcaaactgagtttaaatttcggcaagtacaaaattgtcatgcaaa-aatc

Baboon caagtgaaggaagtg-----gggaa--gttattatgtgcttcatagacacg----atga--cactttataa-cccca-tatcagggatcc-ta-aacagtgattggttg-agaaaattatcaaactgagtttaaatttcggcaagtacaaaattgtcatgcaaa-aatc

Green monkey caagtgaaggaagtg-----gggaa--gttattatgtgcttcatagacacg----atga--cactttataa-cccca-tatcagggatcc-ta-aacagtgattggttg-agaaaattatcaaactgagtttaaatttcggcaactacaaaattgtcatgcaaa-aatc

Marmoset caagtgaaggaagtg-----gggaa--gttattatgtgcttcgtagacacg----atga--cactttataa-ccccg-tatcagggatcc-ta-agcagtgattggttg-agaaaattatcaaactcagtttaaatttcagcaggtacaaaattgtcatgcaaa-agtc

Squirrel monkey caagtgaaggaagtg-----gggaa--gttattatgtgcttcgtagacacg----atga--cactttataa-ccccg-tatcagggatcc-ta-aacagtgattggttg-agaaaattatcaaactgagtttaaatttcagcaggtacaaaattgtcatgcaaa-agtc

Bushbaby tgagttaaggaagta-----gggga--gttattatgtgcttcatagacctg----gtga--cactttataa-ccccc-tatcagcgatcc-ta-agcagtgattggttg-agaaaatgatcaaactgagtttaaatttcagcagatacaaaattgtcatgcaaa-agtc

Chinese tree shrew caagcgaaggaagtg-----gggat--gttattatgtgcttcgtacacgtg----gtga--cagttcataa-ccctg-catcaaggattc-ta-aacagtgattggttg-aggaaataatcaaactgagtttaaatttcagcaggtgcaaaattgtcatgcaaa-agtc

Squirrel c-catgaggaagt-------aggga--gttattatgtgcttcatagacaca----gtga--cactttataa-cccca-tatcagggatcc-ta-aacagtgattggttg-agaaaattatcaaactgagtttaaatttcggcaggtacaaaattgtcatgaaaa-actc

Lesser Egyptian jerboa tgagcaggggagt-------gggga--gttattatgagcttcataggcaca----gtga--cacttcataa-cccca-tatcagggatcc-tg-aacagtgattggttg-agaaaattatcaaactgagtttaaatttcgccaagtacaaaattgtcatgcagg-agca

Prairie vole cgagccagggagt-------ggggc--attattacgagc-tcgtaggccct----gtgacccactttaaac-cccca-catcagggatcctta-aacactgattggagg-agaaaattatcaaactgaatttaaacttcggcaggtacagaattgccatgcaaa-agtc

Chinese hamster cacgccagggagc-------ggggc--attattatgggcttcataggcaca----gtga--cactttaaac-cccca-caccagggatcc-ta-aacagtgattggtgg-a-aacattatcaaactgaatttaaatttcggcaggtacaaaattgtcatgtaaa-agct

Golden hamster cactccagggagc-------cgggc--attattatgggcttcataggcaca----gtga--cactttaaac-cccca-ctccagggatcc-ta-aacagtgattggtgg-a-aacatcatcaaattgaatttaaatttcggcaggtacaaaattgtcatgcaaa-agtc

Mouse cgagcgagggagt-------ggggc--gttattacagcc-tcataggcaca----ctga--ctctttaaac-cccca-catcagggatcc-ta-agcagtgattggttg-agaaaattatcaaactgaatttaaatttcagcaggtacaaaattgtcacgcaaaaagcc

Rat ggagcgagggagt-------ggggc--gttattacagat-tcagaggcact----gtga--ctttcaaaac-cccca-catcagggatcc-ta-aacagtgattggttg-agaaaattatcaaactgaatttaaatttcagcaggtacaaaattgtcacgcaaa-agcc

Naked mole-rat catgtgaggaagt-------ggggc--gttattatgagct-cacagacaca----ggga--cactttataa-cccca-tatcagggatcc-ga-aacagtgattggctg-agaaaattatcaaaccgagtttaaatttcagcaggtagaaaattgtcatgcaaa-agtc

Guinea pig caggcgaggaagt-------ggggc--gttattacgagctccacagacaca----ggga--caccttgcaa-cccca-tatcagggatcc-ga-aacagtgattggcta-agaaaattatcaaaccgagcttaaatttcgtcaggtagaaaattgtcatgcaaa-agtc

Chinchilla caagtgagggagt-------ggggc--attattacaagctttgcagacaca----ggga--caatttataa-cccca-tatcagggatcc-aa-aactgtgattggctg-agaaaatgatcaaaccgagcttaaatttcctcaggtaggaaactgtcatgcaaa-agtc

Brush-tailed rat caagtgaggaagt-------ggggc--gttattatgagcttcacagacaca----ggga--caccgtataa-cccca-catcagggatcc-aa-aacagcgattggctg-agaaaattatcaaactgagtttaaatttcgtctggtagaaaagcatcatgcaaa-aatc

Rabbit caagagaaggaag-------tggga--attattatgggcttcataggcaca----gtga--cactttataa-cccca-tatcagggatcc-ta-aacagcgattggttg-acaaaattatcaaactgagtttaaatttgggcaggtacaaaattgtcatgcaaa-actc

Pika caagagaaggaag-------tggga--attattatgggcttcataggtaca----gtga--cactttataa-cccca-tatcagggatcc-aa-aacagtgattggttg-acaaaactatcaaactgagtttaaatttcggcaggtacaaaaatggtatgagaa-actc

Pig caagtgaaggaagtg-----ggggac-gttattatgtgcttcatagacacg----gtga--cactttataa-cccca-tatcaaggatcc-ta-aacagtgattggttg-agaaaattatcaaactgactttaaatttcggcaggtacaaaattgtcacgcaaa-agtc

Alpaca caagtgaaggaagtg-----ggggac-gttattatgtgcttcatagacatg----gtga--cactttataa-cccca-tatcagggatcc-ta-aacagtgattggttg-agaaaattatcaaactgactttaaatttcggcaggtacaaaattgccatgcaaa-agtc

Bactrian camel caagtgaaggaagtg-----ggggac-tttattatgtgcttcatagacacg----gtga--cactttataa-cccca-tatcagggatcc-ta-aacagtgattggttg-agaaaattatcaaactgactttaaatttcggcaggtataaaattgtcatgcaaa-agtc

Dolphin caagtgaaggaagtg-----gggtaa-cttattatgtgcttcatagacaca----gtga--cactttataa-cccca-tatcaaggatcc-ta-aacagtgattggttg-agaaaattatcaaactgactttaaatttcggcaggtacaaaattgtcatgcaaa-agcc

Killer whale caagtgaaggaagtg-----gggtaa-cttattatgtgcttcatagacaca----ctga--cactttataa-ccccg-tatcaaggatcc-ta-aacagtgattggttg-agaaaattatcaaactgactttaaatttcggcaggtacaaaattgtcatgcaaa-agcc

Tibetan antelope caagtgaaggaagtg-----ggggaa-gttattatgtgcttcatagacatg----gtga--cactttataa-ccctg-tatcaaggatcc--a-aacagtgattggttg-agataattatcaaactgactttaaatttcggcaggaacaaaattgtcctgcaaa-agtc

Cow caggtgaaggaagtg-----ggggaa-gttattatgtgcctcatagacatg----gtga--cactttataa-ccctg-tatcaaggatcc--a-aacagtgattggttg-agataattatcaaactgactttaaatttcagcaggaacaaaattgtcatgcaaa-agtc

Sheep caagtgaaggaagtg-----ggggaa-gttattatgtgcttcatagacatg----gtga--cactttataa-ccctg-tatcaaggatcc--a-aacagtgattggttg-agataattatcaaactgactttaaatttcggcaggaacaaaattgtcatgcaaa-agtc

Domestic goat caagtgaaggaagtg-----ggggaa-gttattatgtgcttcatagacatg----gtga--cactttataa-ccctg-tatcaaggatcc--a-aacagtgattggttg-agataattatcaaactgactttaaatttcggcaggaacaaaattgtcatgcaaa-agtc

Horse caagtgaaggaagtg-----ggggac-gttattatgtgcttcatagacacg----gcga--cactttataa-cccca-catcagggatcc-ta-aacagtgattggttg-agaaaattatcaaactgagtttaaatttcggcaggtacaaaattgtcatgcaaa-agtc

White rhinoceros caagtgaaggaagtg-----gggaa--gttattatgtgcttcatagacacg----gtga--cactttataa-cccca-tatcagggatcc-ta-aacagtgattggttg-agaaaattatcaaactgagtttaaatttcggcaggtacaaaattgtcatgcaaa-agtc

Cat tatgtgaaggaagtg-----ggggaa-gttattatgtgcttcatagacacg----gtga--cactttataa-cccca-tatcagggatcc-ta-aacagtgattggctg-agaaaattatcaaactgagtttaaatttcagcaggtacaaaattgtcacgcaaa-agtc

Dog taagtgaaggaagtg-----ggggaa-gttattatgtgcttcatagacatg----gtga--cactttataa-cccta-tatcagggatcc-ta-aacagtgattggttg-agaaaattatcaaactgagtttaaatttcagcaggtacaaaactgtcacccaaa-agtc

Ferret tatgtgaaggaagtg-----ggggaa-gttattatgtgcttcatagacatg----gtga--cactttataa-cccca-tatcagggatcc-ta-aacagtgattggttg-agaaaattatcaaactgagtttaaatttcagcaggtacaaaattgtcacgtaaa-tgtc

Panda tatgtgaaggaagtg-----ggggaa-gttattatgtgcttcatagacatg----gtga--cactttataa-cccca-tatca-ggatcc-ta-aacagtgattggttg-agaaaattatcaaactgagtttaaatttcagcaggtacaaaatcgtcatgcaaa-agtc

Pacific walrus tatgtgaaggaagta-----ggggaa-gttattatgtacttcatagacatg----gtga--cactttataa-cccca-tatcagggatcc-ta-aacagtgattggttg-agaaaattatcaaactgagtttaaatttcagcaggtacaaaattgtcatgcaaa-agtc

Weddell seal tatgtgaaggaagtg-----ggggaa-gttactatgtgcttcatagacatg----gtga--cactttataa-ccccattatcagggatcc-ta-aacagtgattggttg-agaaaatgatcaaactgagtttaaatttcagcaggtacaaaattgtcatgcaaa-agtc

Black flying-fox caagtgaaggaagta-----gaggaa-gttattatgtgcttcgtagacaca----gtaa--cactttagaa-cctta-tatcagggatcc-ta-aaaagtgattggttg-agaaaattatcagactgagtttaaatttcggcaggtagaaaattgtcatgcaaa-agtc

Megabat caagtgaaggaagta-----gaggaa-gttattatgtgcttcgtagacaca----gtga--cactttagaa-cctta-tatcagggatcc-ta-aaaagtgattggttg-agaaaattatcagactgagtttaaatttcggcaggtagaaaattgtcatgcaaa-agtc

David's myotis (bat) caagtggagggagag-----gaggaa-gttattatgtgcttcacagacacggaccgtga--cacgctataa-cccta-tatcagggatcc-ta-aacagtgattggtgg-agaaaattatcaaactgagtttaaatttcggcaggtacgaaattatcatgcaaa-agtc

Microbat caagtggagggagag-----gaggaa-gttattatgtgcttcacagacacg----gtga--cacgctataa-cccta-tatcagggattc-ta-aacagtgattggtgg-agaaaattatcaaactgagtttaaatttcggcaggtacgaaattatcatgcaaa-agtc

Big brown bat caagtggagggagag-----gaggaa-gttattatgtgtttcacagacacg----gtga--catgctataa-cccta-tatcagggatcc-ta-aacagtgattggtgg-agaaaattatcaaactgagtttaaatttcggcaggtacgaaattatcatgcaaa-agtc

Hedgehog ccagtgaaaaaagtggaggaggggga-gttattatgtgcttcaggggctta----gtga--cactttataa-cccag-tatcagggattc-ta-agcagtgattggttg-agaaaatgatcaaactgagtttaaatttcagcaggtgcaaaattgtcactcaa------

Star-nosed mole caagtgaaggaagtg-----agggaa-gttattatgtgcttcatagacaca----ggga--cactttataa-cccca-tatcagggatcc-ga-aacagtgattggttg-agaaaattatcaaactgagtttaaatttgggcaggtacaaaattgtcatgcaaa-agtc

Elephant cgagtgaaggaagtg-----ggggaa-gttattatatgcttcatagacatg----gaga--tattttgtaa-ccccc-tatcagggttcc-ta-aacagtgattggttg-agaaaattatcaaa-tcagtttaaatttcggcaggtacaaaattgtcatgcaaa-agtc

Cape elephant shrew caagtgaaggaagtg-----ggggaa-gttattatgtggctcatagacaca----gcaa--cactttgtaacccccc-tatcagagatcc-ta-aacagcgattggttg-agaaaatgatcaaa-tgagtttaaatttcggcaggtacaaaattgtcatgtaag-agtc

Manatee caagtgaaggaagtg-----ggggaa-gttattatgttcttcatagacacg----gtga--cattttgtaa-ccccc-tatcagggttcc-ta-aacagtgattggttg-agaaaattatcaaa-tgagtttaaatttcagcaggtagaaaattgtcatgcaaa-agtc

Cape golden mole caagtgaaggaagtg-----ggggaa-gttattatgtgcttcatagacata----gtga--cactttgtaa-ccccc-tatcagggatcc-ta-aacactgattggttgagaaaaattatcaaa-tgagtttaaatttccgcagatacaaaattgtcatacacg-aatc

Tenrec caaatgaaggaagtg-----gggggacattattatgtgcttcatagacacg----gtga--cactttgtaa-cccac-tatcagggatcc-ta-aacagtgattggttg-ggaaaattatcaaa-tgactttaaatttcggcaggtacaaaattgtcacgcaag-agtc

Aardvark caagtgaaggaagtg-----ggggaa-attattatgtgttgcacagacaca----ggga--cactttgtaa-ccccc-tatcagggatcc-ta-aacagtgattggttg-agaaaattatcaaa-tgagtttaaatttcagcaggtacaaaattttcatgcaag-agtc

Armadillo caagtgaaggaagta-----agggaa-gctattatgtgcctcaaagacaca----gtga--cactttataa-cccca-tatcacggatcc-ta-tacagtgattggttg-agaaaattatcaaactgagtttaaatttcagcaggtagaaaattatcaaactga-gttt

Opossum tatgt---------------gggaa--gttaatatgagcttcctggtccca----gcca--cacctcacaa-cccca-tatcagggcttc-ca-gatgatgattggtgg-atataattttgaaacggagtttaaatttcagtagctacacaagagtcatacaaa-aata

Tasmanian devil tatgt---------------gggaa--gttaatatgagcttcatggtcaca----gcca--cacctcacaa-cccca-tatcaggatttc-ca-gatggtgattggtgg-acataattgtcaaacagagtttaaatttcagtagctacataaaagtcaaacaaa-aata

Platypus cccctgagggatgtg-----aagaa--gttaatatgtgcttcaacgtcaaa----gtta--caccttacaa-cctca-tatcagagtcca-ca-agcactgactggttg-agataattgggaagcagagtttaaatttcagcaggtacaaaagtgttatgccaa-acta

American alligator cctgtgaaggaagtg-----tgggag-gttaatatgtgctccacaatcaaaacttacac--cttcttgcat-cc----tatcagggtctt-ca-agtcatgattggttg-atatatttgtg-agcagagtttaaattccagcaggtactaaagtgatgcacaaa-aagc

Lizard tctgttaagaaaacg-----tgggtg-gttaatatgtacttcaccaccaaa----gaga--cgtcttgca------------agtacccc-aagagcaatgattggtta-gtattttactgaagcagagtttaaatttcagcaagtccaaaggtggcaagcaaagcgcc

**DSPPL1 : .::: .: ::: : .:::.:: :: :: .... :: . ::: : . :.: .:::::::::. . .:::::::::::::::::... ::. .: : :** :::: : .

Coelacanth ca----ataaaattg-----tggtag-attagtaggttct-agttgacacc----ttgt--ga--ttgtga-catca-tacctg---tta-tg-aactgtgattggttg-tttagaatgtgaagcagagtttaaatccctgcagt----gaattctctagcacagaagc

**Reference:** Chen S, Unterbrink A, Kadapakkam S, Dong J, Gu TT, Dickson J *et al.* (2004). Regulation of the Cell Type-specific dentin sialophosphoprotein gene expression in mouse odontoblasts by a novel transcription repressor and an activator CCAAT-binding factor. *J Biol Chem* 279(40)*:*42182-42191.

**Fig. S2. Alignment of DPP coding region of *DSPP* showing allelic variants and disease-causing mutations (pages 1-14).** Ten DPP sequences (F1A through F5B) from 5 patients with inherited dentin defects are shown aligned to all previously characterized DPP sequences exhibiting novel patterns of insertions and deletions (indels). Previously characterized DPP haplotypes were downloaded from NCBI PopSets 162077127 and 162077085 (2). These PopSets contain DPP haplotype sequences from 2 different sizes of cloned DPP polymerase chain reaction products (3) and are labeled “HAP#”. Haplotypes labeled “SHAP#" were characterized in China and published, but were not submitted to GenBank (4). However, the locations of all indels were clearly described with respect to the reference sequence. The alignments were made manually, trying to minimize the number of genetic events that would have been required to generate the observed allelic differences in DPP sequences. Only a single previously published DPP sequences for each indel pattern was retained in the final alignment. “REFSEQ” is the NCBI *DSPP* reference sequence (NM_014208.3). “MCKNIGHT” is the previously published mutated DSPP sequence of our proband from family 1 (sequence F1A) with type II dentin dysplasia (3). “F1A” through “F5B” are the 10 DPP allele sequences determined by SMRT sequencing of DPP amplification products from the probands of our 5 families. “HAP1A to HAP38A” are the longer DPP haplotype sequences, while HAP13-HAP35 are shorter DPP sequences previously submitted to GenBank (2). “MERGED” is a hypothetical *DSPP* haplotype that contains the sequences of all indels in the alignment, while “TRANSL” is the translation of the “MERGED” sequence. Notes provide the locations and phenotypes of mutations associated with inherited dentin defects. Blue highlight indicates a mutation that was manifested as type II dentin dysplasia. Green highlight indicates a mutation that was manifested as dentinogenesis imperfecta. The nomenclature used for all disease-causing *DSPP* sequence variations were verified using *Mutalyzer* 2.0.3 at http://www.lovd.nl/mutalyzer/. Nucleotides (polymorphisms) that vary from the reference sequences are in bold. Numbers identify nucleotides or amino acids in the *DSPP* cDNA reference sequence. A haplotype is said to have an indel when it differs in length from the REFSEQ. There are 100 nucleotides in each row to facilitate the alignment of new sequences. We found viewing this data best at 250% scale.

1572

RefSeq (1387) gatgatcccaatagcagtgatgaatctaatggcaatgatgatgctaattcagaaagtgacaataacagcagtagccgaggagatgcttcttataactctgatgaatcaaaagataatggcaatggcagtgactcaaaaggagcagaagatgatgacagtgatagcacatcagacactaataatagt

McKnight atgcaaggagatgatcccaatagcagtgatgaatctaatggcaatgatgatgctaattcagaaagtgacaataacagcagtagccgaggagatgcttcttataactctgatgaatcaaaagataatggcaatggcagtgactcaaaaggagcagaagatgatgacagtgatagcacatcagacactaataatagt

F1a AGTCCATGCAAGGAGATGATCCCAATAGCAGTGATGAATCTAATGGCAATGATGATGCTAATTCAGAAAGTGACAATAACAGCAGTAGCCGAGGAGATGCTTCTTATAACTCTGATGAATCAAAAGATAATGGCAATGGCAGTGACTCAAAAGGAGCAGAAGATGATGACAGTGATAGCACATCAGACACTAATAATAGT

F1b AGTCCATGCAAGGAGATGATCCCAATAGCAGTGATGAATCTAATGGCAATGATGATGCTAATTCAGAAAGTGACAATAACAGCAGTAGCCGAGGAGATGCTTCTTATAACTCTGATGAATCAAAAGATAATGGCAATGGCAGTGACTCAAAAGGAGCAGAAGATGATGACAGTGATAGCACATCAGACACTAATAATAGT

F2a AGTCCATGCAAGGAGATGATCCCAATAGCAGTGATGAATCTAATGGCAATGATGATGCTAATTCAGAAAGTGACAATAACAGCAGTAGCCGAGGAGATGCTTCTTATAACTCTGATGAATCAAAAGATAATGGCAATGGCAGTGACTCAAAAGGAGCAGAAGATGATGACAGTGATAGCACATCAGACACTAATAATAGT

F2b AGTCCATGCAAGGAGATGATCCCAATAGCAGTGATGAATCTAATGGCAATGATGATGCTAATTCAGAAAGTGACAATAACAGCAGTAGCCGAGGAGATGCTTCTTATAACTCTGATGAATCAAAAGATAATGGCAATGGCAGTGACTCAAAAGGAGCAGAAGATGATGACAGTGATAGCACATCAGACACTAATAATAGT

F3a AGTCCATGCAAGGAGATGATCCCAATAGCAGTGATGAATCTAATGGCAATGATGATGCTAATTCAGAAAGTGACAATAACAGCAGTAGCCGAGGAGATGCTTCTTATAACTCTGATGAATCAAAAGATAATGGCAATGGCAGTGACTCAAAAGGAGCAGAAGATGATGACAGTGATAGCACATCAGACACTAATAATAGT

F3b AGTCCATGCAAGGAGATGATCCCAATAGCAGTGATGAATCTAATGGCAATGATGATGCTAATTCAGAAAGTGACAATAACAGCAGTAGCCGAGGAGATGCTTCTTATAACTCTGATGAATCAAAAGATAATGGCAATGGCAGTGACTCAAAAGGAGCAGAAGATGATGACAGTGATAGCACATCAGACACTAATAATAGT

F4a AGTCCATGCAAGGAGATGATCCCAATAGCAGTGATGAATCTAATGGCAATGATGATGCTAATTCAGAAAGTGACAATAACAGCAGTAGCCGAGGAGATGCTTCTTATAACTCTGATGAATCAAAAGATAATGGCAATGGCAGTGACTCAAAAGGAGCAGAAGATGATGACAGTGATAGCACATCAGACACTAATAATAGT

F4b AGTCCATGCAAGGAGATGATCCCAATAGCAGTGATGAATCTAATGGCAATGATGATGCTAATTCAGAAAGTGACAATAACAGCAGTAGCCGAGGAGATGCTTCTTATAACTCTGATGAATCAAAAGATAATGGCAATGGCAGTGACTCAAAAGGAGCAGAAGATGATGACAGTGATAGCACATCAGACACTAATAATAGT

F5a AGTCCATGCAAGGAGATGATCCCAATAGCAGTGATGAATCTAATGGCAATGATGATGCTAATTCAGAAAGTGACAATAACAGCAGTAGCCGAGGAGATGCTTCTTATAACTCTGATGAATCAAAAGATAATGGCAATGGCAGTGACTCAAAAGGAGCAGAAGATGATGACAGTGATAGCACATCAGACACTAATAATAGT

F5b AGTCCATGCAAGGAGATGATCCCAATAGCAGTGATGAATCTAATGGCAATGATGATGCTAATTCAGAAAGTGACAATAACAGCAGTAGCCGAGGAGATGCTTCTTATAACTCTGATGAATCAAAAGATAATGGCAATGGCAGTGACTCAAAAGGAGCAGAAGATGATGACAGTGATAGCACATCAGACACTAATAATAGT

Hap1a CGAGGAGATGCTTCTTATAACTCTGATGAATCAAAAGATAATGGCAATGGCAGTGACTCAAAAGGAGCAGAAGATGATGACAGTGATAGCACATCAGACACTAATAATAGT

Hap2a CGAGGAGATGCTTCTTATAACTCTGATGAATCAAAAGATAATGGCAATGGCAGTGACTCAAAAGGAGCAGAAGATGATGACAGTGATAGCACATCAGACACTAATAATAGT

Hap3a CGAGGAGATGCTTCTTATAACTCTGATGAATCAAAAGATAATGGCAATGGCAGTGACTCAAAAGGAGCAGAAGATGATGACAGTGATAGCACATCAGACACTAATAATAGT

Hap15a CGAGGAGATGCTTCTTATAACTCTGATGAATCAAAAGATAATGGCAATGGCAGTGACTCAAAAGGAGCAGAAGATGATGACAGTGATAGCACATCAGACACTAATAATAGT

Hap17b cgaggagatgcttcttataactctgatgaatcaaaagataatggcaatggcagtgactcaaaaggagcagaagatgatgacagtgatagcacatcagacactaataatagt

Hap20a CGAGGAGATGCTTCTTATAACTCTGATGAATCAAAAGATAATGGCAATGGCAGTGACTCAAAAGGAGCAGAAGATGATGACAGTGATAGCACATCAGACACTAATAATAGT

Hap20b cgaggagatgcttcttataactctgatgaatcaaaagataatggcaatggcagtgactcaaaaggagcagaagatgatgacagtgatagcacatcagacactaataatagt

Hap36a CGAGGAGATGCTTCTTATAACTCTGATGAATCAAAAGATAATGGCAATGGCAGTGACTCAAAAGGAGCAGAAGATGATGACAGTGATAGCACATCAGACACTAATAATAGT

Hap37a CGAGGAGATGCTTCTTATAACTCTGATGAATCAAAAGATAATGGCAATGGCAGTGACTCAAAAGGAGCAGAAGATGATGACAGTGATAGCACATCAGACACTAATAATAGT

Hap38a CGAGGAGATGCTTCTTATAACTCTGATGAATCAAAAGATAATGGCAATGGCAGTGACTCAAAAGGAGCAGAAGATGATGACAGTGATAGCACATCAGACACTAATAATAGT

SHap1: gatgatcccaatagcagtgatgaatctaatggcaatgatgatgctaattcagaaagtgacaataacagcagtagccgaggagatgcttcttataactctgatgaatcaaaagataatggcaatggcagtgactcaaaaggagcagaagatgatgacagtgatagcacatcagacactaataatagt

SHap2: gatgatcccaatagcagtgatgaatctaatggcaatgatgatgctaattcagaaagtgacaataacagcagtagccgaggagatgcttcttataactctgatgaatcaaaagataatggcaatggcagtgactcaaaaggagcagaagatgatgacagtgatagcacatcagacactaataatagt

SHap3: gatgatcccaatagcagtgatgaatctaatggcaatgatgatgctaattcagaaagtgacaataacagcagtagccgaggagatgcttcttataactctgatgaatcaaaagataatggcaatggcagtgactcaaaaggagcagaagatgatgacagtgatagcacatcagacactaataatagt

SHap4: gatgatcccaatagcagtgatgaatctaatggcaatgatgatgctaattcagaaagtgacaataacagcagtagccgaggagatgcttcttataactctgatgaatcaaaagataatggcaatggcagtgactcaaaaggagcagaagatgatgacagtgatagcacatcagacactaataatagt

SHap5: gatgatcccaatagcagtgatgaatctaatggcaatgatgatgctaattcagaaagtgacaataacagcagtagccgaggagatgcttcttataactctgatgaatcaaaagataatggcaatggcagtgactcaaaaggagcagaagatgatgacagtgatagcacatcagacactaataatagt

SHap6: gatgatcccaatagcagtgatgaatctaatggcaatgatgatgctaattcagaaagtgacaataacagcagtagccgaggagatgcttcttataactctgatgaatcaaaagataatggcaatggcagtgactcaaaaggagcagaagatgatgacagtgatagcacatcagacactaataatagt

SHap6(2) gatgatcccaatagcagtgatgaatctaatggcaatgatgatgctaattcagaaagtgacaataacagcagtagccgaggagatgcttcttataactctgatgaatcaaaagataatggcaatggcagtgactcaaaaggagcagaagatgatgacagtgatagcacatcagacactaataatagt

SHap7 gatgatcccaatagcagtgatgaatctaatggcaatgatgatgctaattcagaaagtgacaataacagcagtagccgaggagatgcttcttataactctgatgaatcaaaagataatggcaatggcagtgactcaaaaggagcagaagatgatgacagtgatagcacatcagacactaataatagt

SHap102 gatgatcccaatagcagtgatgaatctaatggcaatgatgatgctaattcagaaagtgacaataacagcagtagccgaggagatgcttcttataactctgatgaatcaaaagataatggcaatggcagtgactcaaaaggagcagaagatgatgacagtgatagcacatcagacactaataatagt

SHap130 gatgatcccaatagcagtgatgaatctaatggcaatgatgatgctaattcagaaagtgacaataacagcagtagccgaggagatgcttcttataactctgatgaatcaaaagataatggcaatggcagtgactcaaaaggagcagaagatgatgacagtgatagcacatcagacactaataatagt

SHap106 gatgatcccaatagcagtgatgaatctaatggcaatgatgatgctaattcagaaagtgacaataacagcagtagccgaggagatgcttcttataactctgatgaatcaaaagataatggcaatggcagtgactcaaaaggagcagaagatgatgacagtgatagcacatcagacactaataatagt

SHap72 gatgatcccaatagcagtgatgaatctaatggcaatgatgatgctaattcagaaagtgacaataacagcagtagccgaggagatgcttcttataactctgatgaatcaaaagataatggcaatggcagtgactcaaaaggagcagaagatgatgacagtgatagcacatcagacactaataatagt

SHap110 gatgatcccaatagcagtgatgaatctaatggcaatgatgatgctaattcagaaagtgacaataacagcagtagccgaggagatgcttcttataactctgatgaatcaaaagataatggcaatggcagtgactcaaaaggagcagaagatgatgacagtgatagcacatcagacactaataatagt

Merged gatgatcccaatagcagtgatgaatctaatggcaatgatgatgctaattcagaaagtgacaataacagcagtagccgaggagatgcttcttataactctgatgaatcaaaagataatggcaatggcagtgactcaaaaggagcagaagatgatgacagtgatagcacatcagacactaataatagt

Transl (463) D D P N S S D E S N G N D D A N S E S D N N S S S R G D A S Y N S D E S K D N G N G S D S K G A E D D D S D S T S D T N N S 524

1573 1686 1772

RefSeq gacagtaatggcaatggtaacaatgggaatgatgacaatgacaaatcagacagtggcaaaggtaaatcagatagcagtgacagtgatagtagtgatagcagcaatagcagtga**t**agtagtgacagcagtgacagtgacagcagtgatagcaacagtagcagtgatagtgacagcagtgacagtgacagcagtgatagcag

McKnight gacagtaatggcaatggtaacaatgggaatgatgacaatgacaaatcagacagtggcaaaggtaaatcagatagcagtgacagtgatagtagtgatagcagcaatagcagtgatagtagtgacagcagtgacagtgacagcagtgatagcaacagtagcagtgatagtgacagcagtgacagtgacagcagtgatagcag

F1a GACAGTAATGGCAATGGTAACAATGGGAATGATGACAATGACAAATCAGACAGTGGCAAAGGTAAATCAGATAGCAGTGACAGTGATAGTAGTGATAGCAGCAATAGCAGTGATAGTAGTGACAGCAGTGACAGTGACAGCAGTGATAGCAACAGTAGCAGTGATAGTGACAGCAGTGACAGTGACAGCAGTGATAGCAG

F1b GACAGTAATGGCAATGGTAACAATGGGAATGATGACAATGACAAATCAGACAGTGGCAAAGGTAAATCAGATAGCAGTGACAGTGATAGTAGTGATAGCAGCAATAGCAGTGATAGTAGTGACAGCAGTGACAGTGACAGCAGTGATAGCAACAGTAGCAGTGATAGTGACAGCAGTGACAGTGACAGCAGTGATAGCAG

F2a GACAGTAATGGCAATGGTAACAATGGGAATGATGACAATGACAAATCAGACAGTGGCAAAGGTAAATCAGATAGCAGTGACAGTGATAGTAGTGATAGCAGCAATAGCAGTGATAGTAGTGACAGCAGTGACAGTGACAGCAGTGATAGCAACAGTAGCAGTGATAGTGACAGCAGTGACAGTGACAGCAGTGATAGCAG

F2b GACAGTAATGGCAATGGTAACAATGGGAATGATGACAATGACAAATCAGACAGTGGCAAAGGTAAATCAGATAGCAGTGACAGTGATAGTAGTGATAGCAGCAATAGCAGTGATAGTAGTGACAGCAGTGACAGTGACAGCAGTGATAGCAACAGTAGCAGTGATAGTGACAGCAGTGACAGTGACAGCAGTGATAGCAG

F3a GACAGTAATGGCAATGGTAACAATGGGAATGATGACAATGACAAATCAGACAGTGGCAAAGGTAAATCAGATAGCAGTGACAGTGATAGTAGTGATAGCAGCAATAGCAGTGATAGTAGTGACAGCAGTGACAGTGACAGCAGTGATAGCAACAGTAGCAGTGATAGTGACAGCAGTGACAGTGACAGCAGTGATAGCAG

F3b GACAGTAATGGCAATGGTAACAATGGGAATGATGACAATGACAAATCAGACAGTGGCAAAGGTAAATCAGATAGCAGTGACAGTGATAGTAGTGATAGCAGCAATAGCAGTGATAGTAGTGACAGCAGTGACAGTGACAGCAGTGATAGCAACAGTAGCAGTGATAGTGACAGCAGTGACAGTGACAGCAGTGATAGCAG

F4a GACAGTAATGGCAATGGTAACAATGGGAATGATGACAATGACAAATCAGACAGTGGCAAAGGTAAATCAGATAGCAGTGACAGTGATAGTAGTGATAGCAGCAATAGCAGTGATAGTAGTGACAGCAGTGACAGTGACAGCAGTGATAGCAACAGTAGCAGTGATAGTGACAGCAGTGACAGTGACAGCAGTGATAGCAG

F4b GACAGTAATGGCAATGGTAACAATGGGAATGATGACAATGACAAATCAGACAGTGGCAAAGGTAAATCAGATAGCAGTGACAGTGATAGTAGTGATAGCAGCAATAGCAGTGATAGTAGTGACAGCAGTGACAGTGACAGCAGTGATAGCAACAGTAGCAGTGATAGTGACAGCAGTGACAGTGACAGCAGTGATAGCAG

F5a GACAGTAATGGCAATGGTAACAATGGGAATGATGACAATGACAAATCAGACAGTGGCAAAGGTAAATCAGATAGCAGTGACAGTGATAGTAGTGATAGCAGCAATAGCAGTGATAGTAGTGACAGCAGTGACAGTGACAGCAGTGATAGCAACAGTAGCAGTGATAGTGACAGCAGTGACAGTGACAGCAGTGATAGCAG

F5b GACAGTAATGGCAATGGTAACAATGGGAATGATGACAATGACAAATCAGACAGTGGCAAAGGTAAATCAGATAGCAGTGACAGTGATAGTAGTGATAGCAGCAATAGCAGTGATAGTAGTGACAGCAGTGACAGTGACAGCAGTGATAGCAACAGTAGCAGTGATAGTGACAGCAGTGACAGTGACAGCAGTGATAGCAG

Hap1a GACAGTAATGGCAATGGTAACAATGGGAATGATGACAATGACAAATCAGACAGTGGCAAAGGTAAATCAGATAGCAGTGACAGTGATAGTAGTGATAGCAGCAATAGCAGTGATAGTAGTGACAGCAGTGACAGTGACAGCAGTGATAGCAACAGTAGCAGTGATAGTGACAGCAGTGACAGTGACAGCAGTGATAGCAG

Hap2a GACAGTAATGGCAATGGTAACAATGGGAATGATGACAATGACAAATCAGACAGTGGCAAAGGTAAATCAGATAGCAGTGACAGTGATAGTAGTGATAGCAGCAATAGCAGTGATAGTAGTGACAGCAGTGACAGTGACAGCAGTGATAGCAACAGTAGCAGTGATAGTGACAGCAGTGACAGTGACAGCAGTGATAGCAG

Hap3a GACAGTAATGGCAATGGTAACAATGGGAATGATGACAATGACAAATCAGACAGTGGCAAAGGTAAATCAGATAGCAGTGACAGTGATAGTAGTGATAGCAGCAATAGCAGTGATAGTAGTGACAGCAGTGACAGTGACAGCAGTGATAGCAACAGTAGCAGTGATAGTGACAGCAGTGACAGTGACAGCAGTGATAGCAG

Hap15a GACAGTAATGGCAATGGTAACAATGGGAATGATGACAATGACAAATCAGACAGTGGCAAAGGTAAATCAGATAGCAGTGACAGTGATAGTAGTGATAGCAGCAATAGCAGTGATAGTAGTGACAGCAGTGACAGTGACAGCAGTGATAGCAACAGTAGCAGTGATAGTGACAGCAGTGACAGTGACAGCAGTGATAGCAG

Hap17b gacagtaatggcaatggtaacaatgggaatgatgacaatgacaaatcagacagtggcaaaggtaaatcagatagcagtgacagtgatagtagtgatagcagcaatagcagtgatagtagtgacagcagtgacagtgacagcagtgatagcaacagtagcagtgatagtgacagcagtgacagtgacagcagtgatagcag

Hap20a GACAGTAATGGCAATGGTAACAATGGGAATGATGACAATGACAAATCAGACAGTGGCAAAGGTAAATCAGATAGCAGTGACAGTGATAGTAGTGATAGCAGCAATAGCAGTGATAGTAGTGACAGCAGTGACAGTGACAGCAGTGATAGCAACAGTAGCAGTGATAGTGACAGCAGTGACAGTGACAGCAGTGATAGCAG

Hap20b gacagtaatggcaatggtaacaatgggaatgatgacaatgacaaatcagacagtggcaaaggtaaatcagatagcagtgacagtgatagtagtgatagcagcaatagcagtgatagtagtgacagcagtgacagtgacagcagtgatagcaacagtagcagtgatagtgacagcagtgacagtgacagcagtgatagcag

Hap36a GACAGTAATGGCAATGGTAACAATGGGAATGATGACAATGACAAATCAGACAGTGGCAAAGGTAAATCAGATAGCAGTGACAGTGATAGTAGTGATAGCAGCAATAGCAGTGATAGTAGTGACAGCAGTGACAGTGACAGCAGTGATAGCAACAGTAGCAGTGATAGTGACAGCAGTGACAGTGACAGCAGTGATAGCAG

Hap37a GACAGTAATGGCAATGGTAACAATGGGAATGATGACAATGACAAATCAGACAGTGGCAAAGGTAAATCAGATAGCAGTGACAGTGATAGTAGTGATAGCAGCAATAGCAGTGATAGTAGTGACAGCAGTGACAGTGACAGCAGTGATAGCAACAGTAGCAGTGATAGTGACAGCAGTGACAGTGACAGCAGTGATAGCAG

Hap38a GACAGTAATGGCAATGGTAACAATGGGAATGATGACAATGACAAATCAGACAGTGGCAAAGGTAAATCAGATAGCAGTGACAGTGATAGTAGTGATAGCAGCAATAGCAGTGATAGTAGTGACAGCAGTGACAGTGACAGCAGTGATAGCAACAGTAGCAGTGATAGTGACAGCAGTGACAGTGACAGCAGTGATAGCAG

SHap1: gacagtaatggcaatggtaacaatgggaatgatgacaatgacaaatcagacagtggcaaaggtaaatcagatagcagtgacagtgatagtagtgatagcagcaatagcagtgatagtagtgacagcagtgacagtgacagcagtgatagcaacagtagcagtgatagtgacagcagtgacagtgacagcagtgatagcag

SHap2: gacagtaatggcaatggtaacaatgggaatgatgacaatgacaaatcagacagtggcaaaggtaaatcagatagcagtgacagtgatagtagtgatagcagcaatagcagtgatagtagtgacagcagtgacagtgacagcagtgatagcaacagtagcagtgatagtgacagcagtgacagtgacagcagtgatagcag

SHap3: gacagtaatggcaatggtaacaatgggaatgatgacaatgacaaatcagacagtggcaaaggtaaatcagatagcagtgacagtgatagtagtgatagcagcaatagcagtgatagtagtgacagcagtgacagtgacagcagtgatagcaacagtagcagtgatagtgacagcagtgacagtgacagcagtgatagcag

SHap4: gacagtaatggcaatggtaacaatgggaatgatgacaatgacaaatcagacagtggcaaaggtaaatcagatagcagtgacagtgatagtagtgatagcagcaatagcagtgatagtagtgacagcagtgacagtgacagcagtgatagcaacagtagcagtgatagtgacagcagtgacagtgacagcagtgatagcag

SHap5: gacagtaatggcaatggtaacaatgggaatgatgacaatgacaaatcagacagtggcaaaggtaaatcagatagcagtgacagtgatagtagtgatagcagcaatagcagtgatagtagtgacagcagtgacagtgacagcagtgatagcaacagtagcagtgatagtgacagcagtgacagtgacagcagtgatagcag

SHap6: gacagtaatggcaatggtaacaatgggaatgatgacaatgacaaatcagacagtggcaaaggtaaatcagatagcagtgacagtgatagtagtgatagcagcaatagcagtgatagtagtgacagcagtgacagtgacagcagtgatagcaacagtagcagtgatagtgacagcagtgacagtgacagcagtgatagcag

SHap6(2) gacagtaatggcaatggtaacaatgggaatgatgacaatgacaaatcagacagtggcaaaggtaaatcagatagcagtgacagtgatagtagtgatagcagcaatagcagtgatagtagtgacagcagtgacagtgacagcagtgatagcaacagtagcagtgatagtgacagcagtgacagtgacagcagtgatagcag

SHap7 gacagtaatggcaatggtaacaatgggaatgatgacaatgacaaatcagacagtggcaaaggtaaatcagatagcagtgacagtgatagtagtgatagcagcaatagcagtgatagtagtgacagcagtgacagtgacagcagtgatagcaacagtagcagtgatagtgacagcagtgacagtgacagcagtgatagcag

SHap102 gacagtaatggcaatggtaacaatgggaatgatgacaatgacaaatcagacagtggcaaaggtaaatcagatagcagtgacagtgatagtagtgatagcagcaatagcagtgatagtagtgacagcagtgacagtgacagcagtgatagcaacagtagcagtgatagtgacagcagtgacagtgacagcagtgatagcag

SHap130 gacagtaatggcaatggtaacaatgggaatgatgacaatgacaaatcagacagtggcaaaggtaaatcagatagcagtgacagtgatagtagtgatagcagcaatagcagtgatagtagtgacagcagtgacagtgacagcagtgatagcaacagtagcagtgatagtgacagcagtgacagtgacagcagtgatagcag

SHap106 gacagtaatggcaatggtaacaatgggaatgatgacaatgacaaatcagacagtggcaaaggtaaatcagatagcagtgacagtgatagtagtgatagcagcaatagcagtgatagtagtgacagcagtgacagtgacagcagtgatagcaacagtagcagtgatagtgacagcagtgacagtgacagcagtgatagcag

SHap72 gacagtaatggcaatggtaacaatgggaatgatgacaatgacaaatcagacagtggcaaaggtaaatcagatagcagtgacagtgatagtagtgatagcagcaatagcagtgatagtagtgacagcagtgacagtgacagcagtgatagcaacagtagcagtgatagtgacagcagtgacagtgacagcagtgatagcag

SHap110 gacagtaatggcaatggtaacaatgggaatgatgacaatgacaaatcagacagtggcaaaggtaaatcagatagcagtgacagtgatagtagtgatagcagcaatagcagtgatagtagtgacagcagtgacagtgacagcagtgatagcaacagtagcagtgatagtgacagcagtgacagtgacagcagtgatagcag

Merged gacagtaatggcaatggtaacaatgggaatgatgacaatgacaaatcagacagtggcaaaggtaaatcagatagcagtgacagtgatagtagtgatagcagcaatagcagtgatagtagtgacagcagtgacagtgacagcagtgatagcaacagtagcagtgatagtgacagcagtgacagtgacagcagtgatagcag

Transl D S N G N G N N G N D D N D K S D S G K G K S D S S D S D S S D S S N S S D S S D S S D S D S S D S N S S S D S D S S D S D S S D S S 591

**Note:** Dentin dysplasia II: c.1686delT; p.Asp562Glufs*752 (5) Kindred 1.

1773 1830 1870_1873 1918_1921 1922_1925 1972

RefSeq tgacagtgatagtagtgatagcagcaatagcagtgacagtagtgacagcagtgatag**c**agtgacagtagtgatagtagtgacagcagtgacagcaag**tcag**acagcagcaaatcagagagcgacagcagtgatagtgacagtaag**tcagacag**cagtgacagcaacagcagtgacagtagtgacaacagtgatagcagcg

McKnight tgacagtgatagtagtgatagcagcaatagcagtgacagtagtgacagcagtgatagcagtgacagtagtgatagtagtgacagcagtgacagcaagtcagacagcagcaaatcagagagcgacagcagtgatagtgacagtaagtcagacagcagtgacagcaacagcagtgacagtagtgacaacagtgatagcagcg

F1a TGACAGTGATAGTAGTGATAGCAGCAATAGCAGTGACAGTAGTGACAGCAGTGATAGCAGTGACAGTAGTGATAGTAGTGACAGCAGTGACAGCAAGTCAGACAGCAGCAAATCAGAGAGCGACAGCAGTGATAGTGACAGTAAGTCAGACAGCAGTGACAGCAACAGCAGTGACAGTAGTGACAACAGTGATAGCAGCG

F1b TGACAGTGATAGTAGTGATAGCAGCAATAGCAGTGACAGTAGTGACAGCAGTGATAGCAGTGACAGTAGTGATAGTAGTGACAGCAGTGACAGCAAGTCAGACAGCAGCAAATCAGAGAGCGACAGCAGTGATAGTGACAGTAAGTCAGACAGCAGTGACAGCAACAGCAGTGACAGTAGTGACAACAGTGATAGCAGCG

F2a TGACAGTGATAGTAGTGATAGCAGCAATAGCAGTGACAGTAGTGACAGCAGTGATAGCAGTGACAGTAGTGATAGTAGTGACAGCAGTGACAGCAAGTCAGACAGCAGCAAATCAGAGAGCGACAGCAGTGATAGTGACAGTAAGTCAGACAGCAGTGACAGCAACAGCAGTGACAGTAGTGACAACAGTGATAGCAG**T**G

F2b TGACAGTGATAGTAGTGATAGCAGCAATAGCAGTGACAGTAGTGACAGCAGTGATAGCAGTGACAGTAGTGATAGTAGTGACAGCAGTGACAGCAAGTCAGACAGCAGCAAATCAGAGAGCGACAGCAGTGATAGTGACAGTAAGTCAGACAGCAGTGACAGCAACAGCAGTGACAGTAGTGACAACAGTGATAGCAGCG

F3a TGACAGTGATAGTAGTGATAGCAGCAATAGCAGTGACAGTAGTGACAGCAGTGATAGCAGTGACAGTAGTGATAGTAGTGACAGCAGTGACAGCAAGTCAGACAGCAGCAAATCAGAGAGCGACAGCAGTGATAGTGACAGTAAGTCAGACAGCAGTGACAGCAACAGCAGTGACAGTAGTGACAACAGTGATAGCAG**T**G

F3b TGACAGTGATAGTAGTGATAGCAGCAATAGCAGTGACAGTAGTGACAGCAGTGATAGCAGTGACAGTAGTGATAGTAGTGACAGCAGTGACAGCAAGTCAGACAGCAGCAAATCAGAGAGCGACAGCAGTGATAGTGACAGTAAGTCAGACAGCAGTGACAGCAACAGCAGTGACAGTAGTGACAACAGTGATAGCAGCG

F4a TGACAGTGATAGTAGTGATAGCAGCAATAGCAGTGACAGTAGTGACAGCAGTGATAGCAGTGACAGTAGTGATAGTAGTGACAGCAGTGACAGCAAGTCAGACAGCAGCAAATCAGAGAGCGACAGCAGTGATAGTGACAGTAAGTCAGACAGCAGTGACAGCAACAGCAGTGACAGTAGTGACAACAGTGATAGCAGCG

F4b TGACAGTGATAGTAGTGATAGCAGCAATAGCAGTGACAGTAGTGACAGCAGTGATAGCAGTGACAGTAGTGATAGTAGTGACAGCAGTGACAGCAAGTCAGACAGCAGCAAATCAGAGAGCGACAGCAGTGATAGTGACAGTAAGTCAGACAGCAGTGACAGCAACAGCAGTGACAGTAGTGACAACAGTGATAGCAGCG

F5a TGACAGTGATAGTAGTGATAGCAGCAATAGCAGTGACAGTAGTGACAGCAGTGATAGCAGTGACAGTAGTGATAGTAGTGACAGCAGTGACAGCAAGTCAGACAGCAGCAAATCAGAGAGCGACAGCAGTGATAGTGACAGTAAGTCAGACAGCAGTGACAGCAACAGCAGTGACAGTAGTGACAACAGTGATAGCAGCG

F5b TGACAGTGATAGTAGTGATAGCAGCAATAGCAGTGACAGTAGTGACAGCAGTGATAGCAGTGACAGTAGTGATAGTAGTGACAGCAGTGACAGCAAGTCAGACAGCAGCAAATCAGAGAGCGACAGCAGTGATAGTGACAGTAAGTCAGACAGCAGTGACAGCAACAGCAGTGACAGTAGTGACAACAGTGATAGCAG**T**G

Hap1a TGACAGTGATAGTAGTGATAGCAGCAATAGCAGTGACAGTAGTGACAGCAGTGATAGCAGTGACAGTAGTGATAGTAGTGACAGCAGTGACAGCAAGTCAGACAGCAGCAAATCAGAGAGCGACAGCAGTGATAGTGACAGTAAGTCAGACAGCAGTGACAGCAACAGCAGTGACAGTAGTGACAACAGTGATAGCAGCG

Hap2a TGACAGTGATAGTAGTGATAGCAGCAATAGCAGTGACAGTAGTGACAGCAGTGATAGCAGTGACAGTAGTGATAGTAGTGACAGCAGTGACAGCAAGTCAGACAGCAGCAAATCAGAGAGCGACAGCAGTGATAGTGACAGTAAGTCAGACAGCAGTGACAGCAACAGCAGTGACAGTAGTGACAACAGTGATAGCAGCG

Hap3a TGACAGTGATAGTAGTGATAGCAGCAATAGCAGTGACAGTAGTGACAGCAGTGATAGCAGTGACAGTAGTGATAGTAGTGACAGCAGTGACAGCAAGTCAGACAGCAGCAAATCAGAGAGCGACAGCAGTGATAGTGACAGTAAGTCAGACAGCAGTGACAGCAACAGCAGTGACAGTAGTGACAACAGTGATAGCAGCG

Hap15a TGACAGTGATAGTAGTGATAGCAGCAATAGCAGTGACAGTAGTGACAGCAGTGATAGCAGTGACAGTAGTGATAGTAGTGACAGCAGTGACAGCAAGTCAGACAGCAGCAAATCAGAGAGCGACAGCAGTGATAGTGACAGTAAGTCAGACAGCAGTGACAGCAACAGCAGTGACAGTAGTGACAACAGTGATAGCAG**T**G

Hap17b tgacagtgatagtagtgatagcagcaatagcagtgacagtagtgacagcagtgatagcagtgacagtagtgatagtagtgacagcagtgacagcaagtcagacagcagcaaatcagagagcgacagcagtgatagtgacagtaagtcagacagcagtgacagcaacagcagtgacagtagtgacaacagtgatagcagcg

Hap20a TGACAGTGATAGTAGTGATAGCAGCAATAGCAGTGACAGTAGTGACAGCAGTGATAGCAGTGACAGTAGTGATAGTAGTGACAGCAGTGACAGCAAGTCAGACAGCAGCAAATCAGAGAGCGACAGCAGTGATAGTGACAGTAAGTCAGACAGCAGTGACAGCAACAGCAGTGACAGTAGTGACAACAGTGATAGCAGCG

Hap20b tgacagtgatagtagtgatagcagcaatagcagtgacagtagtgacagcagtgatagcagtgacagtagtgatagtagtgacagcagtgacagcaagtcagacagcagcaaatcagagagcgacagcagtgatagtgacagtaagtcagacagcagtgacagcaacagcagtgacagtagtgacaacagtgatagcagcg

Hap36a TGACAGTGATAGTAGTGATAGCAGCAATAGCAGTGACAGTAGTGACAGCAGTGATAGCAGTGACAGTAGTGATAGTAGTGACAGCAGTGACAGCAAGTCAGACAGCAGCAAATCAGAGAGCGACAGCAGTGATAGTGACAGTAAGTCAGACAGCAGTGACAGCAACAGCAGTGACAGTAGTGACAACAGTGATAGCAGCG

Hap37a TGACAGTGATAGTAGTGATAGCAGCAATAGCAGTGACAGTAGTGACAGCAGTGATAGCAGTGACAGTAGTGATAGTAGTGACAGCAGTGACAGCAAGTCAGACAGCAGCAAATCAGAGAGCGACAGCAGTGATAGTGACAGTAAGTCAGACAGCAGTGACAGCAACAGCAGTGACAGTAGTGACAACAGTGATAGCAGCG

Hap38a TGACAGTGATAGTAGTGATAGCAGCAATAGCAGTGACAGTAGTGACAGCAGTGATAGCAGTGACAGTAGTGATAGTAGTGACAGCAGTGACAGCAAGTCAGACAGCAGCAAATCAGAGAGCGACAGCAGTGATAGTGACAGTAAGTCAGACAGCAGTGACAGCAACAGCAGTGACAGTAGTGACAACAGTGATAGCAGCG

SHap1: tgacagtgatagtagtgatagcagcaatagcagtgacagtagtgacagcagtgatagcagtgacagtagtgatagtagtgacagcagtgacagcaagtcagacagcagcaaatcagagagcgacagcagtgatagtgacagtaagtcagacagcagtgacagcaacagcagtgacagtagtgacaacagtgatagcagcg

SHap2: tgacagtgatagtagtgatagcagcaatagcagtgacagtagtgacagcagtgatagcagtgacagtagtgatagtagtgacagcagtgacagcaagtcagacagcagcaaatcagagagcgacagcagtgatagtgacagtaagtcagacagcagtgacagcaacagcagtgacagtagtgacaacagtgatagcagcg

SHap3: tgacagtgatagtagtgatagcagcaatagcagtgacagtagtgacagcagtgatagcagtgacagtagtgatagtagtgacagcagtgacagcaagtcagacagcagcaaatcagagagcgacagcagtgatagtgacagtaagtcagacagcagtgacagcaacagcagtgacagtagtgacaacagtgatagcag**t**g

SHap4: tgacagtgatagtagtgatagcagcaatagcagtgacagtagtgacagcagtgatagcagtgacagtagtgatagtagtgacagcagtgacagcaagtcagacagcagcaaatcagagagcgacagcagtgatagtgacagtaagtcagacagcagtgacagcaacagcagtgacagtagtgacaacagtgatagcagcg

SHap5: tgacagtgatagtagtgatagcagcaatagcagtgacagtagtgacagcagtgatagcagtgacagtagtgatagtagtgacagcagtgacagcaagtcagacagcagcaaatcagagagcgacagcagtgatagtgacagtaagtcagacagcagtgacagcaacagcagtgacagtagtgacaacagtgatagcagcg

SHap6: tgacagtgatagtagtgatagcagcaatagcagtgacagtagtgacagcagtgatagcagtgacagtagtgatagtagtgacagcagtgacagcaagtcagacagcagcaaatcagagagcgacagcagtgatagtgacagtaagtcagacagcagtgacagcaacagcagtgacagtagtgacaacagtgatagcagcg

SHap6(2) tgacagtgatagtagtgatagcagcaatagcagtgacagtagtgacagcagtgatagcagtgacagtagtgatagtagtgacagcagtgacagcaagtcagacagcagcaaatcagagagcgacagcagtgatagtgacagtaagtcagacagcagtgacagcaacagcagtgacagtagtgacaacagtgatagcagcg

SHap7 tgacagtgatagtagtgatagcagcaatagcagtgacagtagtgacagcagtgatagcagtgacagtagtgatagtagtgacagcagtgacagcaagtcagacagcagcaaatcagagagcgacagcagtgatagtgacagtaagtcagacagcagtgacagcaacagcagtgacagtagtgacaacagtgatagcagcg

SHap102 tgacagtgatagtagtgatagcagcaatagcagtgacagtagtgacagcagtgatagcagtgacagtagtgatagtagtgacagcagtgacagcaagtcagacagcagcaaatcagagagcgacagcagtgatagtgacagtaagtcagacagcagtgacagcaacagcagtgacagtagtgacaacagtgatagcagcg

SHap130 tgacagtgatagtagtgatagcagcaatagcagtgacagtagtgacagcagtgatagcagtgacagtagtgatagtagtgacagcagtgacagcaagtcagacagcagcaaatcagagagcgacagcagtgatagtgacagtaagtcagacagcagtgacagcaacagcagtgacagtagtgacaacagtgatagcagcg

SHap106 tgacagtgatagtagtgatagcagcaatagcagtgacagtagtgacagcagtgatagcagtgacagtagtgatagtagtgacagcagtgacagcaagtcagacagcagcaaatcagagagcgacagcagtgatagtgacagtaagtcagacagcagtgacagcaacagcagtgacagtagtgacaacagtgatagcagcg

SHap72 tgacagtgatagtagtgatagcagcaatagcagtgacagtagtgacagcagtgatagcagtgacagtagtgatagtagtgacagcagtgacagcaagtcagacagcagcaaatcagagagcgacagcagtgatagtgacagtaagtcagacagcagtgacagcaacagcagtgacagtagtgacaacagtgatagcagcg

SHap110 tgacagtgatagtagtgatagcagcaatagcagtgacagtagtgacagcagtgatagcagtgacagtagtgatagtagtgacagcagtgacagcaagtcagacagcagcaaatcagagagcgacagcagtgatagtgacagtaagtcagacagcagtgacagcaacagcagtgacagtagtgacaacagtgatagcagcg

Merged tgacagtgatagtagtgatagcagcaatagcagtgacagtagtgacagcagtgatagcagtgacagtagtgatagtagtgacagcagtgacagcaagtcagacagcagcaaatcagagagcgacagcagtgatagtgacagtaagtcagacagcagtgacagcaacagcagtgacagtagtgacaacagtgatagcagcg

Transl D S D S S D S S N S S D S S D S S D S S D S S D S S D S S D S K S D S S K S E S D S S D S D S K S D S S D S N S S D S S D N S D S S 657

**Notes:** Dentin dysplasia II: c.1830delC; p.Ser610Argfs*704 (5) Kindred 2.

Dentin dysplasia II: c.1870_1873delTCAG; p.Ser624Thrfs*689 (2).

Dentin dysplasia II: c.1918_1921delTCAG; p.Ser640Thrfs*673 (2).

Dentin dysplasia II: c.1918_1921delTCAG; p.Ser640Thrfs*673 (5) Kindreds 3-5.

Dentin dysplasia II: c.1922_1925delACAG; p.Asp641Alafs*672 (5) Kindred 6.

Sequences highlighted in cyan correspond to sequence variations associated with dentin dysplasia type II (DD-II).

Sequences in bold are single nucleotide polymorphisms (SNPs): c.1971C>T. The C or T at position 1971 is the first sequence variation in the DPP coding region among the haplotypes.

1973 ***ID1*** 2040 ***ID2*** 2063 2154

RefSeq acagcagcaatagcagtaacagcagtgatagtagtgacagcagtgatagcagtgacagcagcagtag**c**agtgacagcagca------------------acagcagtg**a**tagtagtgacagtagtgacagcagcaatagcagtgagagcagtgatagtagtgacagcagtgatagtgacagcagtgatagtagtgacagc

McKnight acagcagcaatagcagtaacagcagtgatagtagtgacagcagtgatagcagtgacagcagcagtagcagtgacagcagcagtagcagtgacagcagcaacagcagtgatagtagtgacagtagtgacagcagcaatagcagtgagagcagtgatagtagtgacagcagtgatagtgacagcagtgatagtagtgacagc

F1a ACAGCAGCAATAGCAGTAACAGCAGTGATAGTAGTGACAGCAGTGATAGCAGTGACAGCAGCAGTAGCAGTGACAGCAGCAGTAGCAGTGACAGCAGCAACAGCAGTGATAGTAGTGACAGTAGTGACAGCAGCAATAGCAGTGAGAGCAGTGATAGTAGTGACAGCAGTGATAGTGACAGCAGTGATAGTAGTGACAGC

F1b ACAGCAGCAATAGCAGTAACAGCAGTGATAGTAGTGACAGCAGTGATAGCAGTGACAGCAGCAGTAGCAGTGACAGCAGCAGTAGCAGTGACAGCAGCAACAGCAGTGATAGTAGTGACAGTAGTGACAGCAGCAATAGCAGTGAGAGCAGTGATAGTAGTGACAGCAGTGATAGTGACAGCAGTGATAGTAGTGACAGC

F2a ACAGCAGCAATAGCAGTAACAGCAGTGATAGTAGTGACAGCAGTGATAGCAGTGACAGCAGCAGTAGCAGTGACAGCAGCA------------------ACAGCAGTGATAGTAGTGACAGTAGTGACAGCAGCAATAGCAGTGAGAGCAGTGATAGTAGTGACAGCAGTGATAGTGACAGCAGTGATAGTAGTGACAGC

F2b ACAGCAGCAATAGCAGTAACAGCAGTGATAGTAGTGACAGCAGTGATAGCAGTGACAGCAGCAGTAGCAGTGACAGCAGCA------------------ACAGCAGTGATAGTAGTGACAGTAGTGACAGCAGCAATAGCAGTGAGAGCAGTGATAGTAGTGACAGCAGTGATAGTGACAGCAGTGATAGTAGTGACAGC

F3a ACAGCAGCAATAGCAGTAACAGCAGTGATAGTAGTGACAGCAGTGATAGCAGTGACAGCAGCAGTAGCAGTGACAGCAGCA------------------ACAGCAGTGATAGTAGTGACAGTAGTGACAGCAGCAATAGCAGTGAGAGCAGTGATAGTAGTGACAGCAGTGATAGTGACAGCAGTGATAGTAGTGACAGC

F3b ACAGCAGCAATAGCAGTAACAGCAGTGATAGTAGTGACAGCAGTGATAGCAGTGACAGCAGCAGTAGCAGTGACAGCAGCA------------------ACAGCAGTGATAGTAGTGACAGTAGTGACAGCAGCAATAGCAGTGAGAGCAGTGATAGTAGTGACAGCAGTGATAGTGACAGCAGTGATAGTAGTGACAGC

F4a ACAGCAGCAATAGCAGTAACAGCAGTGATAGTAGTGACAGCAGTGATAGCAGTGACAGCAGCAGTAGCAGTGACAGCAGCA------------------ACAGCAGTGATAGTAGTGACAGTAGTGACAGCAGCAATAGCAGTGAGAGCAGTGATAGTAGTGACAGCAGTGATAGTGACAGCAGTGATAGTAGTGACAGC

F4b ACAGCAGCAATAGCAGTAACAGCAGTGATAGTAGTGACAGCAGTGATAGCAGTGACAGCAGCAGTAGCAGTGACAGCAGCA------------------ACAGCAGTGATAGTAGTGACAGTAGTGACAGCAGCAATAGCAGTGAGAGCAGTGATAGTAGTGACAGCAGTGATAGTGACAGCAGTGATAGTAGTGACAGC

F5a ACAGCAGCAATAGCAGTAACAGCAGTGATAGTAGTGACAGCAGTGATAGCAGTGACAGCAGCAGTAGCAGTGACAGCAGCA------------------ACAGCAGTGATAGTAGTGACAGTAGTGACAGCAGCAATAGCAGTGAGAGCAGTGATAGTAGTGACAGCAGTGATAGTGACAGCAGTGATAGTAGTGACAGC

F5b ACAGCAGCAATAGCAGTAACAGCAGTGATAGTAGTGACAGCAGTGATAGCAGTGACAGCAGCAGTAGCAGTGACAGCAGCA------------------ACAGCAGTGATAGTAGTGACAGTAGTGACAGCAGCAATAGCAGTGAGAGCAGTGATAGTAGTGACAGCAGTGATAGTGACAGCAGTGATAGTAGTGACAGC

Hap1a ACAGCAGCAATAGCAGTAACAGCAGTGATAGTAGTGACAGCAGTGATAGCAGTGACAGCAGCAGTAGCAGTGACAGCAGCAGTAGCAGTGACAGCAGCAACAGCAGTGATAGTAGTGACAGTAGTGACAGCAGCAATAGCAGTGAGAGCAGTGATAGTAGTGACAGCAGTGATAGTGACAGCAGTGATAGTAGTGACAGC

Hap2a ACAGCAGCAATAGCAGTAACAGCAGTGATAGTAGTGACAGCAGTGATAGCAGTGACAGCAGCAGTAGCAGTGACAGCAGCA------------------ACAGCAGTGATAGTAGTGACAGTAGTGACAGCAGCAATAGCAGTGAGAGCAGTGATAGTAGTGACAGCAGTGATAGTGACAGCAGTGATAGTAGTGACAGC

Hap3a ACAGCAGCAATAGCAGTAACAGCAGTGATAGTAGTGACAGCAGTGATAGCAGTGACAGCAGCAGTAGCAGTGACAGCAGCA------------------ACAGCAGTGATAGTAGTGACAGTAGTGACAGCAGCAATAGCAGTGAGAGCAGTGATAGTAGTGACAGCAGTGATAGTGACAGCAGTGATAGTAGTGACAGC

Hap15a ACAGCAGCAATAGCAGTAACAGCAGTGATAGTAGTGACAGCAGTGATAGCAGTGACAGCAGCAGTAGCAGTGACAGCAGCA------------------ACAGCAGTGATAGTAGTGACAGTAGTGACAGCAGCAATAGCAGTGAGAGCAGTGATAGTAGTGACAGCAGTGATAGTGACAGCAGTGATAGTAGTGACAGC

Hap17b acagcagcaatagcagtaacagcagtgatagtagtgacagcagtgatagcagtgacagcagcagtagcagtgacagcagcagtagcagtgacagcagcaacagcagtgatagtagtgacagtagtgacagcagcaatagcagtgagagcagtgatagtagtgacagcagtgatagtgacagcagtgatagtagtgacagc

Hap20a ACAGCAGCAATAGCAGTAACAGCAGTGATAGTAGTGACAGCAGTGATAGCAGTGACAGCAGCAGTAGCAGTGACAGCAGCA------------------ACAGCAGTGATAGTAGTGACAGTAGTGACAGCAGCAATAGCAGTGAGAGCAGTGATAGTAGTGACAGCAGTGATAGTGACAGCAGTGATAGTAGTGACAGC

Hap20b acagcagcaatagcagtaacagcagtgatagtagtgacagcagtgatagcagtgacagcagcagtagcagtgacagcagca------------------acagcagtgatagtagtgacagtagtgacagcagcaatagcagtgagagcagtgatagtagtgacagcagtgatagtgacagcagtgatagtagtgacagc

Hap36a ACAGCAGCAATAGCAGTAACAGCAGTGATAGTAGTGACAGCAGTGATAGCAGTGACAGCAGC------AGTGACAGCAGCA------------------ACAGCAGTGATAGTAGTGACAGTAGTGACAGCAGCAATAGCAGTGAGAGCAGTGATAGTAGTGACAGCAGTGATAGTGACAGCAGTGATAGTAGTGACAGC

Hap37a ACAGCAGCAATAGCAGTAACAGCAGTGATAGTAGTGACAGCAGTGATAGCAGTGACAGCAGCAGTAGCAGTGACAGCAGCA------------------ACAGCAGTGATAGTAGTGACAGTAGTGACAGCAGCAATAGCAGTGAGAGCAGTGATAGTAGTGACAGCAGTGATAGTGACAGCAGTGATAGTAGTGACAGC

Hap38a ACAGCAGCAATAGCAGTAACAGCAGTGATAGTAGTGACAGCAGTGATAGCAGTGACAGCAGCAGTAGCAGTGACAGCAGCA------------------ACAGCAGTGATAGTAGTGACAGTAGTGACAGCAGCAATAGCAGTGAGAGCAGTGATAGTAGTGACAGCAGTGATAGTGACAGCAGTGATAGTAGTGACAGC

SHap1: acagcagcaatagcagtaacagcagtgatagtagtgacagcagtgatagcagtgacagcagcagtagcagtgacagcagcagtagcagtgacagcagcaacagcagtgatagtagtgacagtagtgacagcagcaatagcagtgagagcagtgatagtagtgacagcagtgatagtgacagcagtgatagtagtgacagc

SHap2: acagcagcaatagcagtaacagcagtgatagtagtgacagcagtgatagcagtgacagcagcagtagcagtgacagcagca------------------acagcagtgatagtagtgacagtagtgacagcagcaatagcagtgagagcagtgatagtagtgacagcagtgatagtgacagcagtgatagtagtgacagc

SHap3: acagcagcaatagcagtaacagcagtgatagtagtgacagcagtgatagcagtgacagcagcagtagcagtgacagcagca------------------acagcagtgatagtagtgacagtagtgacagcagcaatagcagtgagagcagtgatagtagtgacagcagtgatagtgacagcagtgatagtagtgacagc

SHap4: acagcagcaatagcagtaacagcagtgatagtagtgacagcagtgatagcagtgacagcagcagtagcagtgacagcagca------------------acagcagtgatagtagtgacagtagtgacagcagcaatagcagtgagagcagtgatagtagtgacagcagtgatagtgacagcagtgatagtagtgacagc

SHap5: acagcagcaatagcagtaacagcagtgatagtagtgacagcagtgatagcagtgacagcagcagtagcagtgacagcagcagtagcagtgacagcagcaacagcagtgatagtagtgacagtagtgacagcagcaatagcagtgagagcagtgatagtagtgacagcagtgatagtgacagcagtgatagtagtgacagc

SHap6: acagcagcaatagcagtaacagcagtgatagtagtgacagcagtgatagcagtgacagcagcagtagcagtgacagcagcagtagcagtgacagcagcaacagcagtgatagtagtgacagtagtgacagcagcaatagcagtgagagcagtgatagtagtgacagcagtgatagtgacagcagtgatagtagtgacagc

SHap6(2) acagcagcaatagcagtaacagcagtgatagtagtgacagcagtgatagcagtgacagcagcagtagcagtgacagcagcagtagcagtgacagcagcaacagcagtgatagtagtgacagtagtgacagcagcaatagcagtgagagcagtgatagtagtgacagcagtgatagtgacagcagtgatagtagtgacagc

SHap7 acagcagcaatagcagtaacagcagtgatagtagtgacagcagtgatagcagtgacagcagcagtagcagtgacagcagca------------------acagcagtgatagtagtgacagtagtgacagcagcaatagcagtgagagcagtgatagtagtgacagcagtgatagtgacagcagtgatagtagtgacagc

SHap102 acagcagcaatagcagtaacagcagtgatagtagtgacagcagtgatagcagtgacagcagcagtagcagtgacagcagca------------------acagcagtgatagtagtgacagtagtgacagcagcaatagcagtgagagcagtgatagtagtgacagcagtgatagtgacagcagtgatagtagtgacagc

SHap130 acagcagcaatagcagtaacagcagtgatagtagtgacagcagtgatagcagtgacagcagcagtagcagtgacagcagcagtagcagtgacagcagcaacagcagtgatagtagtgacagtagtgacagcagcaatagcagtgagagcagtgatagtagtgacagcagtgatagtgacagcagtgatagtagtgacagc

SHap106 acagcagcaatagcagtaacagcagtgatagtagtgacagcagtgatagcagtgacagcagcagtagcagtgacagcagcagtagcagtgacagcagcaacagcagtgatagtagtgacagtagtgacagcagcaatagcagtgagagcagtgatagtagtgacagcagtgatagtgacagcagtgatagtagtgacagc

SHap72 acagcagcaatagcagtaacagcagtgatagtagtgacagcagtgatagcagtgacagcagcagtagcagtgacagcagca------------------acagcagtgatagtagtgacagtagtgacagcagcaatagcagtgagagcagtgatagtagtgacagcagtgatagtgacagcagtgatagtagtgacagc

SHap110 acagcagcaatagcagtaacagcagtgatagtagtgacagcagtgatagcagtgacagcagcagtagcagtgacagcagca------------------acagcagtgatagtagtgacagtagtgacagcagcaatagcagtgagagcagtgatagtagtgacagcagtgatagtgacagcagtgatagtagtgacagc

Merged acagcagcaatagcagtaacagcagtgatagtagtgacagcagtgatagcagtgacagcagcagtagcagtgacagcagca**gtagcagtgacagcagca**acagcagtgatagtagtgacagtagtgacagcagcaatagcagtgagagcagtgatagtagtgacagcagtgatagtgacagcagtgatagtagtgacagc

Transl D S S N S S N S S D S S D S S D S S D S S S S S D S S **S S S D S S**  N S S D S S D S S D S S N S S E S S D S S D S S D S D S S D S S D S 718

**Note:** Dentin dysplasia II: c.2040delC; p.Ser680Argfs*634 (4) Family H.

Dentin dysplasia II: c.2063delA; p.Asp688Valfs*626 (5) Kindreds 7 & 8.

Bold merged or translated sequences are not found in the *DSPP* cDNA reference sequence.

***ID1***: NM_014208.3:c.2035_2040del AGTAGC.

***ID2***: NM_014208.3:c.2053_2054insGTAGCAGTGACAGCAGCA.

2155 ***ID3*** 2272 ***ID4*** 2349 2354

RefSeq agtaatagtaacagcagcgatagtgacagcagcaacagcagcgatagcagtgacagcagcaacagcagtgacagcagtgatagcagtgacagcagcaacagcagtgacagtagcgat**a**gcagtgacagcagcaacagcagtgacagcagtgatagcagtgacagcagtgatagtagtgacagcagcaacagcag**t**gatag

McKnight agtaatagtaacagcagcgatagtgacagcagcaacagcagcgatagcagtgacagcagcaacagcagtgacagcagtgatagcagtgacagcagcaacagcagtgacagtagcgatagcagtgacagcagcaacagcagtgacagcagtgatagcagtgacagcagtgatagtagtgacagcagcaacagcagtgatag

F1a AGTAATAGTAACAGCAGCGATAGTGACAGCAGCAACAGCAGCGATAGCAGTGACAGCAGCAACAGCAGTGACAGCAGTGATAGCAGTGACAGCAGCAACAGCAGTGACAGTAGCGATAGCAGTGACAGCAGCAACAGCAGTGACAGCAGTGATAGCAGTGACAGCAGTGATAGTAGTGACAGCAGCAACAGCAGTGATAG

F1b AGTAATAGTAACAGCAGCGATAGTGACAGCAGCAACAGCAGCGATAGCAGTGACAGCAG------------------TGATAGCAGTGACAGCAGCAACAGCAGTGACAGTAGCGATAGCAGTGACAGCAGCAACAGCAGTGACAGCAGTGATAGCAGTGACAGCAGTGATAGTAGTGACAGCAGCAACAGCAGTGATAG

F2a AGTAATAGTAACAGCAGCGATAGTGACAGCAGCAACAGCAGCGATAGCAGTGACAGCAGCAACAGCAGTGACAGCAGTGATAGCAGTGACAGCAGCAACAGCAGTGACAGTAGCGATAGCAGTGACAGCAGCAACAGCAGTGACAGCAGTGATAGCAGTGACAGCAGTGATAGTAGTGACAGCAGCAACAGCAGTGATAG

F2b AGTAATAGTAACAGCAGCGATAGTGACAGCAGCAACAGCAGCGATAGCAGTGACAGCAGCAACAGCAGTGACAGCAGTGATAGCAGTGACAGCAGCAACAGCAGTGACAGTAGCGATAGCAGTGACAGCAGCAACAGCAGTGACAGCAGTGATAGCAGTGACAGCAGTGATAGTAGTGACAGCAGCAACAGCAGTGATAG

F3a AGTAATAGTAACAGCAGCGATAGTGACAGCAGCAACAGCAGCGATAGCAGTGACAGCAGCAACAGCAGTGACAGCAGTGATAGCAGTGACAGCAGCAACAGCAGTGACAGTAGCGATAGCAGTGACAGCAGCAACAGCAGTGACAGCAGTGATAGCAGTGACAGCAGTGATAGTAGTGACAGCAGCAACAGCAGTGATAG

F3b AGTAATAGTAACAGCAGCGATAGTGACAGCAGCAACAGCAGCGATAGCAGTGACAGCAGCAACAGCAGTGACAGCAGTGATAGCAGTGACAGCAGCAACAGCAGTGACAGTAGCGATAGCAGTGACAGCAGCAACAGCAGTGACAGCAGTGATAGCAGTGACAGCAGTGATAGTAGTGACAGCAGCAACAGCAGTGATAG

F4a AGTAATAGTAACAGCAGCGATAGTGACAGCAGCAACAGCAGCGATAGCAGTGACAGCAGCAACAGCAGTGACAGCAGTGATAGCAGTGACAGCAGCAACAGCAGTGACAGTAGCGATAGCAGTGACAGCAG------------------TGATAGCAGTGACAGCAGTGATAGTAGTGACAGCAGCAACAGCAGTGATAG

F4b AGTAATAGTAACAGCAGCGATAGTGACAGCAGCAACAGCAGCGATAGCAGTGACAGCAGCAACAGCAGTGACAGCAGTGATAGCAGTGACAGCAGCAACAGCAGTGACAGTAGCGATAGCAGTGACAGCAGCAACAGCAGTGACAGCAGTGATAGCAGTGACAGCAGTGATAGTAGTGACAGCAGCAACAGCAGTGATAG

F5a AGTAATAGTAACAGCAGCGATAGTGACAGCAGCAACAGCAGCGATAGCAGTGACAGCAGCAACAGCAGTGACAGCAGTGATAGCAGTGACAGCAGCAACAGCAGTGACAGTAGCGATAGCAGTGACAGCAGCAACAGCAGTGACAGCAGTGATAGCAGTGACAGCAGTGATAGTAGTGACAGCAGCAACAGCAGTGATAG

F5b AGTAATAGTAACAGCAGCGATAGTGACAGCAGCAACAGCAGCGATAGCAGTGACAGCAGCAACAGCAGTGACAGCAGTGATAGCAGTGACAGCAGCAACAGCAGTGACAGTAGCGATAGCAGTGACAGCAGCAACAGCAGTGACAGCAGTGATAGCAGTGACAGCAGTGATAGTAGTGACAGCAGCAACAGCAGTGATAG

Hap1a AGTAATAGTAACAGCAGCGATAGTGACAGCAGCAACAGCAGCGATAGCAGTGACAGCAG------------------TGATAGCAGTGACAGCAGCAACAGCAGTGACAGTAGCGATAGCAGTGACAGCAGCAACAGCAGTGACAGCAGTGATAGCAGTGACAGCAGTGATAGTAGTGACAGCAGCAACAGCAGTGATAG

Hap2a AGTAATAGTAACAGCAGCGATAGTGACAGCAGCAACAGCAGCGATAGCAGTGACAGCAGCAACAGCAGTGACAGCAGTGATAGCAGTGACAGCAGCAACAGCAGTGACAGTAGCGATAGCAGTGACAGCAGCAACAGCAGTGACAGCAGTGATAGCAGTGACAGCAGTGATAGTAGTGACAGCAGCAACAGCAGTGATAG

Hap3a AGTAATAGTAACAGCAGCGATAGTGACAGCAGCAACAGCAGCGATAGCAGTGACAGCAGCAACAGCAGTGACAGCAGTGATAGCAGTGACAGCAGCAACAGCAGTGACAGTAGCGATAGCAGTGACAGCAGCAACAGCAGTGACAGCAGTGATAGCAGTGACAGCAGTGATAGTAGTGACAGCAGCAACAGCAGTGATAG

Hap15a AGTAATAGTAACAGCAGCGATAGTGACAGCAGCAACAGCAGCGATAGCAGTGACAGCAGCAACAGCAGTGACAGCAGTGATAGCAGTGACAGCAGCAACAGCAGTGACAGTAGCGATAGCAGTGACAGCAGCAACAGCAGTGACAGCAGTGATAGCAGTGACAGCAGTGATAGTAGTGACAGCAGCAACAGCAGTGATAG

Hap17b agtaatagtaacagcagcgatagtgacagcagcaacagcagcgatagcagtgacagcagcaacagcagtgacagcagtgatagcagtgacagcagcaacagcagtgacagtagcgatagcagtgacagcagcaacagcagtgacagcagtgatagcagtgacagcagtgatagtagtgacagcagcaacagcagtgatag

Hap20a AGTAATAGTAACAGCAGCGATAGTGACAGCAGCAACAGCAGCGATAGCAGTGACAGCAGCAACAGCAGTGACAGCAGTGATAGCAGTGACAGCAGCAACAGCAGTGACAGTAGCGATAGCAGTGACAGCAGCAACAGCAGTGACAGCAGTGATAGCAGTGACAGCAGTGATAGTAGTGACAGCAGCAACAGCAGTGATAG

Hap20b agtaatagtaacagcagcgatagtgacagcagcaacagcagcgatagcagtgacagcagcaacagcagtgacagcagtgatagcagtgacagcagcaacagcagtgacagtagcgatagcagtgacagcagcaacagcagtgacagcagtgatagcagtgacagcagtgatagtagtgacagcagcaacagcagtgatag

Hap36a AGTAATAGTAACAGCAGCGATAGTGACAGCAGCAACAGCAGCGATAGCAGTGACAGCAGCAACAGCAGTGACAGCAGTGATAGCAGTGACAGCAGCAACAGCAGTGACAGTAGCGATAGCAGTGACAGCAGCAACAGCAGTGACAGCAGTGATAGCAGTGACAGCAGTGATAGTAGTGACAGCAGCAACAGCAGTGATAG

Hap37a AGTAATAGTAACAGCAGCGATAGTGACAGCAGCAACAGCAGCGATAGCAGTGACAGCAGCAACAGCAGTGACAGCAGTGATAGCAGTGACAGCAGCAACAGCAGTGACAGTAGCGATAGCAGTGACAGCAGCAACAGCAGTGACAGCAGTGATAGCAGTGACAGCAGTGATAGTAGTGACAGCAGCAACAGCAGTGATAG

Hap38a AGTAATAGTAACAGCAGCGATAGTGACAGCAGCAACAGCAGCGATAGCAGTGACAGCAGCAACAGCAGTGACAGCAGTGATAGCAGTGACAGCAGCAACAGCAGTGACAGTAGCGATAGCAGTGACAGCAGCAACAGCAGTGACAGCAGTGATAGCAGTGACAGCAGTGATAGTAGTGACAGCAGCAACAGCAGTGATAG

SHap1: agtaatagtaacagcagcgatagtgacagcagcaacagcagcgatagcagtgacagcagcaacagcagtgacagcagtgatagcagtgacagcagcaacagcagtgacagtagcgatagcagtgacagcagcaacagcagtgacagcagtgatagcagtgacagcagtgatagtagtgacagcagcaacagcagtgatag

SHap2: agtaatagtaacagcagcgatagtgacagcagcaacagcagcgatagcagtgacagcagcaacagcagtgacagcagtgatagcagtgacagcagcaacagcagtgacagtagcgatagcagtgacagcagcaacagcagtgacagcagtgatagcagtgacagcagtgatagtagtgacagcagcaacagcagtgatag

SHap3: agtaatagtaacagcagcgatagtgacagcagcaacagcagcgatagcagtgacagcagcaacagcagtgacagcagtgatagcagtgacagcagcaacagcagtgacagtagcgatagcagtgacagcagcaacagcagtgacagcagtgatagcagtgacagcagtgatagtagtgacagcagcaacagcagtgatag

SHap4: agtaatagtaacagcagcgatagtgacagcagcaacagcagcgatagcagtgacagcagcaacagcagtgacagcagtgatagcagtgacagcagcaacagcagtgacagtagcgatagcagtgacagcagcaacagcagtgacagcagtgatagcagtgacagcagtgatagtagtgacagcagcaacagcagtgatag

SHap5: agtaatagtaacagcagcgatagtgacagcagcaacagcagcgatagcagtgacagcagcaacagcagtgacagcagtgatagcagtgacagcagcaacagcagtgacagtagcgatagcagtgacagcagcaacagcagtgacagcagtgatagcagtgacagcagtgatagtagtgacagcagcaacagcagtgatag

SHap6: agtaatagtaacagcagcgatagtgacagcagcaacagcagcgatagcagtgacagcagcaacagcagtgacagcagtgatagcagtgacagcagcaacagcagtgacagtagcgatagcagtgacagcagcaacagcagtgacagcagtgatagcagtgacagcagtgatagtagtgacagcagcaacagcagtgatag

SHap6(2) agtaatagtaacagcagcgatagtgacagcagcaacagcagcgatagcagtgacagcagcaacagcagtgacagcagtgatagcagtgacagcagcaacagcagtgacagtagcgatagcagtgacagcagcaacagcagtgacagcagtgatagcagtgacagcagtgatagtagtgacagcagcaacagcagtgatag

SHap7 agtaatagtaacagcagcgatagtgacagcagcaacagcagcgatagcagtgacagcagcaacagcagtgacagcagtgatagcagtgacagcagcaacagcagtgacagtagcgatagcagtgacagcagcaacagcagtgacagcagtgatagcagtgacagcagtgatagtagtgacagcagcaacagcagtgatag

SHap102 agtaatagtaacagcagcgatagtgacagcagcaacagcagcgatagcagtgacagcagcaacagcagtgacagcagtgatagcagtgacagcagcaacagcagtgacagtagcgatagcagtgacagcagcaacagcagtgacagcagtgatagcagtgacagcagtgatagtagtgacagcagcaacagcagtgatag

SHap130 agtaatagtaacagcagcgatagtgacagcagcaacagcagcgatagcagtgacagcagcaacagcagtgacagcagtgatagcagtgacagcagcaacagcagtgacagtagcgatagcagtgacagcagcaacagcagtgacagcagtgatagcagtgacagcagtgatagtagtgacagcagcaacagcagtgatag

SHap106 agtaatagtaacagcagcgatagtgacagcagcaacagcagcgatagcagtgacagcagcaacagcagtgacagcagtgatagcagtgacagcagcaacagcagtgacagtagcgatagcagtgacagcagcaacagcagtgacagcagtgatagcagtgacagcagtgatagtagtgacagcagcaacagcagtgatag

SHap72 agtaatagtaacagcagcgatagtgacagcagcaacagcagcgatagcagtgacagcagcaacagcagtgacagcagtgatagcagtgacagcagcaacagcagtgacagtagcgatagcagtgacagcagcaacagcagtgacagcagtgatagcagtgacagcagtgatagtagtgacagcagcaacagcagtgatag

SHap110 agtaatagtaacagcagcgatagtgacagcagcaacagcagcgatagcagtgacagcagcaacagcagtgacagcagtgatagcagtgacagcagcaacagcagtgacagtagcgatagcagtgacagcagcaacagcagtgacagcagtgatagcagtgacagcagtgatagtagtgacagcagcaacagcagtgatag

Merged agtaatagtaacagcagcgatagtgacagcagcaacagcagcgatagcagtgacagcagcaacagcagtgacagcagtgatagcagtgacagcagcaacagcagtgacagtagcgatagcagtgacagcagcaacagcagtgacagcagtgatagcagtgacagcagtgatagtagtgacagcagcaacagcagtgatag

Transl S N S N S S D S D S S N S S D S S D S S N S S D S S D S S D S S N S S D S S D S S D S S N S S D S S D S S D S S D S S D S S N S S D S 785

**Note:** Dentinogenesis imperfecta type II: c.2272delA; p.Ser758Alafs*556 (2).

Dentinogenesis imperfecta type II: c.2349delT; p.Ser783Argfs*531 (5) Kindred 9.

***ID3:*** NM_014208.3:c.2214_2231delCAACAGCAGTGACAGCAG.

***ID4:*** NM_014208.3:c.2286_2303delCAACAGCAGTGACAGCAG.

2355 ***ID5 ID6*** 2525 2554

RefSeq caacgacagcagcaatagcagtgacagcagtgatagcagcaacagcagtgatagcagcaacagcagtgatagcagtgatagcagtgacagcagtgatagcgacagcagcaatagcagtgacagcagtaatagtagtgacagcagcgatagcagcaacagcagtgatagca**g**cgacagcagcgatagcagtgacggcagtg

McKnight caacgacagcagcaatagcagtgacagcagtgatagcagcaacagcagtgatagcagcaacagcagtgatagcagtgatagcagtgacagcagtgatagcgacagcagcaatagcagtgacagcagtaatagtagtgacagcagcgatagca---acagcagtgatagcagcgacagcagcgatagcagtgac**a**gcagtg

F1a CAACGACAGCAGCAATAGCAGTGACAGCAGTGATAGCAGCAACAGCAGTGATAGCAGCAACAGCAGTGATAGCAGTGATAGCAGTGACAGCAGTGATAGCGACAGCAGCAATAGCAGTGACAGCAGTAATAGTAGTGACAGCAGCGATAGCA---ACAGCAGTGATAGCAGCGACAGCAGCGATAGCAGTGAC**A**GCAGTG

F1b CAACGACAGCAGCAATAGCAGTGACAGCAGTGATAGCAGCAACAGCAGTGATAGCAGCAACAGCAGTGATAGCAGTGATAGCAGTGACAGCAGTGATAGCGACAGCAGCAATAGCAGTGACAGCAGTAATAGTAGTGACAGCAGCGATAGCAGCAACAGCAGTGATAGCAGCGACAGCAGCGATAGCAGTGAC**A**GCAGTG

F2a CAACGACAGCAGCAATAGCAGTGACAGCAGTGATAGCAGCAACAGCAGTGATAGCAGCAACAGCAGTGATAGCAGTGATAGCAGTGACAGCAGTGATAGCGACAGCAGCAATAGCAGTGACAGCAGTAATAGTAGTGACAGCAGCGATAGCAGCAACAGCAGTGATAGCAGCGACAGCAGCGATAGCAGTGAC**A**GCAGTG

F2b CAACGACAGCAGCAATAGCAGTGACAGCAGTGATAGCAGCAACAGCAGTGATAGCAGCAACAGCAGTGATAGCAGTGATAGCAGTGACAGCAGTGATAGCGACAGCAGCAATAGCAGTGACAGCAGTAATAGTAGTGACAGCAGCGATAGCAGCAACAGCAGTGATAGCAGCGACAGCAGCGATAGCAGTGACGGCAGTG

F3a CAACGACAGCAGCAATAGCAGTGACAGCAGTGATAGCAGCAACAGCAGTGATAGCAGCAACAGCAGTGATAGCAGTGATAGCAGTGACAGCAGTGATAGCGACAGCAGCAATAGCAGTGACAGCAGTAATAGTAGTGACAGCAGCGATAGCAGCAACAGCAGTGATAGCAGCGACAGCAGCGATAGCAGTGAC**A**GCAGTG

F3b CAACGACAGCAGCAATAGCAGTGACAGCAGTGATAGCAGCAACAGCAGTGATAGCAGCAACAGCAGTGATAGCAGTGATAGCAGTGACAGCAGTGATAGCGACAGCAGCAATAGCAGTGACAGCAGTAATAGTAGTGACAGCAGCGATAGCAGCAACAGCAGTGATAGCAGCGACAGCAGCGATAGCAGTGACGGCAGTG

F4a CAACGACAGCAGCAATAGCAGTGACAGCAGTGATAGCAGCAACAGCAGTGATAGCAGCAACAGCAGTGATAGCAGTGATAGCAGTGACAGCAGTGATAGCGACAGCAGCAATAGCAGTGACAGCAGTAATAGTAGTGACAGCAGCGATAGCAGC------------------GACAGCAG**A**GATAGCAGTGAC**A**GCAGTG

F4b CAACGACAGCAGCAATAGCAGTGACAGCAGTGATAGCAGCAACAGCAGTGATAGCAGCAACAGCAGTGATAGCAGTGATAGCAGTGACAGCAGTGATAGCGACAGCAGCAATAGCAGTGACAGCAGTAATAGTAGTGACAGCAGCGATAGCAGCAACAGCAGTGATAGCAGCGACAGCAGCGATAGCAGTGACGGCAGTG

F5a CAACGACAGCAGCAATAGCAGTGACAGCAGTGATAGCAGCAACAGCAGTGATAGCAGCAACAGCAGTGATAGCAGTGATAGCAGTGACAGCAGTGATAGCGACAGCAGCAATAGCAGTGACAGCAGTAATAGTAGTGACAGCAGCGATAGCAGCAACAGCAGTGATAGCAGCGACAGCAGCGATAGCAGTGACGGCAGTG

F5b CAACGACAGCAGCAATAGCAGTGACAGCAGTGATAGCAGCAACAGCAGTGATAGCAGCAACAGCAGTGATAGCAGTGATAGCAGTGACAGCAGTGATAGCGACAGCAGCAATAGCAGTGACAGCAGTAATAGTAGTGACAGCAGCGATAGCAGCAACAGCAGTGATAGCAGCGACAGCAGCGATAGCAGTGAC**A**GCAGTG

Hap1a CAACGACAGCAGCAATAGCAGTGACAGCAGTGATAGCAGCAACAGCAGTGATAGCAGCAACAGCAGTGATAGCAGTGATAGCAGTGACAGCAGTGATAGCGACAGCAGCAATAGCAGTGACAGCAGTAATAGTAGTGACAGCAGCGATAGCAGCAACAGCAGTGATAGCAGCGACAGCAGCGATAGCAGTGAC**A**GCAGTG

Hap2a CAACGACAGCAGCAATAGCAGTGACAGCAGTGATAGCAGCAACAGCAGTGATAGCAGCAACAGCAGTGATAGCAGTGATAGCAGTGACAGCAGTGATAGCGACAGCAGCAATAGCAGTGACAGCAGTAATAGTAGTGACAGCAGCGATAGCAGCAACAGCAGTGATAGCAGCGACAGCAGCGATAGCAGTGACGGCAGTG

Hap3a CAACGACAGCAGCAATAGCAGTGACAGCAGTGATAGCAGCAACAGCAGTGATAGCAGCAACAGCAGTGATAGCAGTGATAGCAGTGACAGCAGTGATAGCGACAGCAGCAATAGCAGTGACAGCAGTAATAGTAGTGACAGCAGCGATAGCAGCAACAGCAGTGATAGCAGCGACAGCAGCGATAGCAGTGACGGCAGTG

Hap15a CAACGACAGCAGCAATAGCAGTGACAGCAGTGATAGCAGCAACAGCAGTGATAGCAGCAACAGCAGTGATAGCAGTGATAGCAGTGACAGCAGTGATAGCGACAGCAGCAATAGCAGTGACAGCAGTAATAGTAGTGACAGCAGCGATAGCAGCAACAGCAGTGATAGCAGCGACAGCAGCGATAGCAGTGAC**A**GCAGTG

Hap17b caacgacagcagcaatagcagtgacagcagtgatagcagcaacagcagtgatagcagcaacagcagtgatagcagtgatagcagtgacagcagtgatagcgacagcagcaatagcagtgacagcagtaatagtagtgacagcagcgatagca---acagcagtgatagcagcgacagcagcgatagcagtgacagcagtg

Hap20a CAACGACAGCAGCAATAGCAGTGACAGCAGTGATAGCAGCAACAGCAGTGATAGCAGCAACAGCAGTGATAGCAGTGATAGCAGTGACAGCAGTGATAGCGACAGCAGCAATAGCAGTGACAGCAGTAATAGTAGTGACAGCAGCGATAGCAGCAACAGCAGTGATAGCAGCGACAGCAGCGATAGCAGTGACGGCAGTG

Hap20b caacgacagcagcaatagcagtgacagcagtgatagcagcaacagcagtgatagcagcaacagcagtgatagcagtgatagcagtgacagcagtgatagcgacagcagcaatagcagtgacagcagtaatagtagtgacagcagcgatagcagcaacagcagtgatagcagcgacagcagcgatagcagtgacggcagtg

Hap36a CAACGACAGCAGCAATAGCAGTGACAGCAGTGATAGCAGCAACAGCAGTGATAGCAGCAACAGCAGTGATAGCAGTGATAGCAGTGACAGCAGTGATAGCGACAGCAGCAATAGCAGTGACAGCAGTAATAGTAGTGACAGCAGCGATAGCAGCAACAGCAGTGATAGCAGCGACAGCAGCGATAGCAGTGACGGCAGTG

Hap37a CAACGACAGCAGCAATAGCAGTGACAGCAGTGATAGCAGCAACAGCAGTGATAGCAGCAACAGCAGTGATAGCAGTGATAGCAGTGACAGCAGTGATAGCGACAGCAGCAATAGCAGTGACAGCAGTAATAGTAGTGACAGCAGCGATAGCAGCAACAGCAGTGATAGCAGCGACAGCAGCGATAGCAGTGAC**A**GCAGTG

Hap38a CAACGACAGCAGCAATAGCAGTGACAGCAGTGATAGCAGCAACAGCAGTGATAGCAGCAACAGCAGTGATAGCAGTGATAGCAGTGACAGCAGTGATAGCGACAGCAGCAATAGCAGTGACAGCAGTAATAGTAGTGACAGCAGCGATAGCAGCAACAGCAGTGATAGCAGCGACAGCAGCGATAGCAGTGACGGCAGTG

SHap1: caacgacagcagcaatagcagtgacagcagtgatagcagcaacagcagtgatagcagcaacagcagtgatagcagtgatagcagtgacagcagtgatagcgacagcagcaatagcagtgacagcagtaatagtagtgacagcagcgatagcagcaacagcagtgatagcagcgacagcagcgatagcagtgac**a**gcagtg

SHap2: caacgacagcagcaatagcagtgacagcagtgatagcagcaacagcagtgatagcagcaacagcagtgatagcagtgatagcagtgacagcagtgatagcgacagcagcaatagcagtgacagcagtaatagtagtgacagcagcgatagcagcaacagcagtgatagcagcgacagcagcgatagcagtgacggcagtg

SHap3: caacgacagcagcaatagcagtgacagcagtgatagcagcaacagcagtgatagcagcaacagcagtgatagcagtgatagcagtgacagcagtgatagcgacagcagcaatagcagtgacagcagtaatagtagtgacagcagcgatagcagcaacagcagtgatagcagcgacagcagcgatagcagtgac**a**gcagtg

SHap4: caacgacagcagcaatagcagtgacagcagtgatagcagcaacagcagtgatagcagcaacagcagtgatagcagtgatagcagtgacagcagtgatagcgacagcagcaatagcagtgacagcagtaatagtagtgacagcagcgatagcagcaacagcagtgatagcagcgacagcagcgatagcagtgacggcagtg

SHap5: caacgacagcagcaatagcagtgacagcagtgatagcagcaacagcagtgatagcagcaacagcagtgatagcagtgatagcagtgacagcagtgatagcgacagcagcaatagcagtgacagcagtaatagtagtgacagcagcgatagca---acagcagtgatagcagcgacagcagcgatagcagtgac**a**gcagtg

SHap6: caacgacagcagcaatagcagtgacagcagtgatagcagcaacagcagtgatagcagcaacagcagtgatagcagtgatagcagtgacagcagtgatagcgacagcagcaatagcagtgacagcagtaatagtagtgacagcagcgatagcagcaacagcagtgatagcagcgacagcagcgatagcagtgac**a**gcagtg

SHap6(2) caacgacagcagcaatagcagtgacagcagtgatagcagcaacagcagtgatagcagcaacagcagtgatagcagtgatagcagtgacagcagtgatagcgacagcagcaatagcagtgacagcagtaatagtagtgacagcagcgatagca---acagcagtgatagcagcgacagcagcgatagcagtgac**a**gcagtg

SHap7 caacgacagcagcaatagcagtgacagcagtgatagcagcaacagcagtgatagcagcaacagcagtgatagcagtgatagcagtgacagcagtgatagcgacagcagcaatagcagtgacagcagtaatagtagtgacagcagcgatagcagcaacagcagtgatagcagcgacagcagcgatagcagtgacggcagtg

SHap102 caacgacagcagcaatagcagtgacagcagtgatagcagcaacagcagtgatagcagcaacagcagtgatagcagtgatagcagtgacagcagtgatagcgacagcagcaatagcagtgacagcagtaatagtagtgacagcagcgatagcagcaacagcagtgatagcagcgacagcagcgatagcagtgac**a**gcagtg

SHap130 caacgacagcagcaatagcagtgacagcagtgatagcagcaacagcagtgatagcagcaacagcagtgatagcagtgatagcagtgacagcagtgatagcgacagcagcaatagcagtgacagcagtaatagtagtgacagcagcgatagcagcaacagcagtgatagcagcgacagcagcgatagcagtgac**a**gcagtg

SHap106 caacgacagcagcaatagcagtgacagcagtgatagcagcaacagcagtgatagcagcaacagcagtgatagcagtgatagcagtgacagcagtgatagcgacagcagcaatagcagtgacagcagtaatagtagtgacagcagcgatagcagcaacagcagtgatagcagcgacagcagcgatagcagtgac**a**gcagtg

SHap72 caacgacagcagcaatagcagtgacagcagtgatagcagcaacagcagtgatagcagcaacagcagtgatagcagtgatagcagtgacagcagtgatagcgacagcagcaatagcagtgacagcagtaatagtagtgacagcagcgatagcagcaacagcagtgatagcagcgacagcagcgatagcagtgac**a**gcagtg

SHap110 caacgacagcagcaatagcagtgacagcagtgatagcagcaacagcagtgatagcagcaacagcagtgatagcagtgatagcagtgacagcagtgatagcgacagcagcaatagcagtgacagcagtaatagtagtgacagcagcgatagcagcaacagcagtgatagcagcgacagcagcgatagcagtgacggcagtg

Merged caacgacagcagcaatagcagtgacagcagtgatagcagcaacagcagtgatagcagcaacagcagtgatagcagtgatagcagtgacagcagtgatagcgacagcagcaatagcagtgacagcagtaatagtagtgacagcagcgatagcagcaacagcagtgatagcagcgacagcagcgatagcagtgacggcagtg

Transl N D S S N S S D S S D S S N S S D S S N S S D S S D S S D S S D S D S S N S S D S S N S S D S S D S S N S S D S S D S S/**R** D S S D G/**S** S 851

**Notes:** Dentinogenesis imperfecta type II mutation c.2525delG; p.Ser842Thrfs*472 (2).

SNPs: c.2535C>A or p.Ser845Arg; c.2535C>A; c.2548G>A (p.Gly850Ser).

***ID5:*** NM_014208.3:c.2507_2509delGCA.

***ID6:*** NM_014208.3:c.2509_2526delAACAGCAGTGATAGCAGC.

2555 ***ID7*** 2593 ***ID8*** 2666 2684 2688 ***ID9*** 2745

RefSeq atagcgacagcagcaatagaagtgacagtagtaatagt**a**gtgacagcagcgatagcagtgacagcagcaacagcagtgacagcagtgatag**---------**cagtgacagcaacgaaagca**g**caatagcagtgacagca**g**tga**t**agcagcaacagcagtgatagtgacagcagtgatagcagcaacagcagtgacagcagt

McKnight atagcgacagcagcaatagaagtgacagtagtaatagtagtgacagcagcgatagcagtgacagcagcaacagcagtgacagcagtgatagtagtgacagcagtgacagcaacgaaagcagcaatagcagtgacagcagtgatagcagcaacagcagtgatagtgacagcagtgatagcagcaacagcagtgacagcagt

F1a ATAGCGACAGCAGCAATAGAAGTGACAGTAGTAATAGTAGTGACAGCAGCGATAGCAGTGACAGCAGCAACAGCAGTGACAGCAGTGATAGTAGTGACAGCAGTGACAGCAACGAAAGCAGCAATAGCAGTGACAGCAGTGATAGCAGCAACAGCAGTGATAGTGACAGCAGTGATAGCAGCAACAGCAGTGACAGCAGT

F1b ATAGCGACAGCAGCAATAGAAGTGACAGTAGTAATAGTAGTGACAGCAGCGATAGCAGTGACAGCAGCAACAGCAGTGACAGCAGTGATAGTAGTGACAGCAGTGACAGCAACGAAAGCAGCAATAGCAGTGACAGCAGTGATAGCAGCAACAGCAGTGATAGTGACAGCAGTGATAGCAGCAACAGCAGTGACAGCAGT

F2a ATAGCGACAGCAGCAATAGAAGTGACAGTAGTAATAGTAGTGACAGCAGCGATAGCAGTGACAGCAGCAACAGCAGTGACAGCAGTGATAGTAGTGACAGCAGTGACAGCAACGAAAGCAGCAATAGCAGTGACAGCAGTGATAGCAGCAACAGCAGTGATAGTGACAGCAGTGATAGCAGCAACAGCAG**C**GACAGCAGT

F2b ATAGCGACAGCAGCAATAGAAGTGACAGTAGTAATAGTAGTGACAGCAGCGATAGCAGTGACAGCAGCAACAGCAGTGACAGCAGTGATAG---------CAGTGACAGCAACGAAAGCAGCAATAGCAGTGACAGCAGTGATAGCAGCAACAGCAGTGACAGTGACAGCAGTGATAGCAGCAACAGCAGTGACAGCAGT

F3a ATAGCGACAGCAGCAATAGAAGTGACAGTAGTAATAGTAGTGACAGCAGCGATAGCAGTGACAGCAGCAACAGCAGTGACAGCAGTGATAGTAGTGACAGCAGTGACAGCAACGAAAGCAGCAATAGCAGTGACAGCAGTGATAGCAGCAACAGCAGTGATAGTGACAGCAGTGATAGCAGCAACAGCAGCGACAGCAGT

F3b ATAGCGACAGCAGCAATAGAAGTGACAGTAGTAATAGTAGTGACAGCAGCGATAGCAGTGACAGCAGCAACAGCAGTGACAGCAGTGATAG---------CAGTGACAGCAACGAAAGCAGCAATAGCAGTGACAGCAGTGATAGCAGCAACAGCAGTGACAGTGACAGCAGTGATAGCAGCAACAGCAGTGACAGCAGT

F4a ATAGCGACAGCAGCAATAGAAGTGACAGTAGTAATAGTAGTGACAGCAGCGATAGCAGTGACAGCAGCAACAGCAGTGACAGCAGTGATAGTAGTGACAGCAGTGACAGCAACGAAAGCAGCAATAGCAGTGACAGCAGTGATAGCAGCAACAGCAGTGATAGTGACAGCAGTGATAGCAGCAACAGCAGTGACAGCAGT

F4b ATAGCGACAGCAGCAATAGAAGTGACAGTAGTAATAGTAGTGACAGCAGCGATAGCAGTGACAGCAGCAACAGCAGTGACAGCAGTGATAG---------CAGTGACAGCAACGAAAGCAGCAATAGCAGTGACAGCAGTGATAGCAGCAACAGCAGTGACAGTGACAGCAGTGATAGCAGCAACAGCAGTGACAGCAGT

F5a ATAGCGACAGCAGCAATAGAAGTGACAGTAGTAATAGTAGTGACAGCAGCGATAGCAGTGACAGCAGCAACAGCAGTGACAGCAGTGATAG---------CAGTGACAGCAACGAAAGCAGCAATAGCAGTGACAGCAGTGATAGCAGCAACAGCAGTGACAGTGACAGCAGTGATAGCAGCAACAGCAGTGACAGCAGT

F5b ATAGCGACAGCAGCAATAGAAGTGACAGTAGTAATAGTAGTGACAGCAGCGATAGCAGTGACAGCAGCAACAGCAGTGACAGCAGTGATAGTAGTGACAGCAGTGACAGCAACGAAAGCAGCAATAGCAGTGACAGCAGTGATAGCAGCAACAGCAGTGATAGTGACAGCAGTGATAGCAGCAACAGCAGCGACAGCAGT

Hap1a ATAGCGACAGCAGCAATAGAAGTGACAGTAGTAATAGTAGTGACAGCAGCGATAGCAGTGACAGCAGCAACAGCAGTGACAGCAGTGATAGTAGTGACAGCAGTGACAGCAACGAAAGCAGCAATAGCAGTGACAGCAGTGATAGCAGCAACAGCAGTGATAGTGACAGCAGTGATAGCAGCAACAGCAGTGACAGCAGT

Hap2a ATAGCGACAGCAGCAATAGAAGTGACAGTAGTAATAGTAGTGACAGCAGCGATAGCAGTGACAGCAGCAACAGCAGTGACAGCAGTGATAG---------CAGTGACAGCAACGAAAGCAGCAATAGCAGTGACAGCAGTGATAGCAGCAACAGCAGTGACAGTGACAGCAGTGATAGCAGCAACAGCAGTGACAGCAGT

Hap3a ATAGCGACAGCAGCAATAGAAGTGACAGTAGTAATAGTAGTGACAGCAGCGATAGCAGTGACAGCAGCAACAGCAGTGACAGCAGTGATAG---------CAGTGACAGCAACGAAAGCAGCAATAGCAGTGACAGCAGTGATAGCAGCAACAGCAGTGACAGTGACAGCAGTGATAGCAGCAACAGCAGTGACAGCAGT

Hap15a ATAGCGACAGCAGCAATAGAAGTGACAGTAGTAATAGTAGTGACAGCAGCGATAGCAGTGACAGCAGCAACAGCAGTGACAGCAGTGATAGTAGTGACAGCAGTGACAGCAACGAAAGCAGCAATAGCAGTGACAGCAGTGATAGCAGCAACAGCAGTGATAGTGACAGCAGTGATAGCAGCAACAGCAG**C**GACAGCAGT

Hap17b atagcgacagcagcaatagaagtgacagtagtaatagtagtgacagcagcgatagcagtgacagcagcaacagcagtgacagcagtgatagtagtgacagcagtgacagcaacgaaagcagcaatagcagtgacagcagtgatagcagcaacagcagtgatagtgacagcagtgatagcagcaacagcagcgacagcagt

Hap20a ATAGCGACAGCAGCAATAGAAGTGACAGTAGTAATAGTAGTGACAGCAGCGATAGCAGTGACAGCAGCAACAGCAGTGACAGCAGTGATAGTAGTGACAGCAGTGACAGCAACGAAAGCAGCAATAGCAGTGACAGCAGTGATAGCAGCAACAGCAGTGATAGTGACAGCAGTGATAGCAGCAACAGCAGT---------

Hap20b atagcgacagcagcaatagaagtgacagtagtaatagtagtgacagcagcgatagcagtgacagcagcaacagcagtgacagcagtgatagtagtgacagcagtgacagcaacgaaagcagcaatagcagtgacagcagtgatagcagcaacagcagtgatagtgacagcagtgatagcagcaacagcagtgacagcagt

Hap36a ATAGCGACAGCAGCAATAGAAGTGACAGTAGTAATAGTAGTGACAGCAGCGATAGCAGTGACAGCAGCAACAGCAGTGACAGCAGTGATAGTAGTGACAGCAGTGACAGCAACGAAAGCAGCAATAGCAGTGACAGCAGTGATAGCAGCAACAGCAGTGATAGTGACAGCAGTGATAGCAGCAACAGCAGT---------

Hap37a ATAGCGACAGCAGC------------------------------------GATAGCAGTGACAGCAGCAACAGCAGTGACAGCAGTGATAGTAGTGACAGCAGTGACAGCAACGAAAGCAGCAATAGCAGTGACAGCAGTGATAGCAGCAACAGCAGTGATAGTGACAGCAGTGATAGCAGCAACAGCAGTGACAGCAGT

Hap38a ATAGC**A**ACAGCAGCAATAGAAGTGACAGTAGTAATAGTAGTGACAGCAGCGATAGCAGTGACAGCAGCAACAGCAGTGACAGCAGTGATAGTAGTGACAGCAGTGACAGCAACGAAAGCAGCAATAGCAGTGACAGCAGTGATAGCAGCAACAGCAGTGATAGTGACAGCAGTGATAGCAGCAACAGCAGT---------

SHap1: atagcgacagcagcaatagaagtgacagtagtaatagtagtgacagcagcgatagcagtgacagcagcaacagcagtgacagcagtgatagtagtgacagcagtgacagcaacgaaagcagcaatagcagtgacagcagtgatagcagcaacagcagtgatagtgacagcagtgatagcagcaacagcagtgacagcagt

SHap2: atagcgacagcagcaatagaagtgacagtagtaatagtagtgacagcagcgatagcagtgacagcagcaacagcagtgacagcagtgatag**---------**cagtgacagcaacgaaagcagcaatagcagtgacagcagtgatagcagcaacagcagtgatagtgacagcagtgatagcagcaacagcagtgacagcagt

SHap3: atagcgacagcagcaatagaagtgacagtagtaatagtagtgacagcagcgatagcagtgacagcagcaacagcagtgacagcagtgatagtagtgacagcagtgacagcaacgaaagcagcaatagcagtgacagcagtgatagcagcaacagcagtgatagtgacagcagtgatagcagcaacagcagtgacagcagt

SHap4: atagcgacagcagcaatagaagtgacagtagtaatagtagtgacagcagcgatagcagtgacagcagcaacagcagtgacagcagtgatag**---------**cagtgacagcaacgaaagcagcaatagcagtgacagcagtgatagcagcaacagcagtgatagtgacagcagtgatagcagcaacagcagtgacagcagt

SHap5: atagcgacagcagcaatagaagtgacagtagtaatagtagtgacagcagcgatagcagtgacagcagcaacagcagtgacagcagtgatagtagtgacagcagtgacagcaacgaaagcagcaatagcagtgacagcagtgatagcagcaacagcagtgatagtgacagcagtgatagcagcaacagcagtgacagcagt

SHap6: atagcgacagcagcaatagaag**g**gacagtagtaatagtagtgacagcagcgatagcagtgacagcagcaacagcagtgacagcagtgatagtagtgacagcagtgacagcaacgaaagcagcaatagcagtgacagcagtgatagcagcaacagcagtgatagtgacagcagtgatagcagcaacagcagtgacagcagt

SHap6(2) atagcgacagcagcaatagaagtgacagtagtaatagtagtgacagcagcgatagcagtgacagcagcaacagcagtgacagcagtgatagtagtgacagcagtgacagcaacgaaagcagcaatagcagtgacagcagtgatagcagcaacagcagtgatagtgacagcagtgatagcagcaacagcagtgacagcagt

SHap7 atagcgacagcagcaatagaagtgacagtagtaatagtagtgacagcagcgatagcagtgacagcagcaacagcagtgacagcagtgatag**---------**cagtgacagcaacgaaagcagcaatagcagtgacagcagtgatagcagcaacagcagtgatagtgacagcagtgatagcagcaacagcagtgacagcagt

SHap102 atagcgacagcagcaatagaagtgacagtagtaatagtagtgacagcagcgatagcagtgacagcagcaacagcagtgacagcagtgatagtagtgacagcagtgacagcaacgaaagcagcaatagcagtgacagcagtgatagcagcaacagcagtgatagtgacagcagtgatagcagcaacagcagtgacagcagt

SHap130 atagcgacagcagcaatagaagtgacagtagtaatagtagtgacagcagcgatagcagtgacagcagcaacagcagtgacagcagtgatagtagtgacagcagtgacagcaacgaaagcagcaatagcagtgacagcagtgatagcagcaacagcagtgatagtgacagcagtgatagcagcaacagcagtgacagcagt

SHap106 atagcgacagcagcaatagaagtgacagtagtaatagtagtgacagcagcgatagcagtgacagcagcaacagcagtgacagcagtgatagtagtgacagcagtgacagcaacgaaagcagcaatagcagtgacagcagtgatagcagcaacagcagtgatagtgacagcagtgatagcagcaacagcagtgacagcagt

SHap72 atagcgacagcagcaatagaagtgacagtagtaatagtagtgacagcagcgatagcagtgacagcagcaacagcagtgacagcagtgatagtagtgacagcagtgacagcaacgaaagcagcaatagcagtgacagcagtgatagcagcaacagcagtgatagtgacagcagtgatagcagcaacagcagtgacagcagt

SHap110 atagcgacagcagcaatagaagtgacagtagtaatagtagtgacagcagcgatagcagtgacagcagcaacagcagtgacagcagtgatag**---------**cagtgacagcaacgaaagcagcaatagcagtgacagcagtgatagcagcaacagcagtgatagtgacagcagtgatagcagcaacagcagtgacagcagt

Hap4 agaagtgacagtagtaatagtagtgacagcagcgatagcagtgacagcagcaacagcagtgacagcagtgatagtagtgacagcagtgacagcaacgaaagcagcaatagcagtgacagcagtgatagcagcaacagcagtgatagtgacagcagtgatagcagcaacagcag**c**gacagcagt

Hap5 agaagtgacagtagtaatagtagtgacagcagcgatagcagtgacagcagcaacagcagtgacagcagtgatagtagtgacagcagtgacagcaacgaaagcagcaatagcagtgacagcagtgatagcagcaacagcagtgatagtgacagcagtgatagcagcaacagcagt---------

Hap8 agaag**g**gacagtagtaatagtagtgacagcagcgatagcagtgacagcagcaacagcagtgacagcagtgatagtagtgacagcagtgacagcaacgaaagcagcaatagcagtgacagcagtgatagcagcaacagcagtgatagtgacagcagtgatagcagcaacagcagtgacagcagt

Hap13 agaagtgacagtagtaatagtagtgacagcagcgatagcagtgacagcagcaacagcagtgacagcagtgatagtagtgacagcagtgacagcaacgaaagcagcaatagcagtgacagcagtgatagcagcaacagcagtgatagtgacagcagtgatagcagcaacagcag**c**gacagcagt

Hap24 agaagtgacagtagtaatagtagtgacagcagcgatagcagtgacagcagcaacagcagtgacagcagtgatag**---------**cagtgacagcaacgaaagcagcaatagcagtgacagcagtgatagcagcaacagcagtga**c**agtgacagcagtgatagcagcaacagcagtgacagcagt

Hap25 agaagtgacagtagtaatagtagtgacagcagcgatagcagtgacagcagcaacagcagtgacagcagtgatagtagtgacagcagtgacagcaacgaaagcagcaatagcagtgacagcagtgatagcagcaacagcagtgatagtgacagcagtgatagcagcaacagcagtgacagcagt

Hap26 agaagtgacagtagtaatagtagtgacagcagcgatagcagtgacagcagcaacagcagtgacagcagtgatagtagtgacagcagtgacagcaacgaaagcagcaatagcagtgacagcagtgatagcagcaacagcagtgatagtgacagcagtgatagcagcaacagcagtgacagcagt

Hap29 agaagtgacagtagtaatagtagtgacagcagcgatagcagtgacagcagcaacagcagtgacagcagtgatagtagtgacagcagtgacagcaacgaaagcagcaatagcagtgacagcagtgatagcagcaacagcagtgatagtgacagcagtgatagcagcaacagcagtgacagcagt

Hap32 agaagtgacagtagtaatagtagtgacagcagcgatagcagtgacagcagcaacagcagtgacagcagtgatagtagtgacagcagtgacagcaacgaaagcagcaatagcagtgacagcagtgatagcagcaacagcagtgatagtgacagcagtgatagcagcaacagcagt---------

Hap33 agaagtgacagtagtaatagtagtgacagcagcgatagcagtgacagcagcaacagcagtgacagcagtgatagtagtgacagcagtgacagcaacgaaagcagcaatagcagtgacagcagtgatagcagcaacagcagtgatagtgacagcagtgatagcagcaacagcagtgacagcagt

Hap35 agaagtgacagtagtaatagtagtgacagcagcgatagcagtgacagcagcaacagcagtgacagcagtgatagtagtgacagcagtgacagcaacgaaagcagcaatagcagtgacagcagtgatagcagcaacagcagtgatagtgacagcagtgatagcagcaacagcagtgacagcagt

Merged atagcgacagcagcaatagaagtgacagtagtaatagtagtgacagcagcgatagcagtgacagcagcaacagcagtgacagcagtgatag**tagtgacag**cagtgacagcaacgaaagcagcaatagcagtgacagcagtgatagcagcaacagcagtgatagtgacagcagtgatagcagcaacagcagtgacagcagt

Transl D S D/**N** S S N R S/**R** D S S N S S D S S D S S D S S N S S D S S D S **S D S** S D S N E S S N S S D S S D S S N S S D S D S S D S S N S S D S S 915

**Notes:** c.2571T is the beginning of the Hap3 to Hap38 sequences.

Dentinogenesis imperfecta type II: c.2593delA; p.Ser865Valfs*449 (4) Family J.

Dentinogenesis imperfecta type II: c.2666delG; p.Ser889Thrfs*425 (5) Kindred 10.

Dentinogenesis imperfecta type II: c.2684delG; p.Ser895Metfs*419 (4) Family S.

Dentinogenesis imperfecta type II: c.2688delT; p.Asp896Glufs*418 (6).

SNPs: c.2560G>A (p.Asp854Asn); c.2577T>G (p.Ser859Arg); c.2706T>C; c.2736T>C.

***ID7:*** NM_014208.3:c.2569_2604delAATAGAAGTGACAGTAGTAATAGTAGTGACAGCAGC.

***ID8:*** NM_014208.3:c.2645_2646insTAGTGACAG.

***ID9:*** NM_014208.3:c.2737_2745delGACAGCAGT.

2746 ***ID10 ID11*** 2927

RefSeq gatagcagcaacagcagtgatagcagtgaaagcagtaatagtagtgacaacagcaatagcagtgacagcagcaacagcagtgacagcagtgatagcagtgacagcagtaatagtagtgacagcagcaatagcagtgacagcagcaacagcagtgacagcag------------------tgatagcaatagcagcgacag

McKnight gatagcagcaacagcagtgatagcagtgaaagcagtaatagtagtgacaacagcaatagcagtgacagcagcaacagcagtgacagcagtgatagcagtgacagcagtaatagtagtgacagcagcaatagc**g**gtgacagcagcaacagcagtgacagcag------------------tgatagcaatagcagcgacag

F1a GATAGCAGCAACAGCAGTGATAGCAGTGAAAGCAGTAATAGTAGTGACAACAGCAATAGCAGTGACAGCAGCAACAGCAGTGACAGCAGTGATAGCAGTGACAGCAGTAATAGTAGTGACAGCAGCAATAGC**G**GTGACAGCAGCAACAGCAGTGACAGCAG------------------TGATAGCAATAGCAGCGACAG

F1b GATAGCAGCAACAGCAGTGATAGCAGTGAAAGCAGTAATAGTAGTGACAACAGCAATAGCAGTGACAGCAGCAACAGCAGTGACAGCAGTGATAGCAGTGACAGCAGTAATAGTAGTGACAGCAGCAATAGC**G**GTGACAGCAGCAACAGCAGTGACAGCAG------------------TGATAGCAATAGCAGCGACAG

F2a GATAGCAGCAACAGCAGTGATAGCAGTGAAAGCAGTAATAGTAGTGACAACAGCAATAGCAGTGACAGCAGCAACAGCAGTGACAGCAGTGATAGCAGTGACAGCAGTAATAGTAGTGACAGCAGCAATAGC**G**GTGACAGCAGCAACAGCAGTGACAGCAG------------------TGATAGCAATAGCAGCGACAG

F2b GATAGCAGCAACAGCAGTGATAGCAGTGAAAGCAGTAATAGTAGTGACAACAGCAATAGCAGTGACAGCAGCAACAGCAGTGACAGCAGTGATAGCAGTGACAGCAGTAATAGTAGTGACAGCAGCAATAGCAGTGACAGCAGCAACAGCAGTGACAGCAGCAACAGCAGTGACAGCAGTGATAGCAATAGCAGCGACAG

F3a GATAGCAGCAACAGCAGTGATAGCAGTGAAAGCAGTAATAGTAGTGACAACAGCAATAGCAGTGACAGCAGCAACAGCAGTGACAGCAGTGATAGCAGTGACAGCAGTAATAGTAGTGACAGCAGCAATAGC**G**GTGACAGCAGCAACAGCAGTGACAGCAG------------------TGATAGCAATAGCAGCGACAG

F3b GATAGCAGCAACAGCAGTGATAGCAGTGAAAGCAGTAATAGTAGTGACAACAGCAATAGCAGTGACAGCAGCAACAGCAGTGACAGCAGTGATAGCAGTGACAGCAGTAATAGTAGTGACAGCAGCAATAGCAGTGACAGCAGCAACAGCAGTGACAGCAG------------------TGATAGCAATAGCAGCGACAG

F4a GATAGCAGCAACAGCAGTGATAGCAGTGAAAGCAGTAATAGTAGTGACAACAGCAATAGCAGTGACAGCAGCAACAGCAGTGACAGCAGTGATAGCAGTGACAGCAGTAATAGTAGTGACAGCAGCAATAGCAGTGACAGCAGCAACAGCAGTGACAGCAG------------------TGATAGCAATAGCAGCGACAG

F4b GATAGCAGCAACAGCAGTGATAGCAGTGAAAGCAGTAATAGTAGTGACAACAGCAATAGCAGTGACAGCAGCAACAGCAGTGACAGCAGTGATAGCAGTGACAGCAGTAATAGTAGTGACAGCAGCAATAGCAGTGACAGCAGCAACAGCAGTGACAGCAG------------------TGATAGCAATAGCAGCGACAG

F5a GATAGCAGCAACAGCAGTGATAGCAGTGAAAGCAGTAATAGTAGTGACAACAGCAATAGCAGTGACAGCAGCAACAGCAGTGACAGCAGTGATAGCAGTGACAGCAGTAATAGTAGTGACAGCAGCAATAGCAGTGACAGCAGCAACAGCAGTGACAGCAG------------------TGATAGCAATAGCAGCGACAG

F5b GATAGCAGCAACAGCAGTGATAGCAGTGAAAGCAGTAATAGTAGTGACAACAGCAATAGCAGTGACAGCAGCAACAGCAGTGACAGCAGTGATAGCAGTGACAGCAGTAATAGTAGTGACAGCAGCAATAGC**G**GTGACAGCAGCAACAGCAGTGACAGCAG------------------TGATAGCAATAGCAGCGACAG

Hap1a GATAGCAGCAACAGCAGTGATAGCAGTGAAAGCAGTAATAGTAGTGACAACAGCAATAGCAGTGACAGCAGCAACAGCAGTGACAGCAGTGATAGCAGTGACAGCAGTAATAGTAGTGACAGCAGCAATAGC**G**GTGACAGCAGCAACAGCAGTGACAGCAG------------------TGATAGCAATAGCAGCGACAG

Hap2a GATAGCAGCAACAGCAGTGATAGCAGTGAAAGCAGTAATAGTAGTGACAACAGCAATAGCAGTGACAGCAGCAACAGCAGTGACAGCAGTGATAGCAGTGACAGCAGTAATAGTAGTGACAGCAGCAATAGCAGTGACAGCAGCAACAGCAGTGACAGCAG------------------TGATAGCAATAGCAGCGACAG

Hap3a GATAGCAGCAACAGCAGTGATAGCAGTGAAAGCAGTAATAGTAGTGACAACAGCAATAGCAGTGACAGCAGCAACAGCAGTGACAGCAGTGATAGCAGTGACAGCAGTAATAGTAGTGACAGCAGCAATAGCAGTGACAGCAGCAACAGCAGTGACAGCAG------------------TGATAGCAATAGCAGCGACAG

Hap15a GATAGCAGCAACAGCAGTGATAGCAGTGAAAGCAGTAATAGTAGTGACAACAGCAATAGCAGTGACAGCAGCAACAGCAGTGACAGCAGTGATAGCAGTGACAGCAGTAATAGTAGTGACAGCAGCAATAGC**G**GTGACAGCAGCAACAGCAGTGACAGCAG------------------TGATAGCAATAGCAGCGACAG

Hap17b gatagcagcaacagcagtgatagcagtgaaagcagtaatagtagtgacaacagcaatagcagtgacagcagcaacagcagtgacagcagtgatagcagtgacagcagtaatagtagtgacagcagcaatagc**g**gtgacagcagcaacagcagtgacagcag------------------tgatagcaatagcagcgacag

Hap20a GATAGCAGCAACAGCAGTGATAGCAGTGAAAGCAGTAATAGTAGTGACAACAGCAATAGCAGTGACAGCAGCAACAGCAGTGACAGCAGTGATAGCAGTGACAGCAGTAATAGTAGTGACAGCAGCAATAGCAGTGACAGCAGCAACAGCAGTGACAGCAG------------------TGATAGCAATAGCAGCGACAG

Hap20b gatagcagcaacagcagtgatagcagtgaaagcagtaatagtagtgacaacagcaatagcagtgacagcagcaacagcagtgacagcagtgatagcagtgacagcagtaatagtagtgacagcagcaatagc**g**gtgacagcagcaacagcagtgacagcag------------------tgatagcaatagcagcgacag

Hap36a GATAGCAGCAACAGCAGTGATAGCAGTGAAAGCAGTAATAGTAGTGACAACAGCAATAGCAGTGACAGCAGCAACAGCAGTGACAGCAGTGATAGCAGTGACAGCAGTAATAGTAGTGACAGCAGCAATAGCAGTGACAGCAGCAACAGCAGTGACAGCAG------------------TGATAGCAATAGCAGCGACAG

Hap37a GATAGCAGCAACAGCAGTGATAGCAGTGAAAGCAGTAATAGTAGTGACAACAGCAATAGCAGTGACAGCAGCAACAGCAGTGACAGCAGT---------GACAGCAGTAATAGTAGTGACAGCAGCAATAGCAGTGACAGCAGCAACAGCAGTGACAGCAG------------------TGATAGCAATAGCAGCGACAG

Hap38a GATAGCAGCAACAGCAGTGATAGCAGTGAAAGCAGTAATAGTAGTGACAACAGCAATAGCAGTGACAGCAGCAACAGCAGTGACAGCAGTGATAGCAGTGACAGCAGTAATAGTAGTGACAGCAGCAATAGCAGTGACAGCAGCAACAGCAGTGACAGCAG------------------TGATAGCAATAGCAGCGACAG

SHap1: gatagcagcaacagcagtgatagcagtgaaagcagtaatagtagtgacaacagcaatagcagtgacagcagcaacagcagtgacagcagtgatagcagtgacagcagtaatagtagtgacagcagcaatagc**g**gtgacagcagcaacagcagtgacagcag------------------tgatagcaatagcagcgacag

SHap2: gatagcagcaacagcagtgatagcagtgaaagcagtaatagtagtgacaacagcaatagcagtgacagcagcaacagcagtgacagcagtgatagcagtgacagcagtaatagtagtgacagcagcaatagcagtgacagcagcaacagcagtgacagcag------------------tgatagcaatagcagcgacag

SHap3: gatagcagcaacagcagtgatagcagtgaaagcagtaatagtagtgacaacagcaatagcagtgacagcagcaacagcagtgacagcagtgatagcagtgacagcagtaatagtagtgacagcagcaatagc**g**gtgacagcagcaacagcagtgacagcag------------------tgatagcaatagcagcgacag

SHap4: gatagcagcaacagcagtgatagcagtgaaagcagtaatagtagtgacaacagcaatagcagtgacagcagcaacagcagtgacagcagtgatagcagtgacagcagtaatagtagtgacagcagcaatagcagtgacagcagcaacagcagtgacagcag------------------tgatagcaatagcagcgacag

SHap5: gatagcagcaacagcagtgatagcagtgaaagcagtaatagtagtgacaacagcaatagcagtgacagcagcaacagcagtgacagcagtgatagcagtgacagcagtaatagtagtgacagcagcaatagc**g**gtgacagcagcaacagcagtgacagcag------------------tgatagcaatagcagcgacag

SHap6: gatagcagcaacagcagtgatagcagtgaaagcagtaatagtagtgacaacagcaatagcagtgacagcagcaacagcagtgacagcagtgatagcagtgacagcagtaatagtagtgacagcagcaatagc**g**gtgacagcagcaacagcagtgacagcag------------------tgatagcaatagcagcgacag

SHap6(2) gatagcagcaacagcagtgatagcagtgaaagcagtaatagtagtgacaacagcaatagcagtgacagcagcaacagcagtgacagcagtgatagcagtgacagcagtaatagtagtgacagcagcaatagc**g**gtgacagcagcaacagcagtgacagcag------------------tgatagcaatagcagcgacag

SHap7 gatagcagcaacagcagtgatagcagtgaaagcagtaatagtagtgacaacagcaatagcagtgacagcagcaacagcagtgacagcagtgatagcagtgacagcagtaatagtagtgacagcagcaatagcagtgacagcagcaacagcagtgacagcag------------------tgatagcaatagcagcgacag

SHap102 gatagcagcaacagcagtgatagcagtgaaagcagtaatagtagtgacaacagcaatagcagtgacagcagcaacagcagtgacagcagtgatagcagtgacagcagtaatagtagtgacagcagcaatagcagtgacagcagcaacagcagtgacagcag------------------tgatagcaatagcagcgacag

SHap130 gatagcagcaacagcagtgatagcagtgaaagcagtaatagtagtgacaacagcaatagcagtgacagcagcaacagcagtgacagcagtgatagcagtgacagcagtaatagtagtgacagcagcaatagc**g**gtgacagcagcaacagcagtgacagcag------------------tgatagcaatagcagcgacag

SHap106 gatagcagcaacagcagtgatagcagtgaaagcagtaatagtagtgacaacagcaatagcagtgacagcagcaacagcagtgacagcagtgatagcagtgacagcagtaatagtagtgacagcagcaatagc**g**gtgacagcagcaacagcagtgacagcag------------------tgatagcaatagcagcgacag

SHap72 gatagcagcaacagcagtgatagcagtgaaagcagtaatagtagtgacaacagcaatagcagtgacagcagcaacagcagtgacagcagtgatagcagtgacagcagtaatagtagtgacagcagcaatagc**g**gtgacagcagcaacagcagtgacagcag------------------tgatagcaatagcagcgacag

SHap110 gatagcagcaacagcagtgatagcagtgaaagcagtaatagtagtgacaacagcaatagcagtgacagcagcaacagcagtgacagcagtgatagcagtgacagcagtaatagtagtgacagcagcaatagcagtgacagcagcaacagcagtgacagcag------------------tgatagcaatagcagcgacag

Hap4 gatagcagcaacagcagtgatagcagtgaaagcagtaatagtagtgacaacagcaatagcagtgacagcagcaacagcagtgacagcagtgatagcagtgacagcagtaatagtagtgacagcagcaatagcagtgacagcagcaacagcagtgacagcag------------------tgatagcaatagcagcgacag

Hap5 gatagcagcaacagcagtgatagcagtgaaagcagtaatagtagtgacaacagcaatagcagtgacagcagcaacagcagtgacagcagtgatagcagtgacagcagtaatagtagtgacagcagcaatagcagtgacagcagcaacagcagtgacagcag------------------tgatagcaatagcagcgacag

Hap8 gatagcagcaacagcagtgatagcagtgaaagcagtaatagtagtgacaacagcaatagcagtgacagcagcaacagcagtgacagcagtgatagcagtgacagcagtaatagtagtgacagcagcaatagc**g**gtgacagcagcaacagcagtgacagcag------------------tgatagcaatagcagcgacag

Hap13 gatagcagcaacagcagtgatagcagtgaaagcagtaatagtagtgacaacagcaatagcagtgacagcagcaacagcagtgacagcagtgatagcagtgacagcagtaatagtagtgacagcagcaatagc**g**gtgacagcagcaacagcagtgacagcag------------------tgatagcaatagcagcgacag

Hap24 gatagcagcaacagcagtgatagcagtgaaagcagtaatagtagtgacaacagcaatagcagtgacagcagcaacagcagtgacagcagtgatagcagtgacagcagtaatagtagtgacagcagcaatagcagtgacagcagcaacagcagtgacagcag------------------tgatagcaatagcagcgacag

Hap25 gatagcagcaacagcagtgatagcagtgaaagcagtaatagtagtgacaacagcaatagcagtgacagcagcaacagcagtgacagcagtgatagcagtgacagcagtaatagtagtgacagcagcaatagc**g**gtgacagcagcaacagcagtgacagcag------------------tgatagcaatagcagcgacag

Hap26 gatagcagcaacagcagtgatagcagtgaaagcagtaatagtagtgacaacagcaatagcagtgacagcagcaacagcagtgacagcagt---------gacagcagtaatagtagtgacagcagcaatagcagtgacagcagcaacagcagtgacagcag------------------tgatagcaatagcagcgacag

Hap29 gatagcagcaacagcagtgatagcagtgaaagcagtaatagtagtgacaacagcaatagcagtgacagcagcaacagcagtgacagcagtgatagcagtgacagcagtaatagtagtgacagcagcaatagcagtgacagcagcaacagcagtgacagcag------------------tgatagcaatagcagcgacag

Hap32 gatagcagcaacagcagtgatagcagtgaaagcagtaatagtagtgacaacagcaatagcagtgacagcagcaacagcagtgacagcagtgatagcagtgacagcagtaatagtagtgacagcagcaatagcagtgacagcagcaacagcagtgacagcag------------------tgatagcaatagcagcgacag

Hap33 gatagcagcaacagcagtgatagcagtgaaagcagtaatagtagtgacaacagcaatagcagtgacagcagcaacagcagtgacagcagtgatagcagtgacagcagtaatagtagtgacagcagcaatagc**g**gtgacagcagcaacagcagtgacagcag------------------tgatagcaatagcagcgacag

Hap35 gatagcagcaacagcagtgatagcagtgaaagcagtaatagtagtgacaacagcaatagcagtgacagcagcaacagcagtgacagcagtgatagcagtgacagcagtaatagtagtgacagcagcaatagc**g**gtgacagcagcaacagcagtgacagcag------------------tgatagcaatagcagcgacag

Merged gatagcagcaacagcagtgatagcagtgaaagcagtaatagtagtgacaacagcaatagcagtgacagcagcaacagcagtgacagcagtgatagcagtgacagcagtaatagtagtgacagcagcaatagcagtgacagcagcaacagcagtgacagcag------------------tgatagcaatagcagcgacag

Transl D S S N S S D S S E S S N S S D N S N S S D S S N S S D S S D S S D S S N S S D S S N S S/**G** D S S N S S D S S D S N S S D S 976

**Notes:** SNPs: c.2878A>G (p.Ser960Gly).

***ID10:*** NM_014208.3:c.2836_2844delGATAGCAGT.

***ID11:*** NM_014208.3:c.2877_2906dup (Same as designation: NM_014208.3:c.2906_2907insCAACAGCAGTGACAGCAG).

2928 ***ID12 ID13*** ***ID14*** 3100

RefSeq cagtgacagcagcaacagcagcgatagcagtgacagcagtgatagcagtgacagcagtgacagcagtgatagcag---------------------------caacagcagtgatagcagtgacagcagtgacagcagtgatagcagtaatagtagtgacagcagcaatagcagtgacagcagcaacagcagtgacagca

McKnight cagtgacagcagcaacagcagcgatagcagtgacagcagtgatagcagtgacagcagtgacagcagtgatagcag---------------------------caacagcagtgatagcagtgacagcagtgacagcagtgatagcagtaatagtagtgacagcagcaa------------------cagcagtgacagca

F1a CAGTGACAGCAGCAACAGCAGCGATAGCAGTGACAGCAGTGATAGCAGTGACAGCAGTGACAGCAGTGATAGCAG---------------------------CAACAGCAGTGATAGCAGTGACAGCAGTGACAGCAGTGATAGCAGTAATAGTAGTGACAGCAGCAA------------------CAGCAGTGACAGCA

F1b CAGTGACAGCAGCAACAGCAGCGATAGCAGTGACAGCAGTGATAGCAGTGACAGCAGTGACAGCAGTGATAGCAG---------------------------CAACAGCAGTGATAGCAGTGACAGCAGTGACAGCAGTGATAGCAGTAATAGTAGTGACAGCAGCAA------------------CAGCAGTGACAGCA

F2a CAGTGACAGCAGCAACAGCAGCGATAGCAGTGACAGCAGTGATAGCAGTGACAGCAGTGACAGCAGTGATAGCAG---------------------------CAACAGCAGTGATAGCAGTGACAGCAGTGACAGCAGTGATAGCAGTAATAGTAGTGACAGCAGCAA------------------CAGCAGTGACAGCA

F2b CAGTGACAGCAGCAACAGCAGCGATAGCAGTGACAGCAGTGATAGCAGTGACAGCAGTGACAGCAGTGATAGCAG---------------------------CAACAGCAGTGATAGCAGTGACAGCAGTGACAGCAGTGATAGCAGTAATAGTAGTGACAGCAGCAATAGCAGTGACAGCAGCAACAGCAGTGACAGCA

F3a CAGTGACAGCAGCAACAGCAGCGATAGCAGTGACAGCAGTGATAGCAGTGACAGCAGTGACAGCAGTGATAGCAG---------------------------CAACAGCAGTGATAGCAGTGACAGCAGTGACAGCAGTGATAGCAGTAATAGTAGTGACAGCAGCAA------------------CAGCAGTGACAGCA

F3b CAGTGACAGCAGCAACAGCAGCGATAGCAGTGACAGCAGTGATAGCAGTGACAGCAGTGACAGCAGTGATAGCAG---------------------------C**G**ACAGCAGTGATAGCAGTGACAGCAGTGACAGCAGTGATAGCAGTAATAGTAGTGACAGCAGCAA------------------CAGCAGTGACAGCA

F4a CAGTGACAGCAGCAACAGCAGCGATAGCAGTGACAGCAGTGATAGCAGTGACAGCAGTGACAGCAGTGATAGCAGTGACAGCAGTGACAGCAGTGATAGCAGCAACAGCAGTGATAGCAGTGACAGCAGTGACAGCAGTGATAGCAGTAATAGTAGTGACAGCAGCAATAGCAGTGACAGCAGCAACAGCAGTGACAGCA

F4b CAGTGACAGCAGCAACAGCAGCGATAGCAGTGACAGCAGTGATAGCAGTGACAGCAGTGACAGCAGTGATAGCAG---------------------------CAACAGCAGTGATAGCAGTGACAGCAGTGACAGCAGTGATAGCAGTAATAGTAGTGACAGCAGCAATAGCAGTGACAGCAGCAACAGCAGTGACAGCA

F5a CAGTGACAGCAGCAACAGCAGCGATAGCAGTGACAGCAGTGATAGCAGTGACAGCAGTGACAGCAGTGATAGCAG---------------------------C**G**ACAGCAGTGATAGCAGTGACAGCAGTGACAGCAGTGATAGCAGTAATAGTAGTGACAGCAGCAA------------------CAGCAGTGACAGCA

F5b CAGTGACAGCAGCAACAGCAGCGATAGCAGTGACAGCAGTGATAGCAGTGACAGCAGTGACAGCAGTGATAGCAG---------------------------CAACAGCAGTGATAGCAGTGACAGCAGTGACAGCAGTGATAGCAGTAATAGTAGTGACAGCAGCAA------------------CAGCAGTGACAGCA

Hap1a CAGTGACAGCAGCAACAGCAGCGATAGCAGTGACAGCAGTGATAGCAGTGACAGCAGTGACAGCAGTGATAGCAG---------------------------CAACAGCAGTGATAGCAGTGACAGCAGTGACAGCAGTGATAGCAGTAATAGTAGTGACAGCAGCAA------------------CAGCAGTGACAGCA

Hap2a CAGTGACAGCAGCAACAGCAGCGATAGCAGTGACAGCAGTGATAGCAGTGACAGCAGTGACAGCAGTGATAGCAG---------------------------C**G**ACAGCAGTGATAGCAGTGACAGCAGTGACAGCAGTGATAGCAGTAATAGTAGTGACAGCAGCAA------------------CAGCAGTGACAGCA

Hap3a CAGTGACAGCAGCAACAGCAGCGATAGCAGTGACAGCAGTGATAGCAGTGACAGCAGTGACAGCAGTGATAGCAG---------------------------CAACAGCAGTGATAGCAGTGACAGCAGTGACAGCAGTGATAGCAGTAATAGTAGTGACAGCAGCAATAGCAGTGACAGCAGCAACAGCAGTGACAGCA

Hap15a CAGTGACAGCAGCAACAGCAGCGATAGCAGTGACAGCAGTGATAGCAGTGACAGCAGTGACAGCAGTGATAGCAG---------------------------CAACAGCAGTGATAGCAGTGACAGCAGTGACAGCAGTGATAGCAGTAATAGTAGTGACAGCAGCAA------------------CAGCAGTGACAGCA

Hap17b cagtgacagcagcaacagcagcgatagcagtgacagcagtgatagcagtgacagcagtgacagcagtgatagcag---------------------------caacagcagtgatagcagtgacagcagtgacagcagtgatagcagtaatagtagtgacagcagcaa------------------cagcagtgacagca

Hap20a CAGTGACAGCAGCAACAGCAGCGATAGCAGTGACAGCAGTGATAGCAGTGACAGCAGTGACAGCAGTGATAGCAG---------------------------C**G**ACAGCAGTGATAGCAGTGACAGCAGTGACAGCAGTGATAGCAGTAATAGTAGTGACAGCAGCAA------------------CAGCAGTGACAGCA

Hap20b cagtgacagcagcaacagcagcgatagcagtgacagcagtgatagcagtgacagcagtgacagcagtgatagcag---------------------------caacagcagtgatagcagtgacagcagtgacagcagtgatagcagtaatagtagtgacagcagcaa------------------cagcagtgacagca

Hap36a CAGTGACAGCAGCAACAGCAGCGATAGCAGTGACAGCAGTGATAGCAGTGACAGCAGTGACAGCAGTGATAGCAG---------------------------CAACAGCAGTGATAGCAGTGACAGCAGTGACAGCAGTGATAGCAGTAATAGTAGTGACAGCAGCAA**C**AGCAGTGACAGCAGCAACAGCAGTGACAGCA

Hap37a CAGTGACAGCAGCAACAGCAGCGATAGCAG------------------TGACAGCAGTGACAGCAGTGATAGCAG---------------------------CAACAGCAGTGATAGCAGTGACAGCAGTGACAGCAGTGATAGCAGTAATAGTAGTGACAGCAGCAA------------------CAGCAGTGACAGCA

Hap38a CAGTGACAGCAGCAACAGCAGCGATAGCAGTGACAGCAGTGATAGCAGTGACAGCAGTGACAGCAGTGATAGCAG---------------------------CAACAGCAGTGATAGCAGTGACAGCAGTGACAGCAGTGATAGCAGTAATAGTAGTGACAGCAGCAATAGCAGTGACAGCAGCAACAGCAGTGACAGCA

SHap1: cagtgacagcagcaacagcagcgatagcagtgacagcagtgatagcagtgacagcagtgacagcagtgatagcag---------------------------caacagcagtgatagcagtgacagcagtgacagcagtgatagcagtaatagtagtgacagcagcaa------------------cagcagtgacagca

SHap2: cagtgacagcagcaacagcagcgatagcagtgacagcagtgatagcagtgacagcagtgacagcagtgatagcag---------------------------caacagcagtgatagcagtgacagcagtgacagcagtgatagcagtaatagtagtgacagcagcaatagcagtgacagcagcaacagcagtgacagca

SHap3: cagtgacagcagcaacagcagcgatagcagtgacagcagtgatagcagtgacagcagtgacagcagtgatagcag---------------------------c**g**acagcagtgatagcagtgacagcagtgacagcagtgatagcagtaatagtagtgacagcagcaa------------------cagcagtgacagca

SHap4: cagtgacagcagcaacagcagcgatagcagtgacagcagtgatagcagtgacagcagtgacagcagtgatagcag---------------------------c**g**acagcagtgatagcagtgacagcagtgacagcagtgatagcagtaatagtagtgacagcagcaa------------------cagcagtgacagca

SHap5: cagtgacagcagcaacagcagcgatagcagtgacagcagtgatagcagtgacagcagtgacagcagtgatagcag---------------------------c**g**acagcagtgatagcagtgacagcagtgacagcagtgatagcagtaatagtagtgacagcagcaa------------------cagcagtgacagca

SHap6: cagtgacagcagcaacagcagcgatagcagtgacagcagtgatagcagtgacagcagtgacagcagtgatagcag---------------------------caacagcagtgatagcagtgacagcagtgacagcagtgatagcagtaatagtagtgacagcagcaa------------------cagcagtgacagca

SHap6(2) cagtgacagcagcaacagcagcgatagcagtgacagcagtgatagcagtgacagcagtgacagcagtgatagcag---------------------------caacagcagtgatagcagtgacagcagtgacagcagtgatagcagtaatagtagtgacagcagcaa------------------cagcagtgacagca

SHap7 cagtgacagcagcaacagcagcgatagcagtgacagcagtgatagcagtgacagcagtgacagcagtgatagcag---------------------------caacagcagtgatagcagtgacagcagtgacagcagtgatagcagtaatagtagtgacagcagcaa------------------cagcagtgacagca

SHap102 cagtgacagcagcaacagcagcgatagcagtgacagcagtgatagcagtgacagcagtgacagcagtgatagcag---------------------------c**g**acagcagtgatagcagtgacagcagtgacagcagtgatagcagtaatagtagtgacagcagcaa------------------cagcagtgacagca

SHap130 cagtgacagcagcaacagcagcgatagcagtgacagcagtgatagcagtgacagcagtgacagcagtgatagcag---------------------------caacagcagtgatagcagtgacagcagtgacagcagtgatagcagtaatagtagtgacagcagcaa------------------cagcagtgacagca

SHap106 cagtgacagcagcaacagcagcgatagcagtgacagcagtgatagcagtgacagcagtgacagcagtgatagcag---------------------------caacagcagtgatagcagtgacagcagtgacagcagtgatagcagtaatagtagtgacagcagcaa------------------cagcagtgacagca

SHap72 cagtgacagcagcaacagcagcgatagcagtgacagcagtgatagcagtgacagcagtgacagcagtgatagcag---------------------------caacagcagtgatagcagtgacagcagtgacagcagtgatagcagtaatagtagtgacagcagcaa------------------cagcagtgacagca

SHap110 cagtgacagcagcaacagcagcgatagcagtgacagcagtgatagcagtgacagcagtgacagcagtgatagcag---------------------------caacagcagtgatagcagtgacagcagtgacagcagtgatagcagtaatagtagtgacagcagcaa------------------cagcagtgacagca

Hap4 cagtgacagcagcaacagcagcgatagcagtgacagcagtgatagcagtgacagcagtgacagcagtgatagcag---------------------------c**g**acagcagtgatagcagtgacagcagtgacagcagtgatagcagtaatagtagtgacagcagcaa------------------cagcagtgacagca

Hap5 cagtgacagcagcaacagcagcgatagcagtgacagcagtgatagcagtgacagcagtgacagcagtgatagcag---------------------------c**g**acagcagtgatagcagtgacagcagtgacagcagtgatagcagtaatagtagtgacagcagcaa------------------cagcagtgacagca

Hap8 cagtgacagcagcaacagcagcgatagcagtgacagcagtgatagcagtgacagcagtgacagcagtgatagcag---------------------------caacagcagtgatagcagtgacagcagtgacagcagtgatagcagtaatagtagtgacagcagcaa------------------cagcagtgacagca

Hap13 cagtgacagcagcaacagcagcgatagcagtgacagcagtgatagcagtgacagcagtgacagcagtgatagcag---------------------------caacagcagtgatagcagtgacagcagtgacagcagtgatagcagtaatagtagtgacagcagcaa------------------cagcagtgacagca

Hap24 cagtgacagcagcaacagcagcgatagcagtgacagcagtgatagcagtgacagcagtgacagcagtgatagcag---------------------------caacagcagtgatagcagtgacagcagtgacagcagtgatagcagtaatagtagtgacagcagcaatagcagtgacagcagcaacagcagtgacagca

Hap25 cagtgacagcagcaacagcagcgatagcagtgacagcag**ca**atagcagtgacagcagtgacagcagtgatagcag---------------------------caacagcagtgatagcagtgacagcagtgacagcagtgatagcagtaatagtagtgacagcagcaatagcagtgacagcagca**g**cagcagtgacagca

Hap26 cagtgacagcagcaacagcagcgatagcag------------------tgacagcagtgacagcagtgatagcag---------------------------caacagcagtgatagcagtgacagcagtgacagcagtgatagcagtaatagtagtgacagcagcaa------------------cagcagtgacagca

Hap29 cagtgacagcagcaacagcagcgatagcag------------------tgacagcagtgacagcagtgatagcag---------------------------caacagcagtgatagcagtgacagcagtgacagcagtgatagcagtaatagtagtgacagcagcaa------------------cagcagtgacagca

Hap32 cagtgacagcagcaacagcagcgatagcagtgacagcagtgatagcagtgacagcagtgacagcagtgatagcag---------------------------caacagcagtgatagcagtgacagcagtgacagcagtgatagcagtaatagtagtgacagcagcaatagcagtgacagcagcaacagcagtgacagca

Hap33 cagtgacagcagcaacagcagcgatagcagtgacagcagtgatagcagtgacagcagtgacagcagtgatagcag---------------------------caacagcagtgatagcagtgacagcagtgacagcagtgatagcagtaatagtagtgacagcagcaa------------------cagcagtgacagca

Hap35 cagtgacagcagcaacagcagcgatagcagtgacagcagtgatagcagtgacagcagtgacagcagtgatagcag---------------------------caacagcagtgatagcagtgacagcagtgacagcagtgatagcagtaatagtagtgacagcagcaa------------------cagcagtgacagca

Merged cagtgacagcagcaacagcagcgatagcagtgacagcagtgatagcagtgacagcagtgacagcagtgatagcag**tgacagcagtgacagcagtgatagcag**caacagcagtgatagcagtgacagcagtgacagcagtgatagcagtaatagtagtgacagcagcaatagcagtgacagcagcaacagcagtgacagca

Transl S D S S N S S D S S D S S D/**N** S S D S S D S S D S S **D S S D S S D S S** N/**D** S S D S S D S S D S S D S S N S S D S S N S S D S S N/**S** S S D S

**Notes:** SNPs: c.2967T>C; c.2968G>A (p.Asp990Asn); c.3004A>G (p.Asn1002Asp); c.3069T>C; c.3085A>G (p.Asn1029Asp); c.3086A>G (p.Asn1029Ser); c.3087C>T.

***ID12:*** NM_014208.3:c.2958_2975delTGACAGCAGTGATAGCAG.

***ID13:*** NM_014208.3:c.2976_3002dup (Same as designation: NM_014208.3:c.3002_3003insTGACAGCAGTGACAGCAGTGATAGCAG).

***ID14:*** NM_014208.3:c.3069_3086deltagcagtgacagcagcaa.

3101 3135 ***ID15 ID16 ID17*** ***ID18 ID19*** 3265 ***ID20***

RefSeq gcgatagcagtgacagcagcgatagcagtgacag**c**agcgatagcagtgacagcagtgacagcagcaatagcagtgacagcagtgacagcagcgaca---------------------------gcagtgatagcagtgacagcagtgacagcagcgacagcagtgatagcagtgaaagcagtgatagcagtg[------]

McKnight gcgatagcagtgacagcagcgatagcagtgacag-------------------agtgacagcagcaatagcagtgacagcagtgacagcagcgaca---------------------------gcagtgatagcagtgacagcagtg**g**cagcagcgacagcagtgatagcagtga**c**agcagtgatagcag**c**g[------]

F1a GCGATAGCAGTGACAGCAGCGATAGCAGTGACAG-------------------AGTGACAGCAGCAATAGCAGTGACAGCAGTGACAGCAGCGACA---------------------------GCAGTGATAGCAGTGACAGCAGTG**G**CAGCAGCGACAGCAGTGATAGCAGTGA**C**AGCAGTGATAGCAG**C**G[------]

F1b GCGATAGCAGTGACAGCAGCGATAGCAGTGACAGC------------------AGTGACAGCAGCAATAGCAGTGACAGCAGTGACAGCAGCGACA---------------------------GCAGTGATAGCAGTGACAGCAGTG**G**CAGCAGCGACAGCAGTGATAGCAGTGA**C**AGCAGTGATAGCAG**C**G[------]

F2a GCGATAGCAGTGACAGCAGCGATAGCAGTGACAGCAGCGATAGCAGTGACAGCAGTGACAGCAGCAATAGCAGTGACAGCAGTGACAGCAGCGACA---------------------------GCAGTGATAGCAGTGACAGCAGTGACAGCAGCGACAGCAGTGATAGCAGTGA**C**AGCAGTGATAGCAG**C**G[------]

F2b GCGATAGCAGTGACAGCAGCGATAGCAGTGACAGCAGCGATAGCAGTGACAGCAGTGACAGCAGCAATAGCAGTGACAGCAGTGACAGCAGCGACA---------------------------GCAGTGATAGCAGTGACAGCAGTGACAGCAGCGACAGCAGTGATAGCAGTGAAAGCAGTGATAGCAGTG[------]

F3a GCGATAGCAGTGACAGCAGCGATAGCAGTGACAGCAGCGATAGCAGTGACAGCAGTGACAGCAGCAATAGCAGTGACAGCAGTGACAGCAGCGACA---------------------------GCAGTGATAGCAGTGACAGCAGTGACAGCAGCGACAGCAGTGATAGCAGTGA**C**AGCAGTGATAGCAG**C**G[------]

F3b GCGATAGCAGTGACAGCAGCGATAGCAGTGACAGCAGCGATAGCAGTGACAGCAGTGACAGCAGCAATAGCAGTGACAGCAGTGACAGCAGCGACA---------------------------GCAGTGATAGCAGTGACAGCAGTGACAGCAGCGACAGCAGTGATAGCAGTGAAAGCAGTGATAGCAG**C**G[------]

F4a GCGATAGCAGTGACAGCAGCGATAGCAGTGACAGC------------------AG**C**GACAGCAGCAATAGCAGTGACAGCAGTGACAGCAGCGACA---------------------------GCAGTGATAGCAGTGACAGCAG---------CGACAGCAGTGATAGCAGTGA**C**AGCAGTG---------[------]

F4b GCGATAGCAGTGACAGCAGCGATAGCAGTGACAGCAGCGATAGCAGTGACAGCAGTGACAGCAGCAATAGCAGTGACAGCAGTGACAGCAGCGACA---------------------------GCAGTGATAGCAGTGACAGCAGTGACAGCAGCGACAGCAGTGATAGCAGTGAAAGCAGTGATAGCAGTG[------]

F5a GCGATAGCAGTGACAGCAGCGATAGCAGTGACAGCAGCGATAGCAGTGACAGCAGTGACAGCAGCAATAGCAGTGACAGCAGTGACAGCAGCGACA---------------------------GCAGTGATAGCAGTGACAGCAGTGACAGCAGCGACAGCAGTGATAGCAGTGAAAGCAGTGATAGCAG**C**G[------]

F5b GCGATAGCAGTGACAGCAGCGATAGCAGTGACAGCAGCGATAGCAGTGACAGCAGTGACAGCAGCAATAGCAGTGACAGCAGTGACAGCAGCGACA---------------------------GCAGTGATAGCAGTGACAGCAGTGACAGCAGCGACAGCAGTGATAGCAGTGA**C**AGCAGTGATAGCAG**C**G[------]

Hap1a GCGATAGCAGTGACAGCAGCGATAGCAGTGACAGC------------------AGTGACAGCAGCAATAGCAGTGACAGCAGTGACAGCAGCGACA---------------------------GCAGTGATAGCAGTGACAGCAGTG**G**CAGCAGCGACAGCAGTGATAGCAGTGA**C**AGCAGTGATAGCAG**C**G[------]

Hap2a GCGATAGCAGTGACAGCAGCGATAGCAGTGACAGCAGCGATAGCAGTGACAGCAGTGACAGCAGCAATAGCAGTGACAGCAGTGACAGCAGCGACA---------------------------GCAGTGATAGCAGTGACAGCAGTGACAGCAGCGACAGCAGTGATAGCAGTGAAAGCAGTGATAGCAG**C**G[------]

Hap3a GCGATAGCAGTGACAGCAGCGATAGCAGTGACAGCAGCGATAGCAGTGACAGCAGTGACAGCAGCAATAGCAGTGACAGCAGTGACAGCAGCGACA---------------------------GCAGTGATAGCAGTGACAGCAGTGACAGCAGCGACAGCAGTGATAGCAGTGAAAGCAGTGATAGCAGTG[------]

Hap15a GCGATAGCAGTGACAGCAGCGATAGCAGTGACAGCAGCGATAGCAGTGACAGCAGTGACAGCAGCAATAGCAGTGACAGCAGTGACAGCAGCGACA---------------------------GCAGTGATAGCAGTGACAGCAGTGACAGCAGCGACAGCAGTGATAGCAGTGA**C**AGCAGTGATAGCAG**C**G[------]

Hap17b gcgatagcagtgacagcagcgatagcagtgacagcagcgatagcagtgacagcagtgacagcagcaatagcagtgacagcagtgacagcagcgaca---------------------------gcagtgatagcagtgacagcagtgacagcagcgacagcagtgatagcagtgacagcagtgatagcagcg[------]

Hap20a GCGATAGCAGTGACAGCAGCGATAGCAGTGACAGCAGCGATAGCAGTGACAGCAGTGACAGCAGCAATAGCAGTGACAGCAGTGACAGCAGCGACA---------------------------GCAGTGATAGCAGTGACAGCAGTGACAGCAGCGACAGCAGTGATAGCAGTGAAAGCAGTGATAGCAG**C**G[------]

Hap20b gcgatagcagtgacagcagcgatagcagtgacagcagcgatagcagtgacagcagtgacagcagcaatagcagtgacagcagtgacagcagcgaca---------------------------gcagtgatagcagtgacagcagtgacagcagcgacagcagtgatagcagtga**c**agcagtgatagcagcg[------]

Hap36a GCGATAGCAGTGACAGCAGCGATAGCAGTGACAGCAGCGATAGCAGTGACAGCAGTGACAGCAGCAATAGCAGTGACAGCAGTGACAGCAGCGACA---------------------------GCAGTGATAGCAGTGACAGCAGTGACAGCAGCGACAGCAGTGATAGCAGTGAAAGCAGTGATAGCAG**C**G[------]

Hap37a GCGATAGCAGTGACAGCAGCGATAGCAGTGACAGC------------------AGTGACAGCAGCAATAGCAGTGACAGCAGTGACAGCAGCGACA---------------------------GCAGTGATAGCAGTGACAGCAGTGACAGCAGCGACAGCAGTGATAGCAGTGA**C**AGCAGTGATAGCAG**C**G[------]

Hap38a GCGATAGCAGTGACAGCAGCGATAGCAGTGACAGC------------------AGTGACAGCAGCAATAGCAGTGACAGCAGTGACAGCAGCGACA---------------------------GCAGTGATAGCAGTGACAGCAGTGACAGCAGCGACAGCAGTGATAGCAGTGAAAGCAGTGATAGCAG**C**G[------]

SHap1: gcgatagcagtgacagcagcgatagcagtgacagcagcgatagcagtgacagcagtgacagcagcaatagcagtgacagcagtgacagcagcgaca---------------------------gcagtgatagcagtgacagcagtgacagcagcgacagcagtgatagcagtga**c**agcagtgatagcag**c**g[------]

SHap2: gcgatagcagtgacagcagcgatagcagtgacagcagcgatagcagtgacagcagtgacagcagcaatagcagtgacagcagtgacagcagcgaca---------------------------gcagtgatagcagtgacagcagtgacagcagcgacagcagtgatagcagtgaaagcagtgatagcagtg[------]

SHap3: gcgatagcagtgacagcagcgatagcagtgacagcagcgatagcagtgacagcagtgacagcagcaatagcagtgacagcagtgacagcagcgaca---------------------------gcagtgatagcagtgacagcagtgacagcagcgacagcagtgatagcagtga**c**agcagtgatagcag**c**g[------]

SHap4: gcgatagcagtgacagcagcgatagcagtgacagcagcgatagcagtgacagcagtgacagcagcaatagcagtgacagcagtgacagcagcgaca---------------------------gcagtgatagcagtgacagcagtgacagcagcgacagcagtgatagcagtgaaagcagtgatagcag**c**g[------]

SHap5: gcgatagcagtgacagcagcgatagcagtgacagc------------------agtgacagcagcaatagcagtgacagcagtgacagcagcgaca---------------------------gcagtgatagcagtgacagcagtg**g**cagcagcgacagcagtgatagcagtga**c**agcagtgatagcag**c**g[------]

SHap6: gcgatagcagtgacagcagcgatagcagtgacagc------------------agtgacagcagcaatagcagtgacagcagtgacagcagcgaca---------------------------gcagtgatagcagtgacagcagtg**g**cagcagcgacagcagtgatagcagtga**c**agcagtgatagcag**c**g[------]

SHap6(2) gcgatagcagtgacagcagcgatagcagtgacagc------------------agtgacagcagcaatagcagtgacagcagtgacagcagcgaca---------------------------gcagtgatagcagtgacagcagtg**g**cagcagcgacagcagtgatagcagtga**c**agcagtgatagcag**c**g[------]

SHap7 gcgatagcagtgacagcagcgatagcagtgacagc------------------agtgacagcagcaatagcagtgacagcagtgacagcagcgaca---------------------------gcagtgatagcagtgacagcagtgacagcagcgacagcagtgatagcagtgaaagcagtgatagcag**c**g[------]

SHap102 gcgatagcagtgacagcagcgatagcagtgacagcagcgatagcagtgacagcagtgacagcagcaatagcagtgacagcagtgacagcagcgaca---------------------------gcagtgatagcagtgacagcagtgacagcagcgacagcagtgatagcagtgaaagcagtgatagcag**c**g[------]

SHap130 gcgatagcagtgacagcagcgatagcagtgacagc------------------ag---------caatagcagtgacagcagtgacagcagcgaca---------------------------gcagtgatagcagtgacagcagtg**g**cagcagcgacagcagtgatagcagtga**c**agcagtgatagcag**c**g[------]

SHap106 gcgatagcagtgacagcagcgatagcagtgacagc------------------agtgacagcagcaatagcagtgacagcagtgacagcagcgacagcagtgacagcagtgacagcagcgacagcagtgatagcagtgacagcagtg**g**cagcagcgacagcagtgatagcagtga**c**agcagtgatagcag**c**g[------]

SHap72 gcgatagcagtgacagcagcgatagcagtgacagcagcgatagcagtgacagcagtgacagcagcaatagcagtgacagcagtgacagcagcgaca---------------------------gcagtga**c**agcagtgacagcagtgacagcagcgacagcagtgatagcagtga**c**agcagtgatagcag**c**g[------]

SHap110 gcgatagcagtgacagcagcgatagcagtgacagcagcgatagcagtgacagcagtgacagcagcaatagcagtgacagcagtgacagcagcgaca---------------------------gcagtgatagcagtgacagcagtgacagcagcgacagcagtgatagcagtgaaagcagtgatagcag**c**g[------]

Hap4 gcgatagcagtgacagcagcgatagcagtgacagcagcgatagcagtgacagcagtgacagcagcaatagcagtgacagcagtgacagcagcgaca---------------------------gcagtgatagcagtgacagcagtgacagcagcgacagcagtgatagcagtgaaagcagtgatagcag**c**g[------]

Hap5 gcgatagcagtgacagcagcgatagcagtgacagcagcgatagcagtgacagcagtgacagcagcaatagcagtgacagcagtgacagcagcgaca---------------------------gcagtgatagcagtgacagcagtgacagcagcgacagcagtgatagcagtgaaagcagtgatagcag**c**g[------]

Hap8 gcgatagcagtgacagcagcgatagcagtgacagcagcgatagcagtgacagcagtgacagcagcaatagcagtgacagcagtgacagcagcgaca---------------------------gcagtgatagcagtgacagcagtgacagcagcgacagcagtgatagcagtga**c**agcagtgatagcag**c**g[------]

Hap13 gcgatagcagtgacagcagcgatagcagtgacagcagcgatagcagtgacagcagtgacagcagcaatagcagtgacagcagtgacagcagcgaca---------------------------gcagtgatagcagtgacagcagtgacagcagcgacagcagtgatagcagtga**c**agcagtgatagcag**c**g[------]

Hap24 gcgatagcagtgacagcagcgatagcagtgacagcagcgatagcagtgacagcagtgacagcagcaatagcagtgacagcagtgacagcagcgaca---------------------------gcagtgatagcagtgacagcagtgacagcagcgacagcagtgatagcagtgaaagcagtgatagcagtg[**189 bp**]

Hap25 gcgatagcagtgacagcagcgatagcagtgacagcagcgatagcagtgacagcagtgacagcagcaatagcagtgacagcagtgacagcagcgaca---------------------------gcagtga**c**agcagtgacagcagtgacagcagcgacagcagtgatagcagtgaaagcagtgatagcagtg[------]

Hap26 gcgatagcagtgacagcagcgatagcagtgacagc------------------agtgacagcagcaatagcagtgacagcagtgacagcagcgaca---------------------------gcagtgatagcagtgacagcagtgacagcagcgacagcagtgatagcagtga**c**agcagtgatagcag**c**g[------]

Hap29 gcgatagcagtgacagcagcgatagcagtgacagc------------------agtgacagcagcaatagcagtgacagcagtgacagcagcgaca---------------------------gcagtgatagcagtgacagcagtgacagcagcgacagcagtgatagcagtga**c**agcagtgatagcag**c**g[------]

Hap32 gcgatagcagtgacagcagcgatagcagtgacagc------------------agtgacagcagcaatagcagtgacagcagtgacagcagcgaca---------------------------gcagtgatagcagtgacagcagtgacagcagcgacagcagtgatagcagtgaaagcagtgatagcag**c**g[------]

Hap33 gcgatagcagtgacagcagcgatagcagtgacagc------------------agtgacagcagcaatagcagtgacagcagtgacagcagcgaca---------------------------gcagtgatagcagtgacagcagtg**g**cagcagcgacagcagtgatagcagtga**c**agcagtgatagcag**c**g[------]

Hap35 gcgatagcagtgacagcagcgatagcagtgacagc------------------agtgacagcagcaatagcagtgacagcagtgacagcagcgaca---------------------------gcagtgatagcagtgacagcagtgacagcagcgacagcagtgatagcagtga**c**agcagtgatagcag**c**g[------]

Merged gcgatagcagtgacagcagcgatagcagtgacagcagcgatagcagtgacagcagtgacagcagcaatagcagtgacagcagtgacagcagcgaca**gcagtgacagcagtgacagcagcgaca**gcagtgatagcagtgacagcagtgacagcagcgacagcagtgatagcagtgaaagcagtgatagcagtg**[189 bp]**

Transl S D S S D S S D S S D S S D S S D S S D S S N S S D S S D S S D  **S S D S S D S S D**  S S D S S D S S D/**G** S S D S S D S S E/**D** S S D S S **[63 aa]**

Hap24 acagcagcaatagcagtgacagcagtgacagcagcgacagcagtgatagcagtgacagcagcgatagcagtgacagcagtgacagcagcaatagcagtgacagcagtgacagcagcgacagcagtgatagcagtgacagcagtgacagcagcgacagcagtgatagcagtgaaagcagtgatagcagtg

Merged **acagcagcaatagcagtgacagcagtgacagcagcgacagcagtgatagcagtgacagcagcgatagcagtgacagcagtgacagcagcaatagcagtgacagcagtgacagcagcgacagcagtgatagcagtgacagcagtgacagcagcgacagcagtgatagcagtgaaagcagtgatagcagtg**

Merged **D S S N S S D S S D S S D S S D S S D S S D S S D S S D S S N S S D S S D S S D S S D S S D S S D S S D S S D S S E S S D S S**

**Notes:** The [189 bp] at the end of the alignment for Hap24 refers to the sequence immediately above these notes.

Dentin Dysplasia II: c.3135delC; p.Ser1045Argfs*269. This mutation was originally described as c.3141delC; p.S1047fsX223; (3); but c.3141 gave the *Name Checker* result: “C not found at position 3261, found T instead”. This variant assignment would have been difficult to make accurately without the alignment, as the deleted nucleotide is followed by ***ID15***, an 18 bp indel (deletion relative to the reference sequence) in the *DSPP* haplotype carrying this disease-causing variant.

SNPs multiple haplotypes: c.3221A>G (p.Asp1074Gly); c.3249A>C (p.Glu1083Asp); c.3264T>C.

***ID15:*** NM_014208.3:c.3136_3153delAGCGATAGCAGTGACAGC.

***ID16:*** NM_014208.3:c.3156_3164delTGACAGCAG.

***ID17:*** NM_014208.3:c.3170_3196dup (Same as designation: NM_014208.3:c.3196_3197insGCAGTGACAGCAGTGACAGCAGCGACA).

***ID18:*** NM_014208.3:c.3219_3227delTGACAGCAG.

***ID19:*** NM_014208.3:c.3257_3445delATAGCAGTGATAGCAGTGACAGCAGCAACAGCAGTGACAGCAGTGACAGCAGTG.

***ID20:*** NM_014208.3:c.3265_3266insacagcagcaatagcagtgacagcagtgacagcagcgacagcagtgatagcagtgacagcagcgatagcagtgacagcagtgacagcag

caatagcagtgacagcagtgacagcagcgacagcagtgatagcagtgacagcagtgacagcagcgacagcagtgatagcagtgaaagcagtgatagcagtg.

3266 ***ID21*** ***ID19 continued*** ***ID22*** 3438 3447 ***ID23***

RefSeq acagcagcaatagcagtgacagcagcgatagcagcgacagcagcgacagcagcgatagcagtgacagcagcgatagcagtgacagcagtgacagcagcaatagcagtgacagcagtgacagcagcgacagcagtgatagcagtgacagcagcaacagcagtgacagcagtga**c**agcagtgaa------------------

McKnight ---------------------------------------------------------------------------------------------------------------------------------------atagcagtgacagcagc**g**acagcagtgacagcagtgacagcagtgaaagcagcgacagcagcgat

F1a ---------------------------------------------------------------------------------------------------------------------------------------ATAGCAGTGACAGCAGC**G**ACAGCAGTGACAGCAGTGACAGCAGTGAAAGCAGCGACAGCAGCGAT

F1b ---------------------------------------------------------------------------------------------------------------------------------------ATAGCAGTGACAGCAGC**G**ACAGCAGTGACAGCAGTGACAGCAGTGAAAGCAGCGACAGCAGCGAT

F2a ---------------------------------------------------------------------------------------------------------------------------------------ATAGCAGTGACAGCAGC**G**ACAGCAGTGACAGCAGTGACAGCAGTGAAAGCAGCGACAGCAGCGAT

F2b ACAGCAGCAATAGCAGTGACAGCAGCGATAGCAGCGACAGCAGCGACAGCAGCGATAGCAGTGACAGCAGCGATAGCAGTGACAGCAGTGACAGCAGCAATAGCAGTGACAGCAGTGACAGCAGCGACAGCAGTGATAGCAGTGACAGCAGCAACAGCAGTGACAGCAGTGACAGCAGTGAA------------------

F3a ---------------------------------------------------------------------------------------------------------------------------------------ATAGCAGTGACAGCAGC**G**ACAGCAGTGACAGCAGTGACAGCAGTGAAAGCAGCGACAGCAGCGAT

F3b ---------------------------------------------------------------------------------------------------------------------------------------ATAGCAGTGACAGCAGC**G**ACAGCAGTGACAGCAGTGACAGCAGTGAA------------------

F4a ------------------------------------------------------------------------------------------------------------------------------------------------------------------------------------AAAGCAGCGACAGCAGCGAT

F4b ACAGCAGCAATAGCAGTGACAGCAGCGATAGCAGCGACAGCAGCGACAGCAGCGATAGCAGTGACAGCAGCGATAGCAGTGACAGCAGTGACAGCAGCAATAGCAGTGACAGCAGTGACAGCAGCGACAGCAGTGATAGCAGTGACAGCAGCAACAGCAGTGACAGCAGTGACAGCAGTGAA------------------

F5a ---------------------------------------------------------------------------------------------------------------------------------------ATAGCAGTGACAGCAGC**G**ACAGCAGTGACAGCAGTGACAGCAGTGAA------------------

F5b ---------------------------------------------------------------------------------------------------------------------------------------ATAGCAGTGACAGCAGC**G**ACAGCAGTGACAGCAGTGACAGCAGTGAAAGCAGCGACAGCAGCGAT

Hap1a ---------------------------------------------------------------------------------------------------------------------------------------ATAGCAGTGACAGCAGC**G**ACAGCAGTGACAGCAGTGACAGCAGTGAAAGCAGCGACAGCAGCGAT

Hap2a ---------------------------------------------------------------------------------------------------------------------------------------ATAGCAGTGACAGCAGC**G**ACAGCAGTGACAGCAGTGACAGCAGTGAA------------------

Hap3a ACAGCAGCAATAGCAGTGACAGCAGCGATAGCAGCGACAGCAGCGACAGCAGCGATAGCAGTGACAGCAGCGATAGCAGTGACAGCAGTGACAGCAGCAATAGCAGTGACAGCAGTGACAGCAGCGACAGCAGTGATAGCAGTGACAGCAGCAACAGCAGTGACAGCAGTGACAGCAGTGAA------------------

Hap15a ---------------------------------------------------------------------------------------------------------------------------------------ATAGCAGTGACAGCAGC**G**ACAGCAGTGACAGCAGTGACAGCAGTGAAAGCAGCGACAGCAGCGAT

Hap17b ---------------------------------------------------------------------------------------------------------------------------------------atagcagtgacagcagcgacagcagtgacagcagtgacagcagtgaaagcagcgacagcagcgat

Hap20a ---------------------------------------------------------------------------------------------------------------------------------------ATAGCAGTGACAGCAGC**G**ACAGCAGTGACAGCAGTGACAGCAGTGAA------------------

Hap20b ---------------------------------------------------------------------------------------------------------------------------------------atagcagtgacagcagcgacagcagtgacagcagtgacagcagtgaaagcagcgacagcagcgat

Hap36a ---------------------------------------------------------------------------------------------------------------------------------------ATAGCAGTGACAGCAGC**G**ACAGCAGTGACAGCAGTGACAGCAGTGAA------------------

Hap37a ---------------------------------------------------------------------------------------------------------------------------------------ATAGCAGTGACAGCAGC**G**ACAGCAGTGACAGCAGTGACAGCAGTGAAAGCAGCGACAGCAGCGAT

Hap38a ---------------------------------------------------------------------------------------------------------------------------------------ATAGCAGTGACAGCAGC**G**ACAGCAGTGACAGCAGTGACAGCAGTGAA------------------

SHap1: ---------------------------------------------------------------------------------------------------------------------------------------atagcagtgacagcagc**g**acagcagtgacagcagtgacagcagtgaaagcagcgacagcagcgat

SHap2: acagcagcaatagcagtgacagcagcgatagcagcgacagcagcgacagcagcgatagcagtgacagcagcgatagcagtgacagcagtgacagcagcaatagcagtgacagcagtgacagcagcgacagcagtgatagcagtgacagcagcaacagcagtgacagcagtgacagcagtgaa------------------

SHap3: ---------------------------------------------------------------------------------------------------------------------------------------atagcagtgacagcagc**g**acagcagtgacagcagtgacagcagtgaaagcagcgacagcagcgat

SHap4: ---------------------------------------------------------------------------------------------------------------------------------------atagcagtgacagcagc**g**acagcagtgacagcagtgacagcagtgaaagcagcgacagcagcgat

SHap5: ---------------------------------------------------------------------------------------------------------------------------------------atagcagtgacagcagc**g**acagcagtgacagcagtgacagcagtgaaagcagcgacagcagcgat

SHap6: ---------------------------------------------------------------------------------------------------------------------------------------atagcagtgacagcagc**g**acagcagtgacagcagtgacagcagtgaaagcagcgacagcagcgat

SHap6(2) ---------------------------------------------------------------------------------------------------------------------------------------atagcagtgacagcagc**g**acagcagtgacagcagtgacagcagtgaaagcagcgacagcagcgat

SHap7 ---------------------------------------------------------------------------------------------------------------------------------------atagcagtgacagcagc**g**acagcagtgacagcagtgacagcagtgaaagcagcgacagcagcgat

SHap102 ---------------------------------------------------------------------------------------------------------------------------------------atagcagtgacagcagc**g**acagcagtgacagcagtgacagcagtgaaagcagcgacagcagcgat

SHap130 ---------------------------------------------------------------------------------------------------------------------------------------atagcagtgacagcagc**g**acagcagtgacagcagtgacagcagtgaaagcagcgacagcagcgat

SHap106 ---------------------------------------------------------------------------------------------------------------------------------------atagcagtgacagcagc**g**acagcagtgacagcagtgacagcagtgaaagcagcgacagcagcgat

SHap72 ---------------------------------------------------------------------------------------------------------------------------------------atagcagtgacagcagc**g**acagcagtgacagcagtgacagcagtgaa------------------

SHap110 ---------------------------------------------------------------------------------------------------------------------------------------atagcagtgacagcagc**g**acagcagtgacagcagtgacagcagtgaaagcagcgacagcagcgat

Hap4 ---------------------------------------------------------------------------------------------------------------------------------------atagcagtgacagcagc**g**acagcagtgacagcagtgacagcagtgaa------------------

Hap5 ---------------------------------------------------------------------------------------------------------------------------------------atagcagtgacagcagc**g**acagcagtgacagcagtgacagcagtgaa------------------

Hap8 ---------------------------------------------------------------------------------------------------------------------------------------atagcagtgacagcagc**g**acagcagtgacagcagtgacagcagtgaaagcagcgacagcagcgat

Hap13 ---------------------------------------------------------------------------------------------------------------------------------------atagcagtgacagcagc**g**acagcagtgac------------------agcagcgacagcagcgat

Hap24 acagcagcaatagcagtgacagcagcgatagcagcgacagcagcgacagcagcgatagcagtgacagcagcgatagcagtgacagcagtgacagcagcaatagcagtgacagcagtgacagcagcgacagcagtgatagcagtgacagcagcaacagcagtgacagcagtgacagcagtgaa------------------

Hap25 acagcagcaatagcagtgacagcagcgatagcagcgacagcagcgacagcagcgatagcagtgacagcagcgatagcagtgacagcagtgacagcagcaatagcagtgacagcagtgacagcagcgacagcagtgatagcagtgacagcagcaacagcagtgacagcagtgacagcagtgaa------------------

Hap26 ---------------------------------------------------------------------------------------------------------------------------------------atagcagtgacagcagc**g**acagcagtgacagcagtgacagcagtgaaagcagcgacagcagcgat

Hap29 ---------------------------------------------------------------------------------------------------------------------------------------atagcagtgacagcagc**g**acagcagtgacagcagtgacagcagtgaaagcagcgacagcagcgat

Hap32 ---------------------------------------------------------------------------------------------------------------------------------------atagcagtgacagcagc**g**acagcagtgacagcagtgacagcagtgaa------------------

Hap33 ---------------------------------------------------------------------------------------------------------------------------------------atagcagtgacagcagc**g**acagcagtgacagcagtgacagcagtgaaagcagcgacagcagcgat

Hap35 ---------------------------------------------------------------------------------------------------------------------------------------atagcagtgacagcagc**g**acagcagtgacagcagtgacagcagtgaaagcagcgacagcagcgat

Merged acagcagcaatagcagtgacagcagcgatagcagcgacagcagcgacagcagcgatagcagtgacagcagcgatagcagtgacagcagtgacagcagcaatagcagtgacagcagtgacagcagcgacagcagtgatagcagtgacagcagcaacagcagtgacagcagtgacagcagtgaa**agcagcgacagcagcgat**

Transl D S S N S S D S S D S S D S S D S S D S S D S S D S S D S S D S S N S S D S S D S S D S S D S S D S S N/**D** S S D S S D S S E **S** **S D S S D**

**Notes:** Dentinogenesis imperfecta type II: c.3438delC; p.Asp1146Glufs*168 (4) Family E.

SNPs: c.3418A>G (p.(Asn1140Asp);

***ID21:*** NM_014208.3:c.3266_3400delACAGCAGCAATAGCAGTGACAGCAGCGATAGCAGCGACAGCAGCGACAGCAGCGATAGCAGTGACAGCAGCGATAG CAGTGACAGCAGTGACAGCAGCAATAGCAGTGACAGCAGTGACAGCAGCGACAGCAGTG.

***ID22:*** NM_014208.3:c.3430_3447delAGCAGTGACAGCAGTGAA.

***ID23:*** NM_014208.3:c.3447_3448insAGCAGCGACAGCAGCGAT.

3448 ***ID24*** 3504_3508dup ***ID25*** 3546-3550 3560 ***ID26*** 3582-3591 ***ID27*** 3624

RefSeq agcagc**g**acagcagtgacagcagcgacagcagtgacagcagcgatagcagcgacag**cagcg**-----acagcagcgatagcagtgacagcagcaatagcagtga**tagca**gcgacagca**g**tgatagcagtgacagcagcga**cagcagcgat**agcagcgacagcagcgatag------------------tagtgatagcagt

McKnight agcagcgacagcagtgacagcagcgacagcagtgacagcagcgatagcagcgacagcagcg-----acagcagcgatagcagtgacagcagcaatagcagtgatagcagcgacagcagtgatagcagtgacagcagcgacagcagcgatagcagcgacagcag**t**gatag------------------tagtgatagcagt

F1a AGCAGCGACAGCAGTGACAGCAGCGACAGCAGTGACAGCAGCGATAGCAGCGACAGCAGCG-----ACAGCAGCGATAGCAGTGACAGCAGCAATAGCAGTGATAGCAGCGACAGCAGTGATAGCAGTGACAGCAGCGACAGCAGCGATAGCAGCGACAGCAG**T**GATAG------------------TAGTGATAGCAGT

F1b AGCAGCGACAGCAGTGACAGCAGCGACAGCAGTGACAGCAGCGATAGCAGCGACAGCAGCG-----ACAGCAGCGATAGCAGTGACAGCAGCAATAGCAGTGATAGCAGCGACAGCAGTGATAGCAGTGACAGCAGCGACAGCAGCGATAGCAGCGACAGCAG**T**GATAG------------------TAGTGATAGCAGT

F2a AGCAGCGACAGCAGTGACAGCAGCGACAGCAG**C**GACAGCAGCGATAGCAG**T**GACAGCAGCGCAGCGACAGCAGCGATAGCAGTGACAGCAGCAATAGCAG**C**GATAGCAGCGACAGCAGTGATAGCAG**C**GACAGCAGCGACAGCAGCGA**C**AGCAGCGACAGCAG**T**GATAG------------------TAGTGATAGCAGT

F2b AGCAGCGACAGCAGTGACAGCAGCGACAGCAGTGACAGCAGCGATAGCAGCGACAGCAGCG-----ACAGCAGCGATAGCAGTGACAGCAGCAATAGCAGTGATAGCAGCGACAGCAGTGATAGCAGTGACAGCAGCGACAGCAGCGATAGCAGCGACAGCAGCGATAG------------------TAGTGATAGCAGT

F3a AGCAGCGACAGCAGTGAC---------------------------AGCAGCGACAGCAGCG-----ACAGCAGCGATAGCAGTGACAGCAGCAATAGCAG**C**GATAGCAGCGACAGCAGTGATAGCAG**C**GACAGCAGCGACAGCAGCGA**C**AGCAGCGACAGCAG**T**GATAG------------------TAGTGATAGCAGT

F3b AGCAGC**A**ACAGCAGTGACAGCAGCGACAGCAGTGACAGCAGCGATAGCAGCGACAGCAGCG-----ACAGCAGCGATAGCAGTGACAGCAGCAATAGCAGTGATAGCAGCGACAGCAGTGATAGCAGTGACAGCAGCGACAGCAGCGATAGCAGCGACAGCAGCGATAG------------------TAGTGATAGCAGT

F4a AGCAGCGACAGCAGTGACAGCAGCGACAGCAGTGACAGCAGCGATAGCAGCGACAGCAGCG--------------ATAGCAGTGACAGCAGCAATAGCAGTGATAGCAGCGACAGCAGTGATAGCAGTGACAGCAGCGACAGCAGCGATAGCAGCGACAGCAG**T**GATAG------------------TAGTGATAGCAGT

F4b AGCAGCGACAGCAGTGACAGCAGCGACAGCAGTGACAGCAGCGATAGCAGCGACAGCAGCG-----ACAGCAGCGATAGCAGTGACAGCAGCAATAGCAGTGATAGCAGCGACAGCAGTGATAGCAGTGACAGCAGCGACAGCAGCGATAGCAGCGACAGCAGCGATAG------------------TAGTGATAGCAGT

F5a AGCAGC**A**ACAGCAGTGACAGCAGCGACAGCAGTGACAGCAGCGATAGCAGCGACAGCAGCG-----ACAGCAGCGATAGCAGTGACAGCAGCAATAGCAGTGATAGCAGCGACAGCAGTGATAGCAGTGACAGCAGCGACAGCAGCGATAGCAGCGACAGCAGCGATAG------------------TAGTGATAGCAGT

F5b AGCAGCGACAGCAGTGAC---------------------------AGCAGCGACAGCAGCG-----ACAGCAGCGATAGCAGTGACAGCAGCAATAGCAG**C**GATAGCAGCGACAGCAGTGATAGCAG**C**GACAGCAGCGACAGCAGCGA**C**AGCAGCGACAGCAG**T**GATAG------------------TAGTGATAGCAGT

Hap1a AGCAGCGACAGCAGTGACAGCAGCGACAGCAGTGACAGCAGCGATAGCAGCGACAGCAGCG-----ACAGCAGCGATAGCAGTGACAGCAGCAATAGCAGTGATAGCAGCGACAGCAGTGATAGCAGTGACAGCAGCGACAGCAGCGATAGCAGCGACAGCAG**T**GATAG------------------TAGTGATAGCAGT

Hap2a AGCAGC**A**ACAGCAGTGACAGCAGCGACAGCAGTGACAGCAGCGATAGCAGCGACAGCAGCG-----ACAGCAGCGATAGCAGTGACAGCAGCAATAGCAGTGATAGCAGCGACAGCAGTGATAGCAGTGACAGCAGCGACAGCAGCGATAGCAGCGACAGCAGCGATAG------------------TAGTGATAGCAGT

Hap3a AGCAGCGACAGCAGTGACAGCAGCGACAGCAGTGACAGCAGCGATAGCAGCGACAGCAGCG-----ACAGCAGCGATAGCAGTGACAGCAGCAATAGCAGTGATAGCAGCGACAGCAGTGATAGCAG**C**GACAGCAG**T**GACAGCAGCGATAGCAGCGACAGCAGCGATAGCAGCGACAGCAGCGATAGTAGTGATAGCAGT

Hap15a AGCAGCGACAGCAGTGAC---------------------------AGCAGCGACAGCAGCG-----ACAGCAGCGATAGCAGTGACAGCAGCAATAGCAG**C**GATAGCAGCGACAGCAGTGATAGCAGCGACAGCAGCGACAGCAGCGA**C**AGCAGCGACAGCAG**T**GATAG------------------TAGTGATAGCAGT

Hap17b agcagcgacagcagtgac---------------------------agcagcgacagcagcg-----acagcagcgatagcagtgacagcagcaatagcagtgatagcagcgacagcagtgatagcagcgacagcagcgacagcagcgacagcagcgacagcagtgatag------------------tagtgatagcagt

Hap20a AGCAGCGACAGCAGTGACAGCAGCGACAGCAGTGACAGCAGCGATAGCAGCGACAGCAGCG-----ACAGCAGCGATAGCAGTGACAGCAGCAATAGCAGTGATAGCAGCGACAGCAGTGA**C**AGCAGTGACAGCAGCGACAGCAGCGATAGCAGCGACAGCAG**T**GA**C**AG------------------TAGTGATAGCAG-

Hap20b agcagcgacagcagtgacagcagcgacagcagtgacagcagcgatagcagcgacagcagcg-----acagcagcgatagcagtgacagcagcaatagcagtgatagcagcgacagcagtgatagcagtgacagcagcgacagcagcgatagcagcgacagcagtgatag------------------tagtgatagcagt

Hap36a AGCAGCGACAGCAGTGACAGCAGCGACAGCAGTGACAGCAGCGATAGCAGCGACAGCAGCG-----ACAGCAGCGATAGCAGTGACAGCAGCGACAGCAGTGATAGCAGCGACAGCAGTGA**C**AGCAGTGACAGCAGCGACAGCAGCGATAGCAGCGACAGCAG**T**GA**C**AG------------------TAGTGATAGCAG-

Hap37a AGCAGCGACAGCAGTGACAGCAGCGACAGCAGTGACAGCAGCGATAGCAGCGACAGCAGCG-----ACAGCAGCGATAGCAGTGACAGCAGCAATAGCAGTGATAGCAGCGACAGCAGTGATAGCAGTGACAGCAGCGACAGCAGCGATAGCAGCGACAGCAG**T**GATAG------------------TAGTGATAGCAGT

Hap38a AGCAGCGACAGCAGTGACAGCAGCGACAGCAGTGACAGCAGCGATAGCAGCGACAGCAGCG-----ACAGCAGCGATAGCAGTGACAGCAGCAATAGCAGTGATAGCAGCGACAGCAGTGATAGCAGTGACAGCAGCGACAGCAGCGATAGCAGCGACAGCAG**T**GATAG------------------TAGTGATAGCAG-

SHap1: agcagcgacagcagtgacagcagcgacagcagtgacagcagcgatagcagcgacagcagcg-----acagcagcgatagcagtgacagcagcaatagcagtgatagcagcgacagcagtgatagcagtgacagcagcgacagcagcgatagcagcgacagcag**t**gatag------------------tagtgatagcagt

SHap2: agcagcgacagcagtgacagcagcgacagcagtgacagcagcgatagcagcgacagcagcg-----acagcagcgatagcagtgacagcagcaatagcagtgatagcagcgacagcagtgatagcagtgacagcagcgacagcagcgatagcagcgacagcagcgatag------------------tagtgatagcagt

SHap3: agcagcgacagcagtgacagcagcgacagcagtgacagcagcgatagcagcgacagcagcg-----acagcagcgatagcagtgacagcagcaatagcagtgatagcagcgacagcagtgatagcag**c**gacagcagcgacagcagcga**c**agcagcgacagcag**t**gatag------------------tagtgatagcagt

SHap4: agcagc**a**acagcagtgacagcagcgacagcagtgacagcagcgatagcagcgacagcagcg-----acagcagcgatagcagtgacagcagcaatagcagtgatagcagcgacagcagtgatagcagtgacagcagcgacagcagcgatagcagcgacagcagcgatag------------------tagtgatagcagt

SHap5: agcagcgacagcagtgacagcagcgacagcagtgacagcagcgatagcagcgacagcagcg-----acagcagcgatagcagtgacagcagcaatagcagtgatagcagcgacagcagtgatagcagtgacagcagcgacagcagcgatagcagcgacagcag**t**gatag------------------tagtgatagcag-

SHap6: agcagcgacagcagtgacagcagcgacagcagtgacagcagcgatagcagcgacagcagcg-----acagcagcgatagcagtgacagcagcaatagcagtgatagcagcgacagcagtgatagcagtgacagcagcgacagcagcgatagcagcgacagcag**t**gatag------------------tagtgatagcagt

SHap6(2) agcagcgacagcagtgacagcagcgacagcagtgacagcagcgatagcagcgacagcagcg-----acagcagcgatagcagtgacagcagcaatagcagtgatagcagcgacagcagtgatagcagtgacagcagcgacagcagcgatagcagcgacagcag**t**gatag------------------tagtgatagcagt

SHap7 agcagc**a**acagcagtgacagcagcgacagcagtgacagcagcgatagcagcgacagcagcg-----acagcagcgatagcagtgacagcagcaatagcagtgatagcagcgacagcagtgatagcagtgacagcagcgacagcagcgatagcagcgacagcagcgatag------------------tagtgatagcagt

SHap102 agcagcgacagcagtgacagcagcgacagcagtgacagcagcgatagcagcgacagcagcg-----acagcagcgatagcagtgacagcagcaatagcagtgatagcagcgacagcagtgatagcagtgacagcagcgacagcagcgatagcagcgacagcag**t**gatag------------------tagtgatagcag-

SHap130 agcagcgacagcagtgacagcagcgacagcagtgacagcagcgatagcagcgacagcagcg-----acagcagcgatagcagtgacagcagcaatagcagtgatagcagcgacagcagtgatagcagtgacagcagcgacagcagcgatagcagcgacagcag**t**gatag------------------tagtgatagcagt

SHap106 agcagcgacagcagtgacagcagcgacagcagtgacagcagcgatagcagcgacagcagcg-----acagcagcgatagcagtgacagcagcaatagcagtgatagcagcgacagcagtgatagcagtgacagcagcgacagcagcgatagcagcgacagcag**t**gatag------------------tagtgatagcagt

SHap72 agcagcgacagcagtgac---------------------------agcagcgacagcagcg-----acagcagcgatagcagtgacagcagcaatagcagtgatagcagcgacagcagtgatagcagtg---------acagcagcga**c**agcagcgacagcag**t**gatag------------------tagtgatagcagt

SHap110 agcagc**a**acagcagtgacagcagcgacagcagtgacagcagcgatagcagcgacagcagcg-----acagcagcgatagcagtgacagcagcaatagcagtgatagcagcgacagcagtgatagcagtgacagcagcgacagcagcgatagcagcgacagcagcgatag------------------tagtgatagcag-

Hap4 agcagcgacagcagtgacagcagcgacagcagtgacagcagcgatagcagcgacagcagcg-----acagcagcgatagcagtgacagcagcaatagcagtgatagcagcgacagcagtga**c**agcagtg---------acagcagcgatagcagcgacagcag**t**ga**c**ag------------------tagtgatagcag-

Hap5 agcagcgacagcagtgacagcagcgacagcagtgacagcagcgatagcagcgacagcagcg-----acagcagcgatagcagtgacagcagcaatagcagtgatagcagcgacagcagtga**c**agcagtg---------acagcagcgatagcagcgacagcag**t**ga**c**ag------------------tagtgatagcag-

Hap8 agcagcgacagcagtgacagcagcgacagcagtgacagcagcgatagcagcgacagcagcg-----acagcagcgatagcagtgacagcagcaatagcagtgatagcagcgacagcagtgatagcagtgacagcagcgacagcagcgatagcagcgacagcag**t**gatag------------------tagtgatagcag-

Hap13 agcagcgacagcagtgac---------------------------agcagcgacagcagcg-----acagcagcgatagcagtgacagcagcaatagcagtgatagcagcgacagcagtgatagcag**c**gacagcagcgacagcagcga**c**agcagcgacagcag**t**gatag------------------tagtgatagcagt

Hap24 agcagcgacagcagtgacagcagcgacagcagtgacagcagcgatagcagcgacagcagcg-----acagcagcgatagcagtgacagcagcaatagcagtgatagcagcgacagcagtgatagcagtgacagcagcgacagcagcgatagcagcgacagcagcgatag------------------tagtgatagcagt

Hap25 agcagcgacagcagtgacagcagcgacagcagtgacagcagcgatagcagcgacagcagcg-----acagcagcgatagcagtgacagcagcaatagcagtgatagcagcgacagcagtgatagcagtgacagcagcgacagcagcgatagcagcgacagcagcgatag------------------tagtgatagcagt

Hap26 agcagcgacagcagtgacagcagcgacagcagtgacagcagcgatagcagcgacagcagcg-----acagcagcgatagcagtgacagcagcaatagcagtgatagcagcgacagcagtgatagcagtgacagcagcgacagcagcgatagcagcgacagcag**t**gatag------------------tagtgatagcagt

Hap29 agcagcgacagcagtgacagcagcgacagcagtgacagcagcgatagcagcgacagcagcg-----acagcagcgatagcagtgacagcagcaatagcagtgatagcagcgacagcagtgatagcagtgacagcagcgacagcagcgatagcagcgacagcag**t**gatag------------------tagtgatagcagt

Hap32 agcagcgacagcagtgacagcagcgacagcagtgacagcagcgatagcagcgacagcagcg-----acagcagcgatagcagtgacagcagcaatagcagtgatagcagcgacagcagtgatagcagtgacagcagcgacagcagcgatagcagcgacagcag**t**gatag------------------tagtgatagcag-

Hap33 agcagcgacagcagtgacagcagcgacagcagtgacagcagcgatagcagcgacagcagcg-----acagcagcgatagcagtgacagcagcaatagcagtgatagcagcgacagcagtgatagcagtgacagcagcgacagcagcgatagcagcgacagcag**t**gatag------------------tagtgatagcagt

Hap35 agcagcgacagcagtgacagcagcgacagcagtgacagcagcgatagcagcgacagcagcg-----acagcagcgatagcagtgacagcagcaatagcagtgatagcagcgacagcagtgatagcagtgacagcagcgacagcagcgatagcagcgacagcag**t**gatag------------------tagtgatagcagt

Merged agcagcgacagcagtgacagcagcgacagcagtgacagcagcgatagcagcgacagcagcg-----acagcagcgatagcagtgacagcagcaatagcagtgatagcagcgacagcagtgatagcagtgacagcagcgacagcagcgatagcagcgacagcagcgatag**cagcgacagcagcgatag**tagtgatagcagt

Transl S S D/**N** S S D S S D S S D S S D S S D S S D S S D S S D S S N S S D S S D S S D S S D S S D S S D S S D S S D S **S D S S D S** S D S S 1208

**Notes:** Snp8: c.3454G>A; p.D1152N

Dentinogenesis imperfecta type II: c.3504_3508dup; p.Asp1170Alafs*146; New disease-causing mutation discovered in this study.

Dentinogenesis imperfecta type II: c.3546_3550delTAGCAinsG; p.Asp1182Glufs*131 (4) Family M.

Dentinogenesis imperfecta type II: c.3560delG; p.Ser1187Metfs*127 (6).

Dentin dysplasia II: c.3582_3591delCAGCAGCGAT; p.Asp1194Glufs*117 (5) Kindred 11.

***ID24:*** NM_014208.3:c.3466_3492delAGCAGCGACAGCAGTGACAGCAGCGAT

***ID25:*** NM_014208.3:c.3509_3517delACAGCAGCG.

***ID26:*** NM_014208.3:c.3591_3599delTAGCAGCGA.

***ID27:*** NM_014208.3:c.3594_3611dupCAGCGACAGCAGCGATAG.

3625 ***ID28 ID29 ID30*** 3626-3701del76 bp ***ID31 ID32*** 3806

RefSeq g**acagcagtgacagcagcgacagcagtgacagcagcgacagcagtgacagcagcgacagcagtgacagcaatgaaag**cagcgacagcagtgacagcagcgatagcagtgacagcagc------------------aacagcagtgacagcagcgacagcagtgatagcagtgacagcacatctgacagcaatgatgagag

McKnight gacagcagtgacagcagcgacagcagtgacagcagcgacagcagtgacagcagcgacagcagtgacagcaatgaaagcagcgacagcagtgacagcagcgatagcagtgacagcagc------------------aacagcagtgacagcagcgacagcagtgatagcagtgacagcacatctgacagcaatgatgagag

F1a GACAGCAGTGACAGCAGCGACAGCAGTGACAGCAGCGACAGCAGTGACAGCAGCGACAGCAGTGACAGCAATGAAAGCAGCGACAGCAGTGACAGCAGCGATAGCAGTGACAGCAGC------------------AACAGCAGTGACAGCAGCGACAGCAGTGATAGCAGTGACAGCACATCTGACAGCAATGATGAGAG

F1b GACAGCAGTGACAGCAGCGACAGCAGTGACAGCAGCGACAGCAGTGACAGCAGCGACAGCAGTGACAGCAATGAAAGCAGCGACAGCAGTGACAGCAGCGATAGCAGTGACAGCAGC------------------AACAGCAGTGACAGCAGCGACAGCAGTGATAGCAGTGACAGCACATCTGACAGCAATGATGAGAG

F2a GACAGCAGTGACAGCAGCGACAGCAGTGACAGCAGCGACAGCAGTGACAGCAGCGACAGCAGTGACAGCAATGAAAGCAGCGACAGCAGTGACAGCAGCGATAGCAGTGACAGCAGC------------------AACAGCAGTGACAGCAGCGACAGCAGTGATAGCAGTGACAGCACATCTGACAGCAATGATGAGAG

F2b GACAGCAGTGACAGCAGCGACAGCAGTGACAGCAGCGACAGCAGTGACAGCAGCGACAGCAGTGACAGCAATGAAAGCAGCGACAGCAGTGACAGCAGCGATAGCAGTGACAGCAGC------------------AACAGCAGTGACAGCAGCGACAGCAGTGATAGCAGTGACAGCACATCTGACAGCAATGATGAGAG

F3a GACAGCAGTGACAGCAGCGACAGCAGTGACAGCAGCGACAGCAGTGACAGCAGCGACAGCAGTGACAGCAATGAAAGCAGCGACAGCAGTGACAGCAGCGATAGCAGTGACAGCAGC------------------AACAGCAGTGACAGCAGCGACAGCAGTGATAGCAGTGACAGCACATCTGACAGCAATGATGAGAG

F3b GACAGCAGTGACAGCAGCGACAGCAGTGACAGCAGCGACAGCAGTGACAGCAGCGACAGCAGTGACAGCAATGAAAGCAGCGACAGCAGTGACAGCAGCGATAGCAGTGACAGCAGC------------------AACAGCAGTGACAGCAGCGACAGCAGTGATAGCAGTGACAGCACATCTGACAGCAATGATGAGAG

F4a GACAGCAGTGACAGCAGCGACAGCAGTGACAGCAGCGACAGCAGTGACAGCAGCGACAGCAGTGACAGCAATGAAAGCAGCGACAGCAGTGACAGCAGCGATAGCAGTGACAGCAGC------------------AACAGCAGTGACAGCAGCGACAGCAGTGATAGCAGTGACAGCACATCTGACAGCAATGATGAGAG

F4b GACAGCAGTGACAGCAGCGACAGCAGTGACAGCAGCGACAGCAGTGACAGCAGCGACAGCAGTGACAGCAATGAAAGCAGCGACAGCAGTGACAGCAGCGATAGCAGTGACAGCAGC------------------AACAGCAGTGACAGCAGCGACAGCAGTGATAGCAGTGACAGCACATCTGACAGCAATGATGAGAG

F5a GACAGCAGTGACAGCAGCGACAGCAGTGACAGCAGCGACAGCAGTGACAGCAGCGACAGCAGTGACAGCAATGAAAGCAGCGACAGCAGTGACAGCAGCGATAGCAGTGACAGCAGC------------------AACAGCAGTGACAGCAGCGACAGCAGTGATAGCAGTGACAGCACATCTGACAGCAATGATGAGAG

F5b GACAGCAGTGACAGCAGCGACAGCAGTGACAGCAGCGACAGCAGTGACAGCAGCGACAGCAGTGACAGCAATGAAAGCAGCGACAGCAGTGACAGCAGCGATAGCAGTGACAGCAGC------------------AACAGCAGTGACAGCAGCGACAGCAGTGATAGCAGTGACAGCACATCTGACAGCAATGATGAGAG

Hap1a GACAGCAGTGACAGCAGCGACAGCAGTGACAGCAGCGACAGCAGTGACAGCAGCGACAGCAGTGACAGCAATGAAAGCAGCGACAGCAGTGACAGCAGCGATAGCAGTGACAGCAGC------------------AACAGCAGTGACAGCAGCGACAGCAGTGATAGCAGTGACAGCACATCTGACAGCAATGATGAGAG

Hap2a GACAGCAGTGACAGCAGCGACAGCAGTGACAGCAGCGACAGCAGTGACAGCAGCGACAGCAGTGACAGCAATGAAAGCAGCGACAGCAGTGACAGCAGCGATAGCAGTGACAGCAGC------------------AACAGCAGTGACAGCAGCGACAGCAGTGATAGCAGTGACAGCACATCTGACAGCAATGATGAGAG

Hap3a GACAGCAGTGACAGCAGCGACAGCAGTGACAGCAGCGACAGCAGTGACAGCAGCGACAGCAGTGACAGCAATGAAAGCAGCGACAGCAGTGACAGCAGCGATAGCAGTGACAGCAGC------------------AACAGCAGTGACAGCAGCGACAGCAGTGATAGCAGTGACAGCACATCTGACAGCAATGATGAGAG

Hap15a GACAGCAGTGACAGCAGCGACAGCAGTGACAGCAGCGACAGCAGTGACAGCAGCGACAGCAGTGACAGCAATGAAAGCAGCGACAGCAGTGACAGCAGCGATAGCAGTGACAGCAGC------------------AACAGCAGTGACAGCAGCGACAGCAGTGATAGCAGTGACAGCACATCTGACAGCAATGATGAGAG

Hap17b gacagcagtgacagcagcgacagcagtgacagcagcgacagcagtgacagcagcgacagcagtgacagcaatgaaagcagcgacagcagtgacagcagcgatagcagtgacagcagc------------------aacagcagtgacagcagcgacagcagtgatagcagtgacagcacatctgacagcaatgatgagag

Hap20a -----------------CGACAGCAGTGACAGCAGCGACAGCAGTGACAGCAGCGACAGCAGTGACAGCAATGAAAGCAGCGACAGCAGTGACAGCAGCGATAGCAGTGACAGCAGCGATAGCAGTGACAGCAGCAACAGCAGTGACAGCAGCGACAGCAGTGATAGCAGTGACAGCACATCTGACAGCAATGATGAGAG

Hap20b gacagcagtgacagcagcgacagcagtgacagcagcgacagcagtgacagcagcgacagcagtgacagcaatgaaagcagcgacagcagtgacagcagcgatagcagtgacagcagc------------------aacagcagtgacagcagcgacagcagtgatagcagtgacagcacatctgacagcaatgatgagag

Hap36a -----------------CGACAGCAGTGACAGCAGCGACAGCAGTGACAGCAGCGACAGCAGTGACAGCAATGAAAGCAGCGACAGCAGTGACAGCAGCGATAGCAGTGACAGCAGCGATAGCAGTGACAGCAGCAACAGCAGTGACAGCAGCGACAGCAGTGATAGCAGTGACAGCACATCTGACAGCAATGATGAGAG

Hap37a GACAGCAGTGACAGCAGCGACAGCAGTGACAGCAGCGACAGCAGTGACAGCAGCGACAGCAGTGACAGCAATGAAAGCAGCGACAGCAGTGACAGCAGCGATAGCAGTGACAGCAGC------------------AACAGCAGTGACAGCAGCGACAGCAGTGATAGCAGTGACAGCACATCTGACAGCAATGATGAGAG

Hap38a -----------------------------------CGACAGCAGTGACAGCAGCGACAGCAGTGACAGCAATGAAAGCAGCGACAGCAGTGACAGCAGCGATAGCAGTGACAGCAGCGATAGCAGTGACAGCAGCAACAGCAGTGACAGCAGCGACAGCAGTGATAGCAGTGACAGCACATCTGACAGCAATGATGAGAG

SHap1: gacagcagtgacagcagcgacagcagtgacagcagcgacagcagtgacagcagcgacagcagtgacagcaatgaaagcagcgacagcagtgacagcagcgatagcagtgacagcagc------------------aacagcagtgacagcagcgacagcagtgatagcagtgacagcacatctgacagcaatgatgagag

SHap2: gacagcagtgacagcagcgacagcagtgacagcagcgacagcagtgacagcagcgacagcagtgacagcaatgaaagcagcgacagcagtgacagcagcgatagcagtgacagcagc------------------aacagcagtgacagcagcgacagcagtgatagcagtgacagcacatctgacagcaatgatgagag

SHap3: gacagcagtgacagcagcgacagcagtgacagcagcgacagcagtgacagcagcgacagcagtgacagcaatgaaagcagcgacagcagtgacagcagcgatagcagtgacagcagc------------------aacagcagtgacagcagcgacagcagtgatagcagtgacagcacatctgacagcaatgatgagag

SHap4: gacagcagtgacagcagcgacagcagtgacagcagcgacagcagtgacagcagcgacagcagtgacagcaatgaaagcagcgacagcagtgacagcagcgatagcagtgacagcagc------------------aacagcagtgacagcagcgacagcagtgatagcagtgacagcacatctgacagcaatgatgagag

SHap5: -----------------**t**gacagcagtgacagcagcgacagcagtgacagcagcgacagcagtgacagcaatgaaagcagcgacagcagtgacagcagcgatagcagtgacagcagc------------------aacagcagtgacagcagcgacagcagtgatagcagtgacagcacatctgacagcaatgatgagag

SHap6: gacagcagtgacagcagcgacagcagtgacagcagcgacagcagtgacagcagcgacagcagtgacagcaatgaaagcagcgacagcagtgacagcagcgatagcagtgacagcagc------------------aacagcagtgacagcagcgacagcagtgatagcagtgacagcacatctgacagcaatgatgagag

SHap6(2) gacagcagtgacagcagcgacagcagtgacagcagcgacagcagtgacagcagcgacagcagtgacagcaatgaaagcagcgacagcagtgacagcagcgatagcagtgacagcagc------------------aacagcagtgacagcagcgacagcagtgatagcagtgacagcacatctgacagcaatgatgagag

SHap7 gacagcagtgacagcagcgacagcagtgacagcagcgacagcagtgacagcagcgacagcagtgacagcaatgaaagcagcgacagcagtgacagcagcgatagcagtgacagcagc------------------aacagcagtgacagcagcgacagcagtgatagcagtgacagcacatctgacagcaatgatgagag

SHap102 -----------------cgacagcagtgacagcagcgacagcagtgacagcagcgacagcagtgacagcaatgaaagcagcgacagcagtgacagcagcgatagcagtgacagcagcgatagcagtgacagcagcaacagcagtgacagcagcgacagcagtgatagcagtgacagcacatctgacagcaatgatgagag

SHap130 gacagcagtgacagcagcgacagcagtgacagcagcgacagcagtgacagcagcgacagcagtgacagcaatgaaagcagcgacagcagtgacagcagcgatagcagtgacagcagc------------------aacagcagtgacagcagcgacagcagtgatagcagtgacagcacatctgacagcaatgatgagag

SHap106 gacagcagtgacagcagcgacagcagtgacagcagcgacagcagtgacagcagcgacagcagtgacagcaatgaaagcagcgacagcagtgacagcagcgatagcagtgacagcagc------------------aacagcagtgacagcagcgacagcagtgatagcagtgacagcacatctgacagcaatgatgagag

SHap72 gacagcagtgacagcagcgacagcagtgacagcagcgacagcagtgacagcagcgacagcagtgacagcaatgaaagcagcgacagcagtgacagcagcgatagcagtgacagcagc------------------aacagcagtgacagcagcgacagcagtgatagcagtgacagcacatctgacagcaatgatgagag

SHap110 -----------------**t**gacagcagtgacagcagcgacagcagtgacagcagcgacagcagtgacagcaatgaaagcagcgacagcagtgacagcagcgatagcagtgacagcagc------------------aacagcagtgacagcagcgacagcagtgatagcagtgacagcacatctgacagcaatgatgagag

Hap4 -----------------cgacagcagtgacagcagcgacagcagtgacagcagcgacagcagtgacagcaatgaaagcagcgacagcagtgacagcagcgatagcagtgacagcagcgatagcagtgacagcagcaacagcagtgacagcagcgacagcagtgatagcagtgacagcacatctgacagcaatgatgagag

Hap5 -----------------cgacagcagtgacagcagcgacagcagtgacagcagcgacagcagtgacagcaatgaaagcagcgacagcagtgacagcagcgatagcagtgacagcagcgatagcagtgacagcagcaacagcagtgacagcagcgacagcagtgatagcagtgacagcacatctgacagcaatgatgagag

Hap8 -----------------**t**gacagcagtgacagcagcgacagcagtgacagcagcgacagcagtgacagcaatgaaagcagcgacagcagtgacagcagcgatagcagtgacagcagc------------------aacagcagtgacagcagcgacagcagtgatagcagtgacagcacatctgacagcaatgatgagag

Hap13 gacagcag---------**t**gacagcagtgacagcagcgacagcagtgacagcagcgacagcagtg**g**cagcaatgaaagcagcgacagcagtgacagcagcgatagcagtgacagcagc------------------aacagcagtgacagcagcgacagcagtgatagcagtgacagcacatctgacagcaatgatgagag

Hap24 gacagcagtgacagcagcgacagcagtgacagcagcgacagcagtgacagcagcgacagcagtgacagcaatgaaagcagcgacagcagtgacagcagcgatagcagtgacagcagc------------------aacagcagtgacagcagcgacagcagtgatagcagtgacagcacatctgacagcaatgatgagag

Hap25 gacagcagtgacagcagcgacagcagtgacagcagc**a**acagcagtgacagcagcgacagcagtgacagcaatgaaagcagcgacagcagtgacagcagcgatagcagtgacagcagc------------------aacagcagtgacagcagcgacagcagtgatagcagtgacagcacatctgacagcaatgatgagag

Hap26 gacagcag---------**t**gacagcagtgacagcagcgacagcagtgacagcagcgacagcagtgacagcaatgaaagcagcgacagcagtgacagcagcgatagcagtgacagcagc------------------aacagcagtgacagcagcgacagcagtgatagcagtgacagcacatctgacagcaatgatgagag

Hap29 gacagcagtgacagcagcgacagcagtgacagcagcgacagcagtgacagcagcgacagcagtgacagcaatgaaagcagcgacagcagtgacagcagcgatagcagtgacagcagc------------------aacagcagtgacagcagcgacagcagtgatagcagtgacagcacatctgacagcaatgatgagag

Hap32 -----------------cgacagcagtgacagcagcgacagcagtgacagcagcgacagcagtgacagcaatgaaagcagcgacagcagtgacagcagcgatagcagtgacagcagcgatagcagtgacagcagcaacagcagtgacagcagcgacagcagtgatagcagtgacagcacatctgacagcaatgatgagag

Hap33 gacagcagtgacagcagcgacagcagtga---------------------------------------------------------------------------------------------------------------------cagcagcgacagcagtgatagcagtgacagcacatctgacagcaatgatgagag

Hap35 gacagcagtgacagcagcgacagcagtgacagcagcgacagcagtgacagcagcgacagcagtgacagcaatgaaagcagcgacagcagtgacagcagcgatagcagtgacagcagc------------------aacagcagtgacagcagcgacagcagtgatagcagtgacagcacatctgacagcaatgatgagag

Merged gacagcagtgacagcagcgacagcagtgacagcagcgacagcagtgacagcagcgacagcagtgacagcaatgaaagcagcgacagcagtgacagcagcgatagcagtgacagcagc**gatagcagtgacagcagc**aacagcagtgacagcagcgacagcagtgatagcagtgacagcacatctgacagcaatgatgagag

Transl D S S D S S D S S D S S D S S D S S D S S D S N E S S D S S D S S D S S D S S  **D S S D S** **S** N S S D S S D S S D S S D S T S D S N D E S

**Notes:** Dentin dysplasia II: c.3626_3701del76; p.Asp1209Alafs*80 (5) Kindred 12. This was orignally described as c.3625_3700del76; p.Asp1209Alafs*80, but was corrected by *Multilyzer*.

ACTGCTGACGCTGCACTGCTATCACT from the published chromatogram (Fig. 2F) gives a reverse complement of (AGTGATAGCAGTGCAGCGTCAGCAGT AGTGATAGCAGTG-del 76-CAGCGTCAGCAGT, which indicates that in addition to the deletion there is a nearby sequence variation c.3707T>A (red highlight; p.Asp1236Val).

***ID28:*** NM_014208.3:c.3624_3641delTGACAGCAGTGACAGCAG.

***ID29:*** NM_014208.3:c.3633_3641delTGACAGCAG.

***ID30:*** NM_014208.3:c.3624_3659delTGACAGCAGTGACAGCAGCGACAGCAGTGACAGCAG

***ID31:*** NM_014208.3:c.3654_3752delCAGCAGCGACAGCAGTGACAGCAGCGACAGCAGTGACAGCAATGAAAGCAG CGACAGCAGTGACAGCAGCGATAGCAGTGACAGCAGCAACAGCAGTGA.

***ID32:*** NM_014208.3:c.3724_3741dup (same as: NM_014208.3:c.3741_3742insGATAGCAGTGACAGCAGC).

3807 3906

RefSeq tgacagccagagcaagtctggtaacggtaacaacaatggaagtgacagtgacagtgacagtgaaggcagtgacagtaaccactcaaccagtgatgattag

McKnight tgacagccagagcaagtctggtaacggtaacaacaatggaagtgacagtgacagtgacagtgaaggcagtgacagtaaccactcaaccagtgatgattag

F1a TGACAGCCAGAGCAAGTCTGGTAACGGTAACAACAATGGAAGTGACAGTGACAGTGACAGTGAAGGCAGTGACAGTAACCACTCAACCAGTGATGATTAG

F1b TGACAGCCAGAGCAAGTCTGGTAACGGTAACAACAATGGAAGTGACAGTGACAGTGACAGTGAAGGCAGTGACAGTAACCACTCAACCAGTGATGATTAG

F2a TGACAGCCAGAGCAAGTCTGGTAACGGTAACAACAATGGAAGTGACAGTGACAGTGACAGTGAAGGCAGTGACAGTAACCACTCAACCAGTGATGATTAG

F2b TGACAGCCAGAGCAAGTCTGGTAACGGTAACAACAATGGAAGTGACAGTGACAGTGACAGTGAAGGCAGTGACAGTAACCACTCAACCAGTGATGATTAG

F3a TGACAGCCAGAGCAAGTCTGGTAACGGTAACAACAATGGAAGTGACAGTGACAGTGACAGTGAAGGCAGTGACAGTAACCACTCAACCAGTGATGATTAG

F3b TGACAGCCAGAGCAAGTCTGGTAACGGTAACAACAATGGAAGTGACAGTGACAGTGACAGTGAAGGCAGTGACAGTAACCACTCAACCAGTGATGATTAG

F4a TGACAGCCAGAGCAAGTCTGGTAACGGTAACAACAATGGAAGTGACAGTGACAGTGACAGTGAAGGCAGTGACAGTAACCACTCAACCAGTGATGATTAG

F4b TGACAGCCAGAGCAAGTCTGGTAACGGTAACAACAATGGAAGTGACAGTGACAGTGACAGTGAAGGCAGTGACAGTAACCACTCAACCAGTGATGATTAG

F5a TGACAGCCAGAGCAAGTCTGGTAACGGTAACAACAATGGAAGTGACAGTGACAGTGACAGTGAAGGCAGTGACAGTAACCACTCAACCAGTGATGATTAG

F5b TGACAGCCAGAGCAAGTCTGGTAACGGTAACAACAATGGAAGTGACAGTGACAGTGACAGTGAAGGCAGTGACAGTAACCACTCAACCAGTGATGATTAG

Hap1a TGACAGCCAGAGCAAGTCTGGTAACGGTAACAACAATGGAAGTGACAGTGACAGTGACAGTGAAGGCAGTGACAGTAACCACTCAACCAGTGATGATTAG

Hap2a TGACAGCCAGAGCAAGTCTGGTAACGGTAACAACAATGGAAGTGACAGTGACAGTGACAGTGAAGGCAGTGACAGTAACCACTCAACCAGTGATGATTAG

Hap3a TGACAGCCAGAGCAAGTCTGGTAACGGTAACAACAATGGAAGTGACAGTGACAGTGACAGTGAAGGCAGTGACAGTAACCACTCAACCAGTGATGATTAG

Hap15a TGACAGCCAGAGCAAGTCTGGTAACGGTAACAACAATGGAAGTGACAGTGACAGTGACAGTGAAGGCAGTGACAGTAACCACTCAACCAGTGATGATTAG

Hap17b tgacagccagagcaagtctggtaacggtaacaacaatggaagtgacagtgacagtgacagtgaaggcagtgacagtaaccactcaaccagtgatgattag

Hap20a TGACAGCCAGAGCAAGTCTGGTAACGGTAACAACAATGGAAGTGACAGTGACAGTGACAGTGAAGGCAGTGACAGTAACCACTCAACCAGTGATGATTAG

Hap20b tgacagccagagcaagtctggtaacggtaacaacaatggaagtgacagtgacagtgacagtgaaggcagtgacagtaaccactcaaccagtgatgattag

Hap36a TGACAGCCAGAGCAAGTCTGGTAACGGTAACAACAATGGAAGTGACAGTGACAGTGACAGTGAAGGCAGTGACAGTAACCACTCAACCAGTGATGATTAG

Hap37a TGACAGCCAGAGCAAGTCTGGTAACGGTAACAACAATGGAAGTGACAGTGACAGTGACAGTGAAGGCAGTGACAGTAACCACTCAA**G**CAGTGATGATTAG

Hap38a TGACAGCCAGAGCAAGTCTGGTAACGGTAACAACAATGGAAGTGACAGTGACAGTGACAGTGAAGGCAGTGACAGTAACCACTCAACCAGTGATGATTAG

SHap1: tgacagccagagcaagtctggtaacggtaacaacaatggaagtgacagtgacagtgacagtgaaggcagtgacagtaaccactcaaccagtgatgattag

SHap2: tgacagccagagcaagtctggtaacggtaacaacaatggaagtgacagtgacagtgacagtgaaggcagtgacagtaaccactcaaccagtgatgattag

SHap3: tgacagccagagcaagtctggtaacggtaacaacaatggaagtgacagtgacagtgacagtgaaggcagtgacagtaaccactcaaccagtgatgattag

SHap4: tgacagccagagcaagtctggtaacggtaacaacaatggaagtgacagtgacagtgacagtgaaggcagtgacagtaaccactcaaccagtgatgattag

SHap5: tgacagccagagcaagtctggtaacggtaacaacaatggaagtgacagtgacagtgacagtgaaggcagtgacagtaaccactcaaccagtgatgattag

SHap6: tgacagccagagcaagtctggtaacggtaacaacaatggaagtgacagtgacagtgacagtgaaggcagtgacagtaaccactcaaccagtgatgattag

SHap6(2) tgacagccagagcaagtctggtaacggtaacaacaatggaagtgacagtgacagtgacagtgaaggcagtgacagtaaccactcaaccagtgatgattag

SHap7 tgacagccagagcaagtctggtaacggtaacaacaatggaagtgacagtgacagtgacagtgaaggcagtgacagtaaccactcaaccagtgatgattag

SHap102 tgacagccagagcaagtctggtaacggtaacaacaatggaagtgacagtgacagtgacagtgaaggcagtgacagtaaccactcaaccagtgatgattag

SHap130 tgacagccagagcaagtctggtaacggtaacaacaatggaagtgacagtgacagtgacagtgaaggcagtgacagtaaccactcaaccagtgatgattag

SHap106 tgacagccagagcaagtctggtaacggtaacaacaatggaagtgacagtgacagtgacagtgaaggcagtgacagtaaccactcaaccagtgatgattag

SHap72 tgacagccagagcaagtctggtaacggtaacaacaatggaagtgacagtgacagtgacagtgaaggcagtgacagtaaccactcaaccagtgatgattag

SHap110 tgacagccagagcaagtctggtaacggtaacaacaatggaagtgacagtgacagtgacagtgaaggcagtgacagtaaccactcaaccagtgatgattag

Hap4 tgacagccagagcaagtctggtaacggtaacaacaatggaagtgacagtgacagtgacagtgaaggcagtgacagtaaccactcaaccagtgatgattag

Hap5 tgacagccagagcaagtctggtaacggtaacaacaatggaagtgacagtgacagtgacagtgaaggcagtgacagtaaccactcaaccagtgatgattag

Hap8 tgacagccagagcaagtctggtaacggtaacaacaatggaagtgacagtgacagtgacagtgaaggcagtgacagtaaccactcaaccagtgatgattag

Hap13 tgacagccagagcaagtctggtaacggtaacaacaatggaagtgacagtgacagtgacagtgaaggcagtgacagtaaccactcaaccagtgatgattag

Hap24 tgacagccagagcaagtctggtaacggtaacaacaatggaagtgacagtgacagtgacagtgaaggcagtgacagtaaccactcaaccagtgatgattag

Hap25 tgacagccagagcaagtctggtaacggtaacaacaatggaagtgacagtgacagtgacagtgaaggcagtgacagtaaccactcaaccagtgatgattag

Hap26 tgacagccagagcaagtctggtaacggtaacaacaatggaagtgacagtgacagtgacagtgaaggcagtgacagtaaccactcaaccagtgatgattag

Hap29 tgacagccagagcaagtctggtaacggtaacaacaatggaagtgacagtgacagtgacagtgaaggcagtgacagtaaccactcaaccagtgatgattag

Hap32 tgacagccagagcaagtctggtaacggtaacaacaatggaagtgacagtgacagtgacagtgaaggcagtgacagtaaccactcaaccagtgatgattag

Hap33 tgacagccagagcaagtctggtaacggtaacaacaatggaagtgacagtgacagtgacagtgaaggcagtgacagtaaccactcaaccagtgatgattag

Hap35 tgacagccagagcaagtctggtaacggtaacaacaatggaagtgacagtgacagtgacagtgaaggcagtgacagtaaccactcaaccagtgatgattag

Merged tgacagccagagcaagtctggtaacggtaacaacaatggaagtgacagtgacagtgacagtgaaggcagtgacagtaaccactcaaccagtgatgattag

Transl D S Q S K S G N G N N N G S D S D S D S E G S D S N H S T S D D *

**References:**

1 Chen, S., Unterbrink, A., Kadapakkam, S., Dong, J., Gu, T.T., Dickson, J., Chuang, H.H. and MacDougall, M. (2004) Regulation of the Cell Type-specific dentin sialophosphoprotein gene expression in mouse odontoblasts by a novel transcription repressor and an activator CCAAT-binding factor. *J Biol Chem*, **279**, 42182-42191.

2 McKnight, D.A., Suzanne Hart, P., Hart, T.C., Hartsfield, J.K., Wilson, A., Wright, J.T. and Fisher, L.W. (2008) A comprehensive analysis of normal variation and disease-causing mutations in the human DSPP gene. *Hum Mutat*, **29**, 1392-1404.

3 McKnight, D.A., Simmer, J.P., Hart, P.S., Hart, T.C. and Fisher, L.W. (2008) Overlapping DSPP mutations cause dentin dysplasia and dentinogenesis imperfecta. *J Dent Res.*, **87**, 1108-1111.

4 Song, Y.L., Wang, C.N., Fan, M.W., Su, B. and Bian, Z. (2008) Dentin phosphoprotein frameshift mutations in hereditary dentin disorders and their variation patterns in normal human population. *J Med Genet*, **45**, 457-464.

5 Nieminen, P., Papagiannoulis-Lascarides, L., Waltimo-Siren, J., Ollila, P., Karjalainen, S., Arte, S., Veerkamp, J., Walton, V.T., Kustner, E.C., Siltanen, T. *et al.* (2011) Frameshift mutations in dentin phosphoprotein and dependence of dentin disease phenotype on mutation location. *J Bone Miner Res*, **26**, 873-880.

6 Lee, K.E., Kang, H.Y., Lee, S.K., Yoo, S.H., Lee, J.C., Hwang, Y.H., Nam, K.H., Kim, J.S., Park, J.C. and Kim, J.W. (2011) Novel dentin phosphoprotein frameshift mutations in dentinogenesis imperfecta type II. *Clin Genet*, **79**, 378-384.

**Fig. S3. Patterns of 32 DPP indels in *DSPP* haplotypes.** Indels highlighted in magenta are found in only a single haplotype; “na” means sequence not available. There are 36 different indel patterns: F2b, F4a, Hap1a, Hap2a, Hap3a, Hap15a, Hap17b:, Hap20a, Hap20b, Hap36a, Hap37a, Hap38a, SHap1, SHap2 (RefSeq), SHap3:, SHap4:, SHap5:, SHap6:, SHap6(2), SHap7, SHap102, SHap130, SHap106, SHap72, SHap110, Hap4, Hap5, Hap8, Hap13, Hap22, Hap24, Hap25, Hap26, Hap29, Hap32, Hap33, Hap35. Excluding the 16 indels that have been identified in only a single haplotype, there are 16 DPP indels that give rise to 25 confirmed (Fig. S4) *DSPP* haplotypes. The 11 haplotypes highlighted in magenta contain the 16 unconfirmed indels. The McKnight sequence is the same as F1a, which contains a frameshift mutation in haplotype SHap6(2). F1 through F5 are the haplotypes characterized by SMRT sequencing in the probands from our 5 families. F1b = Hap1a; F2a = SHap3; F3a = Hap15a; F3b = Hap2a; F4b = SHap2; F5a = Hap2a; F5b = Hap15a.

**Indels** ***ID1*** ***ID2 ID3 ID4 ID5 ID6 ID7 ID8 ID9 ID10 ID11 ID12 ID13 ID14 ID15 ID16 ID17 ID18 ID19 ID20 ID21 ID22 ID23 ID24 ID25 ID26 ID27 ID28 ID29 ID30 ID31 ID32***

RefSeq

McKnight ID2 ID5 ID8 ID14 ID15 ID21 ID23

F1a ID2 ID5 ID8 ID14 ID15 ID21 ID23

F1b ID2 ID3 ID8 ID14 ID15 ID21 ID23

F2a ID8 ID14 ID21 ID23

F2b ID11

F3a ID8 ID14 ID21 ID23 ID24

F3b ID14 ID21

F4a ID4 ID6 ID8 ID13 ID15 ID18 ID19 ID21 ID23 ID25

F4b

F5a ID14 ID21

F5b ID8 ID14 ID21 ID23 ID24

Hap1a ID2 ID3 ID8 ID14 ID15 ID21 ID23

Hap2a ID14 ID21

Hap3a ID27

Hap15a ID8 ID14 ID21 ID23 ID24

Hap17b: ID2 ID5 ID8 ID14 ID21 ID23 ID24

Hap20a ID8 ID9 ID14 ID21 ID28 ID32

Hap20b ID8 ID14 ID21

Hap36a ID1 ID8 ID9 ID21 ID28 ID32

Hap37a ID7 ID8 ID10 ID12 ID14 ID15 ID21 ID23

Hap38a ID8 ID9 ID15 ID21 ID30 ID32

SHap1: ID2 ID8 ID14 ID21 ID23

SHap2:

SHap3: ID8 ID14 ID21 ID23

SHap4: ID14 ID21 ID23

SHap5: ID2 ID5 ID8 ID14 ID15 ID21 ID23 ID28

SHap6: ID2 ID8 ID14 ID15 ID21 ID23

SHap6(2) ID2 ID5 ID8 ID14 ID15 ID21 ID23

SHap7 ID14 ID15 ID21 ID23

SHap102 ID8 ID14 ID21 ID23 ID28 ID32

SHap130 ID2 ID8 ID14 ID15 ID16 ID21 ID23

SHap106 ID2 ID8 ID14 ID15 ID17 ID21 ID23

SHap72 ID8 ID14 ID21 ID24 ID26

SHap110 ID14 ID21 ID23 ID28

Hap4 na na na na na na ID8 ID14 ID21 ID26 ID28 ID32

Hap5 na na na na na na ID8 ID9 ID14 ID21 ID26 ID28 ID32

Hap8 na na na na na na ID8 ID14 ID21 ID23 ID28

Hap13 na na na na na na ID8 ID14 ID21 ID22 ID23 ID24 ID29

Hap24 na na na na na na ID20

Hap25 na na na na na na ID8

Hap26 na na na na na na ID8 ID10 ID12 ID14 ID15 ID21 ID23 ID29

Hap29 na na na na na na ID8 ID12 ID14 ID15 ID21 ID23

Hap32 na na na na na na ID8 ID9 ID15 ID21 ID28 ID32

Hap33 na na na na na na ID8 ID14 ID15 ID21 ID23 ID31

Hap35 na na na na na na ID8 ID14 ID15 ID21 ID23

**Fig. S4.** Map showing the 25 different patterns of confirmed indels (haplotypes). Novel indels (those only found in only a single haplotype) were deleted (as potential artifacts) and then duplicate haplotypes patterns were deleted. Note that Indels 14, 15, 23, and 28 cannot be grouped without ungrouping other indels. This suggests that multiple indels in the DPP repetitive region may have been generated independently in different haplotypes.

**Indels** ***ID2 ID3 ID5 ID8 ID9 ID12 ID14 ID15 ID21 ID23 ID24 ID26 ID28 ID32***

SHap2 (RefSeq)

Hap20b ID14

Hap25 na na na ID8

SHap102 ID8 ID14 ID21 ID23 ID28 ID32

Hap32 na na na ID8 ID9 ID15 ID21 ID28 ID32

Hap20a ID8 ID9 ID14 ID21 ID28 ID32

Hap5 na na na ID8 ID9 ID14 ID21 ID26 ID28 ID32

Hap4 na na na ID8 ID14 ID21 ID26 ID28 ID32

SHap72 ID8 ID14 ID21 ID24 ID26

Hap17b ID2 ID5 ID8 ID14 ID21 ID23 ID24

Hap15a ID8 ID14 ID21 ID23 ID24

Hap1a ID2 ID3 ID8 ID14 ID15 ID21 ID23

SHap6: ID2 ID8 ID14 ID15 ID21 ID23

SHap6(2) ID2 ID5 ID8 ID14 ID15 ID21 ID23

SHap5: ID2 ID5 ID8 ID14 ID15 ID21 ID23 ID28

Hap8 na na na ID8 ID14 ID21 ID23 ID28

SHap1: ID2 ID8 ID14 ID21 ID23

SHap3: ID8 ID14 ID21 ID23

Hap26 na na na ID8 ID12 ID14 ID15 ID21 ID23

Hap29 na na na ID8 ID12 ID14 ID15 ID21 ID23

Hap35 na na na ID8 ID14 ID15 ID21 ID23

SHap7 ID14 ID15 ID21 ID23

SHap110 ID14 ID21 ID23 ID28

SHap4: ID14 ID21 ID23

Hap2a ID14 ID21
