# Supplementary material for: The dentin phosphoprotein repeat region and inherited defects of dentin
Source: Mol Genet Genomic Med. 2015 Sep 7;4(1):28–38. doi: 10.1002/mgg3.176 (PMC4707025; doi:10.1002/mgg3.176)
Supplement: Supplementary file 2 — Table S1. Outcome analyses of whole‐exome sequencing. Table S2. PCR primers and reaction conditions. Table S3A. Thirty‐eight short DSPP haplotype sequences in NCBI PopSet: 162077127. Table S3B. Twenty‐one longer DSPP haplotype sequences in NCBI PopSet: 162077085. Table S4. List of insertions and deletions (indels) in the DPP repetitive region. Table S5. Disease‐causing mutations in the coding region for DPP. [file MGG3-4-028-s002.docx]

**Table S1. Outcome analyses of whole exome sequencing.**

| Symptom | DGI-II | DGI-II | OI-DGI |
| --- | --- | --- | --- |
| Genetic status | Singleton | Singleton | Singleton |
| Sample | Family 2 | Family 3 | Family 5 |
| Sex | M | M | F |
| Assumed inheritance pattern | Rec/Dom | Rec/Dom | Rec/Dom |
| Lanes used | 1/6 | 1/6 | 1/6 |
| SE/PE | PE | PE | PE |
| Read length | 74 | 74 | 74 |
| # of reads (M) | 70 | 94.3 | 91.1 |
| Median coverage (X) | 57 | 81 | 82 |
| Mean coverage (X) | 67.5 | 91.4 | 93.5 |
| % on genome | 92.35% | 92.41% | 92.25% |
| % on target | 62.62% | 62.87% | 66.70% |
| % of bases covered at least 4x | 97.18% | 97.90% | 97.87% |
| % of bases covered at least 8x | 95.00% | 96.60% | 96.69% |
| Mean error rate | 0.40% | 0.37% | 0.43% |
| 1st reads error rate | 0.36% | 0.35% | 0.40% |
| 2nd reads error rate | 0.43% | 0.39% | 0.46% |
| 2nd base error rate | 0.38% | 0.36% | 0.41% |
| Last base error rate | 0.76% | 0.71% | 1.03% |
| % of PCR duplicate | 2.70% | 2.97% | 4.15% |
| # of Hom SNVs QS>60 | 12108 | 12924 | 12183 |
| # of novel Hom SNVs QS>60 | 32 | 23 | 21 |
| # of novel-novel Hom SNVs QS>60 | 19 | 15 | 15 |
| % of novel Hom SNVs QS>60 | 0.26% | 0.18% | 0.17% |
| # of Het SNVs QS>100 | 20056 | 20265 | 20905 |
| # of novel Het SNVs QS>100 | 414 | 477 | 750 |
| % of novel Het SNVs QS>100 | 2.06% | 2.35% | 3.59% |
| # of Het SNVs QS>200 | 15609 | 17128 | 17900 |
| # of novel Het SNVs QS>200 | 271 | 330 | 611 |
| % of novel Het SNVs QS>200 | 1.74% | 1.93% | 3.41% |

**Key to abbreviations:** Dom, dominant; Het, heterozygous; Hom, homozygous; M, million bases; PE, paired-end reads; QS: quality score; Rec, recessive; SE: single-end reads; SNVs; single nucleotide variants, X, fold.

**Table S2. PCR primers and reaction conditions.**

| Gene | Primer Name and Sequence | Anneal  Temp | Amplicon  Size |
| --- | --- | --- | --- |
| DSPPex1 | DSPP1F: 5’TCACCAAGTGAAGGAAGTGG  DSPP1R: 5’AAGCCCAAGGTGGATTTTT | 56 ˚C | 565 bp |
| DSPex2 | DSP2F: 5’GATGTCCCCATAACCACACC  DSP2R: 5’CTCCATGACTTCTGGGCATT | 56 ˚C | 596 bp |
| DSPex3/4 | DSP34F: 5’CAAGCCCTGTAAGAAGCCACT  DSP34R: 5’ACATGGATGCTTGTCATGGT | 59 ˚C | 1490 bp |
| DSPex5 | DSP5F: 5’CCTATGGCAACTTTTCCCAGT  DSP5R: 5’TGTCATTGTCATCATTCCCATT | 56 ˚C | 589 bp |
| DPPF1 | DPPF1: 5’GCGCTCTGTGTGCAGCAGTCCATGCAAGGAGATGATCC  DPPR1: 5’GCGCTCTGTGTGCAGCCTAATCATCACTGGTTGAGTGG | 55 ˚C | ~2534 bp |
| DPPF2 | DPPF2: 5’TCATGAGTCGACACTAAGTCCATGCAAGGAGATGATCC  DPPR2: 5’TCATGAGTCGACACTACTAATCATCACTGGTTGAGTGG | 55 ˚C | ~2534 bp |
| DPPF3 | DPPF3: 5’TATCTATCGTATACGCAGTCCATGCAAGGAGATGATCC  DPPR3: 5’TATCTATCGTATACGCCTAATCATCACTGGTTGAGTGG | 55 ˚C | ~2534 bp |
| DPPF4 | DPPF4: 5’ATCACACTGCATCTGAAGTCCATGCAAGGAGATGATCC  DPPR4: 5’ATCACACTGCATCTGACTAATCATCACTGGTTGAGTGG | 55 ˚C | ~2534 bp |
| DPPF5 | DPPF5: 5’ACGTACGCTCGTCATAAGTCCATGCAAGGAGATGATCC  DPPR5: 5’ACGTACGCTCGTCATACTAATCATCACTGGTTGAGTGG | 55 ˚C | ~2534 bp |
| *COL1A2*  p.G676D | A2X34F: 5’TCCAACCAGAGTGCAGTGAA  A2X34R: 5’GGAAGGCGGGAAATTTTAGA | 58 ˚C | 229 bp |

DPP PCR reactions conditions: 5 min at 94˚C followed by 35 cycles of denaturing at 94˚C for 30 sec, annealing at 55 ˚C for 30 s and extension at 73 ˚C for 3 min, and a final extension at 72 ˚C for 5 min.

**Table S3A. 38 Short DSPP haplotype sequences in NCBI PopSet: 162077127.**

EU278672.1 Human haplotype 33 dentin sialophosphoprotein (*DSPP*) gene, exon 5 and partial cds 1092 bp.

EU278676.1 Human haplotype 37 dentin sialophosphoprotein (*DSPP*) gene, exon 5 and partial cds 1140 bp.

EU278652.1 Human haplotype 13 dentin sialophosphoprotein (*DSPP*) gene, exon 5 and partial cds 1155 bp.

EU278665.1 Human haplotype 26 dentin sialophosphoprotein (*DSPP*) gene, exon 5 and partial cds 1155 bp.

EU278643.1 Human haplotype 4 dentin sialophosphoprotein (*DSPP*) gene, exon 5 and partial cds 1164 bp.

EU278677.1 Human haplotype 38 dentin sialophosphoprotein (*DSPP*) gene, exon 5 and partial cds 1164 bp.

EU278644.1 Human haplotype 5 dentin sialophosphoprotein (*DSPP*) gene, exon 5 and partial cds 1173 bp.

EU278645.1 Human haplotype 6 dentin sialophosphoprotein (*DSPP*) gene, exon 5 and partial cds 1173 bp.

EU278668.1 Human haplotype 29 dentin sialophosphoprotein (*DSPP*) gene, exon 5 and partial cds 1173 bp.

EU278641.1 Human haplotype 2 dentin sialophosphoprotein (*DSPP*) gene, exon 5 and partial cds 1182 bp.

EU278654.1 Human haplotype 15 dentin sialophosphoprotein (*DSPP*) gene, exon 5 and partial cds 1182 bp.

EU278656.1 Human haplotype 17 dentin sialophosphoprotein (*DSPP*) gene, exon 5 and partial cds 1182 bp.

EU278657.1 Human haplotype 18 dentin sialophosphoprotein (*DSPP*) gene, exon 5 and partial cds 1182 bp.

EU278649.1 Human haplotype 10 dentin sialophosphoprotein (*DSPP*) gene, exon 5 and partial cds 1182 bp.

EU278650.1 Human haplotype 11 dentin sialophosphoprotein (*DSPP*) gene, exon 5 and partial cds 1182 bp.

EU278651.1 Human haplotype 12 dentin sialophosphoprotein (*DSPP*) gene, exon 5 and partial cds 1182 bp.

EU278659.1 Human haplotype 20 dentin sialophosphoprotein (*DSPP*) gene, exon 5 and partial cds 1182 bp.

EU278662.1 Human haplotype 23 dentin sialophosphoprotein (*DSPP*) gene, exon 5 and partial cds 1182 bp.

EU278667.1 Human haplotype 28 dentin sialophosphoprotein (*DSPP*) gene, exon 5 and partial cds 1182 bp.

EU278670.1 Human haplotype 31 dentin sialophosphoprotein (*DSPP*) gene, exon 5 and partial cds 1182 bp.

EU278671.1 Human haplotype 32 dentin sialophosphoprotein (*DSPP*) gene, exon 5 and partial cds 1182 bp.

EU278673.1 Human haplotype 34 dentin sialophosphoprotein (*DSPP*) gene, exon 5 and partial cds 1182 bp.

EU278640.1 Human haplotype 1 dentin sialophosphoprotein (*DSPP*) gene, exon 5 and partial cds 1191 bp.

EU278646.1 Human haplotype 7 dentin sialophosphoprotein (*DSPP*) gene, exon 5 and partial cds 1191 bp.

EU278647.1 Human haplotype 8 dentin sialophosphoprotein (*DSPP*) gene, exon 5 and partial cds 1191 bp.

EU278655.1 Human haplotype 16 dentin sialophosphoprotein (*DSPP*) gene, exon 5 and partial cds 1191 bp.

EU278674.1 Human haplotype 35 dentin sialophosphoprotein (*DSPP*) gene, exon 5 and partial cds 1191 bp.

EU278666.1 Human haplotype 27 dentin sialophosphoprotein (*DSPP*) gene, exon 5 and partial cds 1200 bp.

EU278669.1 Human haplotype 30 dentin sialophosphoprotein (*DSPP*) gene, exon 5 and partial cds 1200 bp.

EU278675.1 Human haplotype 36 dentin sialophosphoprotein (*DSPP*) gene, exon 5 and partial cds 1200 bp.

EU278658.1 Human haplotype 19 dentin sialophosphoprotein (*DSPP*) gene, exon 5 and partial cds 1209 bp.

EU278660.1 Human haplotype 21 dentin sialophosphoprotein (*DSPP*) gene, exon 5 and partial cds 1209 bp.

EU278661.1 Human haplotype 22 dentin sialophosphoprotein (*DSPP*) gene, exon 5 and partial cds 1209 bp.

EU278648.1 Human haplotype 9 dentin sialophosphoprotein (*DSPP*) gene, exon 5 and partial cds 1335 bp.

EU278653.1 Human haplotype 14 dentin sialophosphoprotein (*DSPP*) gene, exon 5 and partial cds 1335 bp.

EU278664.1 Human haplotype 25 dentin sialophosphoprotein (*DSPP*) gene, exon 5 and partial cds 1344 bp.

EU278642.1 Human haplotype 3 dentin sialophosphoprotein (*DSPP*) gene, exon 5 and partial cds 1353 bp.

EU278663.1 Human haplotype 24 dentin sialophosphoprotein (*DSPP*) gene, exon 5 and partial cds 1524 bp.

**Table S3B. 21 Longer *DSPP* haplotype sequences in NCBI PopSet: 162077085.**

EU278619.1 Human haplotype 1E dentin sialophosphoprotein (*DSPP*) gene, exon 5 and partial cds 2301 bp.

EU278620.1 Human haplotype 1A dentin sialophosphoprotein (*DSPP*) gene, exon 5 and partial cds 2301 bp.

EU278621.1 Human haplotype 1B dentin sialophosphoprotein (*DSPP*) gene, exon 5 and partial cds 2316 bp.

EU278622.1 Human haplotype 1D dentin sialophosphoprotein (*DSPP*) gene, exon 5 and partial cds 2301 bp.

EU278623.1 Human haplotype 1F dentin sialophosphoprotein (*DSPP)* gene, exon 5 and partial cds 2319 bp.

EU278624.1 Human haplotype 1C dentin sialophosphoprotein (*DSPP*) gene, exon 5 and partial cds 2319 bp.

EU278625.1 Human haplotype 2B dentin sialophosphoprotein (*DSPP*) gene, exon 5 and partial cds 2292 bp.

EU278626.1 Human haplotype 2D dentin sialophosphoprotein (*DSPP*) gene, exon 5 and partial cds 2292 bp.

EU278627.1 Human haplotype 2A dentin sialophosphoprotein (*DSPP*) gene, exon 5 and partial cds 2292 bp.

EU278628.1 Human haplotype 3A dentin sialophosphoprotein (*DSPP*) gene, exon 5 and partial cds 2463 bp.

EU278638.1 Human haplotype 14A dentin sialophosphoprotein (*DSPP*) gene, exon 5 and partial cds 2445 bp.

EU278629.1 Human haplotype 15A dentin sialophosphoprotein (*DSPP*) gene, exon 5 and partial cds 2292 bp.

EU278630.1 Human haplotype 17A dentin sialophosphoprotein (*DSPP*) gene, exon 5 and partial cds 2292 bp.

EU278631.1 Human haplotype 17B dentin sialophosphoprotein (*DSPP*) gene, exon 5 and partial cds 2307 bp.

EU278632.1 Human haplotype 20A dentin sialophosphoprotein (*DSPP*) gene, exon 5 and partial cds 2292 bp.

EU278633.1 Human haplotype 20B dentin sialophosphoprotein (*DSPP*) gene, exon 5 and partial cds 2310 bp.

EU278634.1 Human haplotype 21A dentin sialophosphoprotein (*DSPP*) gene, exon 5 and partial cds 2337 bp.

EU278635.1 Human haplotype 21B dentin sialophosphoprotein (*DSPP*) gene, exon 5 and partial cds 2319 bp.

EU278636.1 Human haplotype 36A dentin sialophosphoprotein (*DSPP*) gene, exon 5 and partial cds 2304 bp.

EU278637.1 Human haplotype 37A dentin sialophosphoprotein (*DSPP*) gene, exon 5 and partial cds 2238 bp.

EU278639.1 Human haplotype 38A dentin sialophosphoprotein (*DSPP*) gene, exon 5 and partial cds 2274 bp.

**Table S4. List of insertions and deletions (indels) in the DPP repetitive region.** Indel numbers highlighted in magenta are currently found in only a single haplotype.

***ID1***: NM_014208.3:c.2035_2040delAGTAGC.

***ID2***: NM_014208.3:c.2053_2054insGTAGCAGTGACAGCAGCA.

***ID3:*** NM_014208.3:c.2214_2231delCAACAGCAGTGACAGCAG.

***ID4:*** NM_014208.3:c.2286_2303delCAACAGCAGTGACAGCAG.

***ID5:*** NM_014208.3:c.2507_2509delGCA.

***ID6:*** NM_014208.3:c.2509_2526delAACAGCAGTGATAGCAGC.

***ID7:*** NM_014208.3:c.2569_2604delAATAGAAGTGACAGTAGTAATAGTAGTGACAGCAGC.

***ID8:*** NM_014208.3:c.2645_2646insTAGTGACAG.

***ID9:*** NM_014208.3:c.2737_2745delGACAGCAGT.

***ID10:*** NM_014208.3:c.2836_2844delGATAGCAGT.

***ID11:*** NM_014208.3:c.2877_2906dup

Same as designation: NM_014208.3:c.2906_2907insCAACAGCAGTGACAGCAG.

***ID12:*** NM_014208.3:c.2958_2975delTGACAGCAGTGATAGCAG.

***ID13:*** NM_014208.3:c.2976_3002dup

Same as designation: NM_014208.3:c.3002_3003insTGACAGCAGTGACAGCAGTGATAGCAG.

***ID14:*** NM_014208.3:c.3069_3086deltagcagtgacagcagcaa.

***ID15:*** NM_014208.3:c.3136_3153delAGCGATAGCAGTGACAGC.

***ID16:*** NM_014208.3:c.3156_3164delTGACAGCAG.

***ID17:*** NM_014208.3:c.3170_3196dup

Same as designation: NM_014208.3:c.3196_3197insGCAGTGACAGCAGTGACAGCAGCGACA.

***ID18:*** NM_014208.3:c.3219_3227delTGACAGCAG.

***ID19:*** NM_014208.3:c.3257_3445delATAGCAGTGATAGCAGTGACAGCAGCAA CAGCAGTGACAGCAGTGACAGCAGTG.

***ID20:*** NM_014208.3:c.3265_3266insacagcagcaatagcagtgacagcagtgacagcagcgacagca

gtgatagcagtgacagcagcgatagcagtgacagcagtgacagcagcaatagcagtgacagcagtgacagcagcgacagcagtgatagcagtgacagcagtgacagcagcgacagcagtgatagcagtgaaagcagtgatagcagtg.

***ID21:*** NM_014208.3:c.3266_3400delACAGCAGCAATAGCAGTGACAGCAGCGATAGCAGCGA

CAGCAGCGACAGCAGCGATAGCAGTGACAGCAGCGATAGCAGTGACAGCAGTGACAGCAGCAATAGCAGTGACAGCAGTGACAGCAGCGACAGCAGTG.

***ID22:*** NM_014208.3:c.3430_3447delAGCAGTGACAGCAGTGAA.

***ID23:*** NM_014208.3:c.3447_3448insAGCAGCGACAGCAGCGAT.

***ID24:*** NM_014208.3:c.3466_3492delAGCAGCGACAGCAGTGACAGCAGCGAT

***ID25:*** NM_014208.3:c.3509_3517delACAGCAGCG.

***ID26:*** NM_014208.3:c.3591_3599delTAGCAGCGA.

***ID27:*** NM_014208.3:c.3594_3611dupCAGCGACAGCAGCGATAG.

***ID28:*** NM_014208.3:c.3624_3641delTGACAGCAGTGACAGCAG.

***ID29:*** NM_014208.3:c.3633_3641delTGACAGCAG.

***ID30:*** NM_014208.3:c.3624_3659delTGACAGCAGTGACAGCAGCGACAGCAGTGACAGCAG

***ID31:*** NM_014208.3:c.3654_3752delCAGCAGCGACAGCAGTGACAGCAGCGACAGCAGTGACAGCA

ATGAAAGCAG CGACAGCAGTGACAGCAGCGATAGCAGTGACAGCAGCAACAGCAGTGA

***ID32:*** NM_014208.3:c.3724_3741dup

Same as designation: NM_014208.3:c.3741_3742insGATAGCAGTGACAGCAGC.

**Table S5. Disease-causing mutations in the coding region for DPP.** All 21 disease-causing mutations shift the reading frame into the -1 reading frame. (Cyan: Dentin dysplasia II; Green: Dentinogenesis imperfecta type II).

1. Dentin dysplasia II: c.1686delT; p.Asp562Glufs*752 (1) Kindred 1

2. Dentin dysplasia II: c.1830delC; p.Ser610Argfs*704 (1) Kindred 2

3. Dentin dysplasia II: c.1870_1873delTCAG; p.Ser624Thrfs*689 (2)

4. Dentin dysplasia II: c.1918_1921delTCAG; p.Ser640Thrfs*673 (2)

5. Dentin dysplasia II: c.1918_1921delTCAG; p.Ser640Thrfs*673 (1) Kindreds 3-5

6. Dentin dysplasia II: c.1922_1925delACAG; p.Asp641Alafs*672 (1) Kindred 6

7. Dentin dysplasia II: c.2040delC; p.Ser680Argfs*634 (3) Family H

8. Dentin dysplasia II: c.2063delA; p.Asp688Valfs*626 (1) Kindreds 7 & 8

9. Dentinogenesis imperfecta type II: c.2272delA; p.Ser758Alafs*556 (2)

10. Dentinogenesis imperfecta type II: c.2349delT; p.Ser783Argfs*531 (1) Kindred 9

11. Dentinogenesis imperfecta type II: c.2525delG; p.Ser842Thrfs*472 (2)

12. Dentinogenesis imperfecta type II: c.2593delA; p.Ser865Valfs*449 (3) Family J.

13. Dentinogenesis imperfecta type II: c.2666delG; p.Ser889Thrfs*425 (1) Kindred 10.

14. Dentinogenesis imperfecta type II: c.2684delG; p.Ser895Metfs*419 (3) Family S.

15. Dentinogenesis imperfecta type II: c.2688delT; p.Asp896Glufs*418 (4)

16. Dentin Dysplasia II: c.3135delC; p.Ser1045Argfs*269. (5)

17. Dentinogenesis imperfecta type II: c.3438delC; p.Asp1146Glufs*168 (3) Family E.

18. Dentinogenesis imperfecta type II: c.3504_3508dup; p.Asp1170Alafs*146 This Study Family 2

19. Dentinogenesis imperfecta type II: c.3546_3550delTAGCAinsG; p.Asp1182Glufs*131 (3) Family M

20. Dentinogenesis imperfecta type II: c.3560delG; p.Ser1187Metfs*127 (4)

21. Dentin dysplasia II: c.3582_3591delCAGCAGCGAT; p.Asp1194Glufs*117 (1) Kindred 11

**References.**

1 Nieminen, P., Papagiannoulis-Lascarides, L., Waltimo-Siren, J., Ollila, P., Karjalainen, S., Arte, S., Veerkamp, J., Walton, V.T., Kustner, E.C., Siltanen, T. *et al.* (2011) Frameshift mutations in dentin phosphoprotein and dependence of dentin disease phenotype on mutation location. *J Bone Miner Res*, **26**, 873-880.

2 McKnight, D.A., Suzanne Hart, P., Hart, T.C., Hartsfield, J.K., Wilson, A., Wright, J.T. and Fisher, L.W. (2008) A comprehensive analysis of normal variation and disease-causing mutations in the human DSPP gene. *Hum Mutat*, **29**, 1392-1404.

3 Song, Y.L., Wang, C.N., Fan, M.W., Su, B. and Bian, Z. (2008) Dentin phosphoprotein frameshift mutations in hereditary dentin disorders and their variation patterns in normal human population. *J Med Genet*, **45**, 457-464.

4 Lee, K.E., Kang, H.Y., Lee, S.K., Yoo, S.H., Lee, J.C., Hwang, Y.H., Nam, K.H., Kim, J.S., Park, J.C. and Kim, J.W. (2011) Novel dentin phosphoprotein frameshift mutations in dentinogenesis imperfecta type II. *Clin Genet*, **79**, 378-384.

5 McKnight, D.A., Simmer, J.P., Hart, P.S., Hart, T.C. and Fisher, L.W. (2008) Overlapping DSPP mutations cause dentin dysplasia and dentinogenesis imperfecta. *J Dent Res.*, **87**, 1108-1111.
